# Tubular retractors in neuro-oncological surgery: a systematic review and meta-analysis

**DOI:** 10.1007/s10143-025-03677-w

**Published:** 2025-06-27

**Authors:** Amir Rafati Fard, Owen Hibberd, Isaac Akinduro, Zainab Bhatti, Kieran J. Smith, Reece Patel, Sejal Karmarkar, Oliver D. Mowforth, Ciaran S. Hill

**Affiliations:** 1 School of Clinical Medicine, University of Cambridge, Cambridge, UK; 2 School of Medicine, University of Nottingham, Nottingham, UK; 3 Division of Neurosurgery, Department of Clinical Neurosciences, University of Cambridge, Cambridge, UK; 4 Victor Horsley Department of Neurosurgery, National Hospital for Neurology and Neurosurgery, University College London NHS Trust, London, UK; 5 UCL Cancer Institute, University College London, London, UK

**Keywords:** Minimally invasive, Neuro-oncology, Resection, Safety, Surgery, Tubular retractors, Tumour

## Abstract

Neuro-oncological surgery necessitates a careful balance between maximising tumour resection whilst minimising damage to healthy brain parenchyma. Tubular retractors represent an emerging tool proposed to facilitate in the optimisation of this onco-functional balance. The objective was to evaluate the evidence regarding tubular retractors in neuro-oncological surgery. A systematic review and meta-analysis was performed. Studies reporting on surgical outcomes of tubular retractors in adult neuro-oncological cases were eligible. Medline, Embase, Cochrane Library, ClinicalTrials.gov, and ICTRP were searched to 14th July 2024. Duplicate title/abstract screening, data extraction, and risk of bias assessments were conducted. Prevalence of gross total resection (GTR) and complications were calculated using random effects models. 49 studies were included in the final analysis with a total of 684 patients. Combined pooled prevalence for GTR was 76% (95% CI: 67–85%), whilst for complications was 14% (95% CI: 8–20%). GTR rate by tumour histology was: 52% for gliomas (95% CI: 41–62%), 80% for metastases (95% CI: 65–92%), and 100% for colloid cysts (95% CI: 99–100%). Complication rate by tumour histology was: 16% for gliomas (95% CI: 5–30%), 12% for metastases (95% CI: 1–28%), and 16% for colloid cysts (95% CI: 8–24%). There was no significant difference between tubular retractor brands and GTR or complication rate (*p* > 0.05). Despite the mounting interest regarding the utility of tubular retractors in neuro-oncological surgery, the current evidence remains largely in the form of case series. Prospective studies with greater sample sizes, longer follow-up, and direct comparison to conventional retraction are now needed.

**Supplementary Information:**

The online version contains supplementary material available at 10.1007/s10143-025-03677-w.

## Introduction

Brain tumours encompass a heterogenous group of benign and malignant lesions originating from the brain parenchyma and surrounding tissue or due to metastatic spread from other organs across the body [1]. Collectively, they represent a significant source of morbidity and mortality worldwide [2]. Despite development of novel neuro-oncological therapies [3], surgical resection remains the mainstay of treatment for many patients with a brain tumour [4]. Crucially, surgical resection necessitates a fine balance between the oncological advantage conferred by maximum resection against functional deficit from damage to nearby healthy tissue (termed the ‘onco-functional balance’) [5]. As such, maximal safe resection forms the current standard of care in neuro-oncological surgery [6]. 

Retraction of surrounding brain parenchyma is a key step in neuro-oncological surgery often required for adequate tumour visualisation and for creation of a surgical corridor. Conventionally, neurosurgeons have utilised metal blade retractors for retraction, but these tools apply a focal pressure to brain tissue that result in localised contusions and ischaemia with associated retraction-induced brain injury [7]. Such injury has been shown to be a significant source of patient morbidity, including focal neurological deficits, cognitive impairment, and post-operative seizures [8], with estimates of incidence in up to 10% of intracranial neurosurgical cases [9]. Consequently, in an attempt to minimize retraction-induced brain injury, there has been a shift to ‘dynamic retraction’ or ‘retractorless surgery’ [10]. However, in neuro-oncological surgery the complete avoidance of brain retraction is often challenging, particularly for deep-seated tumours.

First proposed by Kelly et al. in the 1980s [11], tubular retractors represent an emerging alternative to conventional retractors. By circumferentially distributing pressure across the brain parenchyma, tubular retractors theoretically minimise injury associated with retraction, whilst concomitantly providing improved visibility of both the tumour and surrounding tissue through a clear surgical corridor [12, 13]. Furthermore, through a minimally invasive approach, tubular retractors are able to potentially avoid disturbance to regions of the brain that may otherwise have been injured from the larger access required in conventional procedures. Therefore, by taking advantage of naturally existing anatomical corridors within the brain, tubular retractors can minimise injury to critical surrounding structures, such as subcortical white matter tracts as in minimally invasive parafascicular surgery (MIPS) [10]. MIPS has already been shown to be of potential clinical benefit in the management of patients with intracerebral haemorrhage across several multicentre studies [14–17], notably including the prospective ENRICH randomised trial [14], supporting its translational potential to neuro-oncological surgery. As such, tubular retractors are a promising tool in the neurosurgeon’s ever-expanding arsenal, necessitating their continual critical appraisal to determine their utility and scope in neuro-oncological surgery.

Whilst two previous systematic reviews have provided insight into the potential benefits of tubular retractors in the resection of brain lesions [12, 13], their search strategies are now five years out of date with the literature significantly expanded since. Furthermore, their analyses were broad, incorporating lesions beyond the subspecialty of neuro-oncology, including intracerebral haemorrhages and vascular malformations. As such, an update is now required to draw more concrete conclusions from the larger evidence base and to assess the current landscape of tubular retractors specifically in the field of neuro-oncological surgery. Our objective was to synthesise all evidence regarding the surgical outcomes of tubular retractors in neuro-oncological surgery. Through meta-analysis, we consider their efficacy and safety, and through sub-group analysis, we investigate their utility for specific tumour histology and compare different tubular retractor brands.

## Methods

This systematic review and meta-analysis was reported in accordance with the Preferred Reporting Items for Systematic Reviews and Meta-Analyses (PRISMA) guidelines (Supplementary Data 1) [18]. The protocol was registered on PROSPERO (ID: CRD42023464954).

### Search strategy and selection criteria

The search strategy was reviewed by a medical librarian at the University of Cambridge (IK). Searches were performed in Medline and Embase using Ovid (Wolters Kluwer, Netherlands), the Cochrane Library, ClinicalTrials.gov, and ICTRP from inception to 14th July 2024 (Supplementary Data 2). Search sensitivity was evaluated against 11 papers known to meet inclusion: all papers were successfully captured. Manual searches of reference lists and citations of included studies were undertaken to identify any additional relevant studies that might have been missed.

Primary studies that investigated a cohort of adult patients with a brain tumour (*Population*), utilised tubular retractors (*Intervention*), and reported on measures of efficacy and/or safety (*Outcomes*) were included. There were no specified comparators. Studies were excluded if the title and abstract indicated failure to meet all of the specified inclusion criteria or satisfied at least one of the exclusion criteria (Table 1). Six reviewers (ARF/OH/ZB/KJS/RP/SK) independently performed title and abstract screening with blinding using (Rayyan Systems Inc., Cambridge, USA). A pilot screen of 100 publications was conducted to ensure concordance between reviewers. Disagreements following unblinding were resolved by discussion between reviewers and the senior author (CSH) where necessary.

**Table 1:** Inclusion and exclusion criteria

Inclusion criteria	Exclusion criteria
• Primary study • Adult patients with a brain tumour (including cysts) • Resection using tubular retractor system	• Non-adult population (< 18 years) • Mixed cohort (e.g. spinal cord tumour, intracerebral haemorrhage) • Non-English language • Non-human study • Editorial, case report, opinion article, letter, conference abstract, correction • Systematic review or meta-analysis • Full text unavailable

### Data extraction and critical appraisal

Articles were retrieved for full text screening and data extraction using a piloted proforma. Data extracted from articles included: author, publication year, study design, selection criteria, sample characteristics, brain tumour characteristics, tubular retractor brand and dimensions, surgical workflow, efficacy outcomes, safety outcomes, main conclusion, and limitations. Extent of resection (EOR) was defined as follows: gross total resection (GTR) referred to an EOR of 100%, near total resection (NTR) was defined as an EOR between 95% and 99%, and subtotal resection (STR) was defined as an EOR < 95%. Where a study defined NTR differently (e.g. between 90 and 99%) and there was no further information regarding the exact EOR, these resections were allocated STR to avoid inflating our estimate of NTR cases.

Risk of methodological bias in individual studies was assessed using the Joanna Briggs Institute (JBI) critical assessment tools for case series or cohort studies. As advocated by JBI, no study was excluded because of risk of bias. Criteria 10 (appropriate statistical analysis) of the case series checklist was deemed not applicable to case series included in our review. Each study was assigned a score from 0 (no criteria satisfied) to 9 or 11 (all criteria satisfied for case series or cohort studies respectively), where a ‘Yes’ scored 1, ‘Unclear’ scored 0.5, and ‘No’ scored 0. We trichrotomised this ordinal scale to classify risk of bias as high (scores 0-2.5 or 0-3.5 for case series or cohort studies respectively), moderate (scores 3–6 or 4-7.5 for case series or cohort studies respectively), and low (scores 6.5-9 or 8–11 for case series or cohort studies respectively). Confidence in the body of evidence was assessed using the Grading of Recommendations, Assessment, Development, and Evaluations (GRADE) framework [19]. 

Full text screening, data extraction, risk of methodological bias, and confidence in the body of evidence were assessed independently in duplicate (ARF and OH/IA/KS) with blinding. Any disagreements following unblinding were resolved by discussion between reviewers until mutual agreement was reached. Outstanding questions were resolved by discussion with the senior reviewer (ODM) and the senior author (CSH) where necessary.

### Data analysis and reporting

Meta-analysis using a random effects model was performed for pooled proportion estimates for prevalence of GTR and complications in studies that had five or more patients eligible. Proportions were calculated for each of the outcome measures, where the denominator referred to the total number of patients within the study that met the inclusion criteria. Biopsies were excluded from the GTR meta-analysis. Subgroup analysis was performed based on tumour histology and brand of tubular retractor. Forest plots with 95% confidence intervals were generated using the Tukey-Freeman double arcsine transformation and DerSimonian-Laird variance estimator. Total heterogeneity and I^2^ characteristics were calculated. Heterogeneity was investigated using leave-one-out sensitivity analysis. Publication bias was evaluated and presented as funnel plots, with Egger’s test used to assess funnel plot symmetry. Differences in prevalence of GTR and complications between tumour histology and between brands of tubular retractor were compared using the Chi-squared test, where the null hypothesis was rejected for *p* < 0.05. Statistical analyses were performed using R (v4.2.2; R Core Team 2022) (‘meta’ and ‘ggplot2’ packages).

## Results

**Fig. 1:**
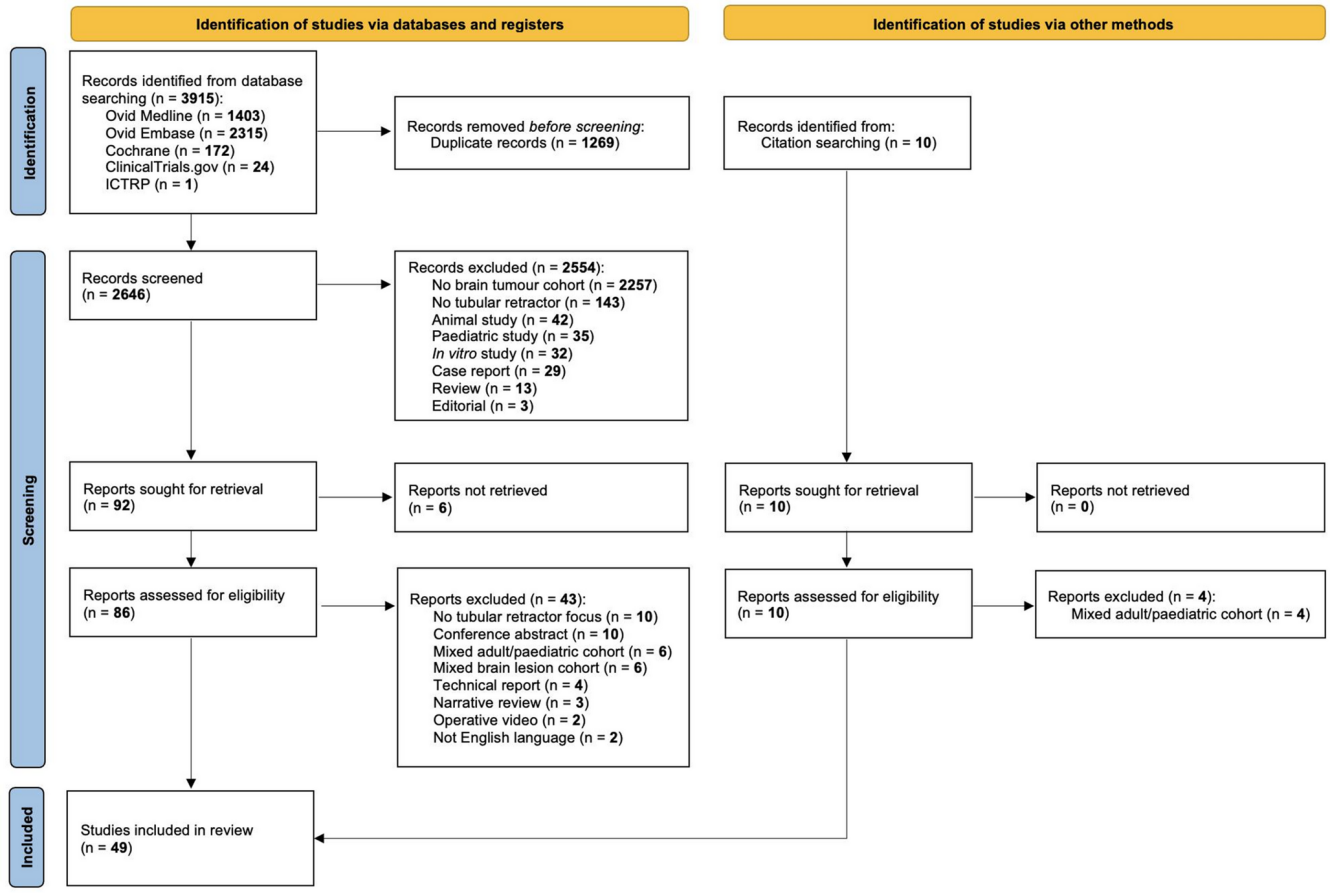
PRISMA flow diagram of study selection

Our search identified 3915 records, of which 2646 were screened following removal of duplicates (Fig. 1). 92 full-text articles were assessed for eligibility, of which 43 were included. An additional six studies were identified by manual search, resulting in 49 studies included in the final analysis [20–68]. In total, 47 studies were case series, one was a cohort study that compared tubular retractor-assisted biopsy to stereotactic biosy [44], and there was one ongoing prospective multicentre randomised controlled trial (RCT) [68]. 

### Study characteristics

Study characteristics of included papers are summarised in Supplementary Data 3. In total, 684 patients were included, with a median study size of 13 (IQR 8–18). Mean age of patients included ranged from 34.9 to 66.8 years, with a median of 51.2 (IQR 45.3–56.1).

The most prevalent tumours by histology were: glioma (*n* = 281; of which 179 were high-grade, 76 were low-grade, and 26 had no grade specified), metastasis (*n* = 156), colloid cyst (*n* = 140), neurocytoma (*n* = 29), and meningioma (*n* = 24). The most prevalent tumours by location were: ventricular (*n* = 247), frontal lobe (*n* = 84), parietal lobe (*n* = 52), thalamus (*n* = 48), and cerebellum (*n* = 40). There were 79 tumours that were multifocal. Nine studies reported on tumour depth, 17 studies reported on diameter, and 12 studies reported on volume.

### Surgical workflow

Characteristics of surgical workflow in each study is summarised in Supplementary Data 4. Three different brands of retractors were commonly used: VBAS (*n* = 214), BrainPath (*n* = 199), and METRx (*n* = 63). Resection using other tubular retractors occurred for 208 cases. Details regarding retractor diameter and length were reported in 31 and 22 studies respectively, with a range of 11–28 mm and 3–9 cm respectively.

A trans-cortical approach was preferred in 18 studies, whilst a trans-sulcal approach was preferred in 19 studies. Seven studies utilised both trans-cortical and trans-sulcal approaches. Four studies did not adequately report on surgical approach [27, 40, 53, 56]. Visualisation was achieved using: microscope (*n* = 15), endoscope (*n* = 15), or exoscope (*n* = 8). Eight studies used some combination of microscope, endoscope, and/or exoscope. Two studies did not adequately report on their visualisation method [62, 65]. The most common surgical adjuncts utilised were: pre-operative diffusion tensor imaging (DTI) (*n* = 21), NICO Myriad (*n* = 10), intra-operative neurophysiological monitoring (IONM) (*n* = 9), intra-operative ultrasound (*n* = 6), and pre-operative functional MRI (fMRI) (*n* = 5). Only eight studies reported on mean operative time, whilst 18 studies reported on length of post-operative hospital stay (Supplementary Data 4).

### Extent of resection

**Fig. 2:**
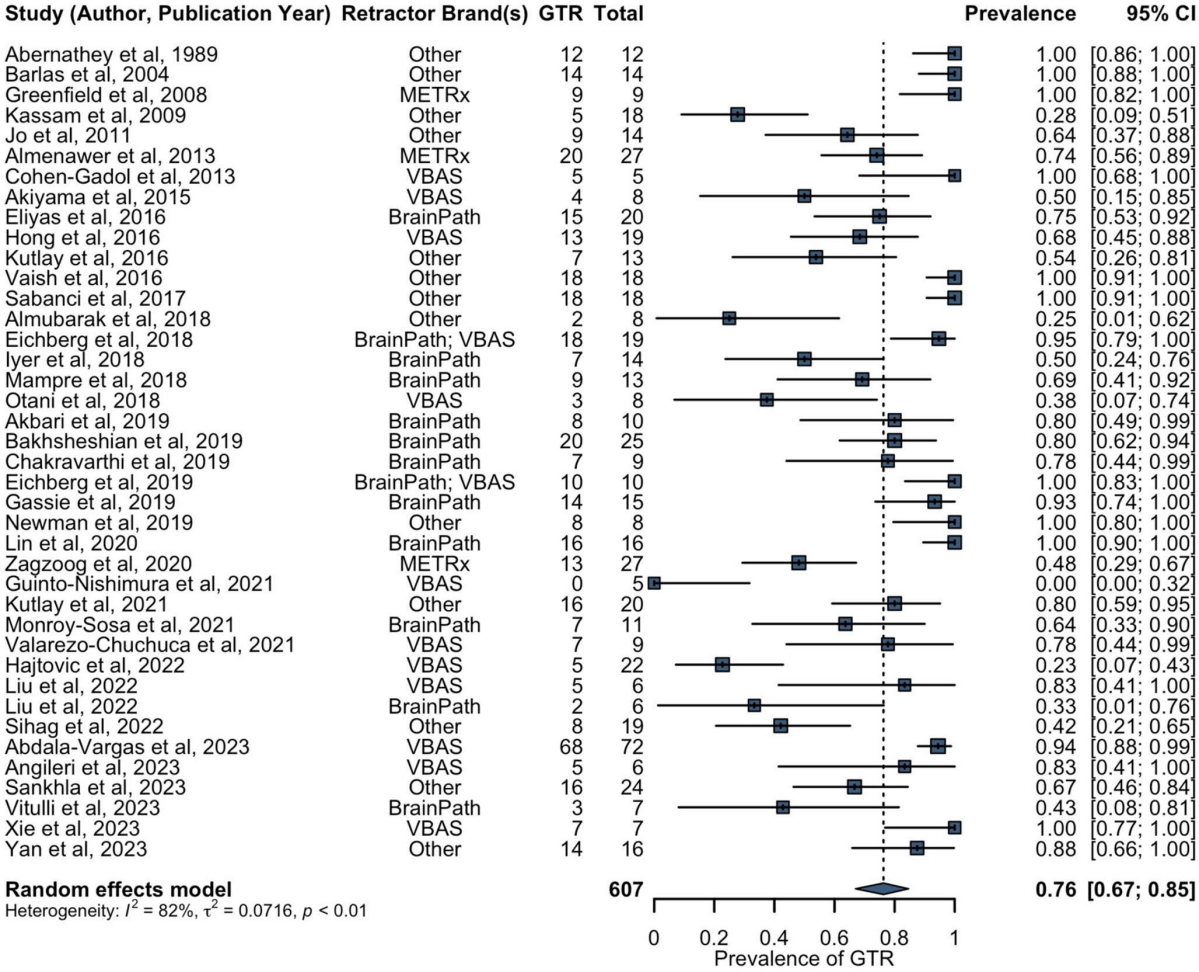
Forest plot of combined prevalence of GTR. GTR = gross total resection; METRx = minimal exposure tubular retractor system; VBAS = viewsite brain access system

All studies, except one [21], reported data on EOR (Supplementary Data 4). GTR was achieved in 458 patients, whilst NTR and STR was achieved in 49 and 119 patients, respectively. Two patients had failed removal using a tubular retractor [26]. Biopsies were performed in 41 cases. The combined pooled prevalence of GTR using tubular retractors was estimated to be 76% (40 studies, 607 patients, 95% CI: 67–85%) (Fig. 2). Leave-one-out sensitivity analysis revealed no influential outliers within the combined pooled prevalence of GTR. Excluding deep-seated thalamic tumours as a sensitivity analysis for surgically-accessible lesions, the combined pooled prevalence of GTR using tubular retractors was estimated to be 80% (38 studies, 557 patients, 95% CI: 71–88%).

**Fig. 3:**
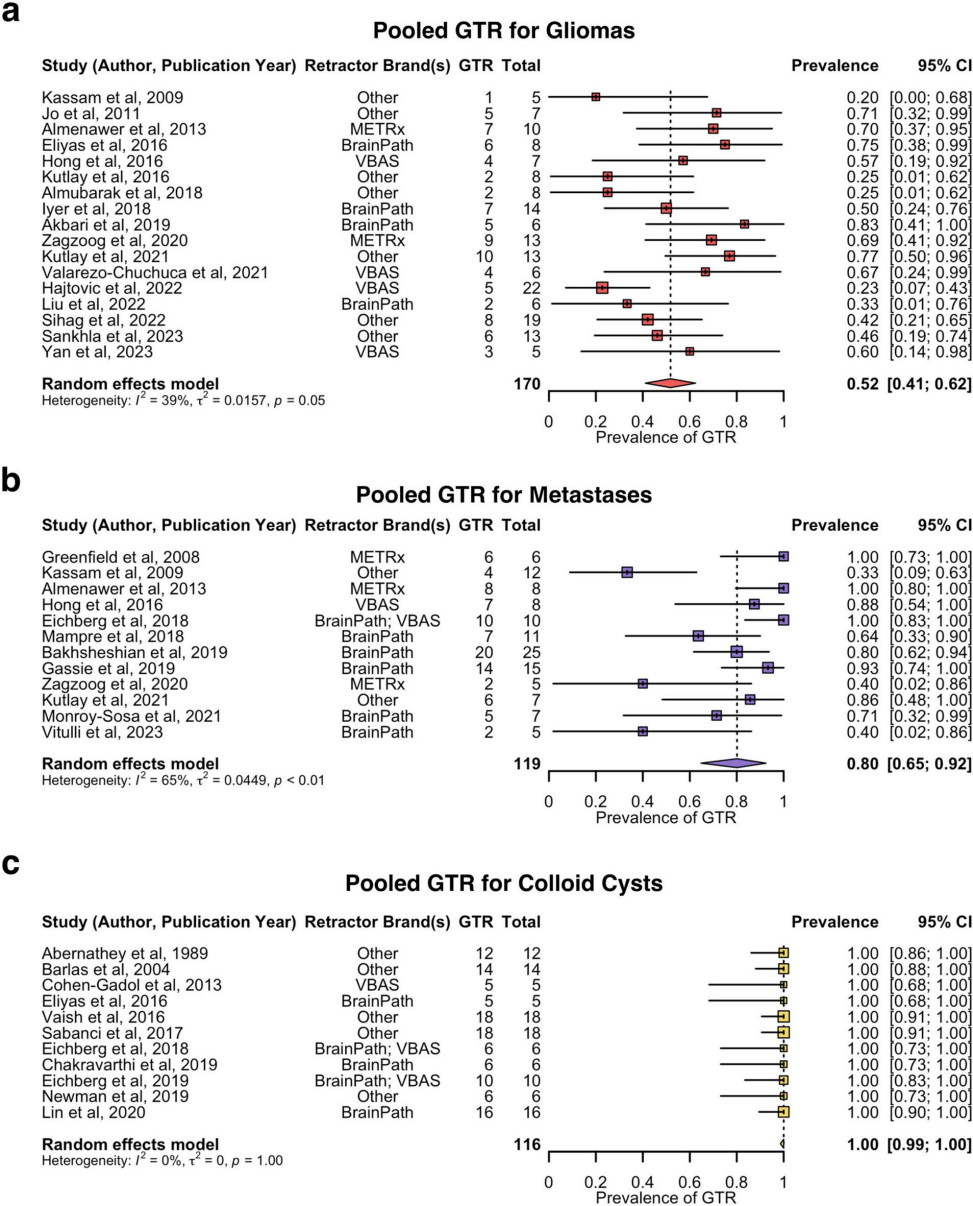
Forest plot of prevalence of GTR by tumour histology. **a**. Pooled prevalence of GTR for gliomas. **b**. Pooled prevenance of GTR for metastases. **c**. Pooled prevalence of GTR for colloid cysts. GTR = gross total resection; METRx = minimal exposure tubular retractor system; VBAS = viewsite brain access system

Subgroup analysis of the pooled prevalence of GTR by tumour histology is shown in Fig. 3. The pooled prevalence of GTR was estimated as follows: 52% for gliomas (17 studies, 170 patients, 95% CI: 41–62%), 80% for metastases (12 studies, 119 patients, 95% CI: 65–92%), and 100% for colloid cysts (11 studies, 116 patients, 95% CI: 99–100%). Sensitivity analysis revealed no influential outliers. The rate of GTR was significantly higher in colloid cysts compared to both metastases (*p* = 1.2 × 10^− 11^) and gliomas (*p* = 2.2 × 10^− 16^), and significantly higher in metastases compared to gliomas (*p* = 2.7 × 10^− 5^) (Supplementary Data 5).

**Fig. 4:**
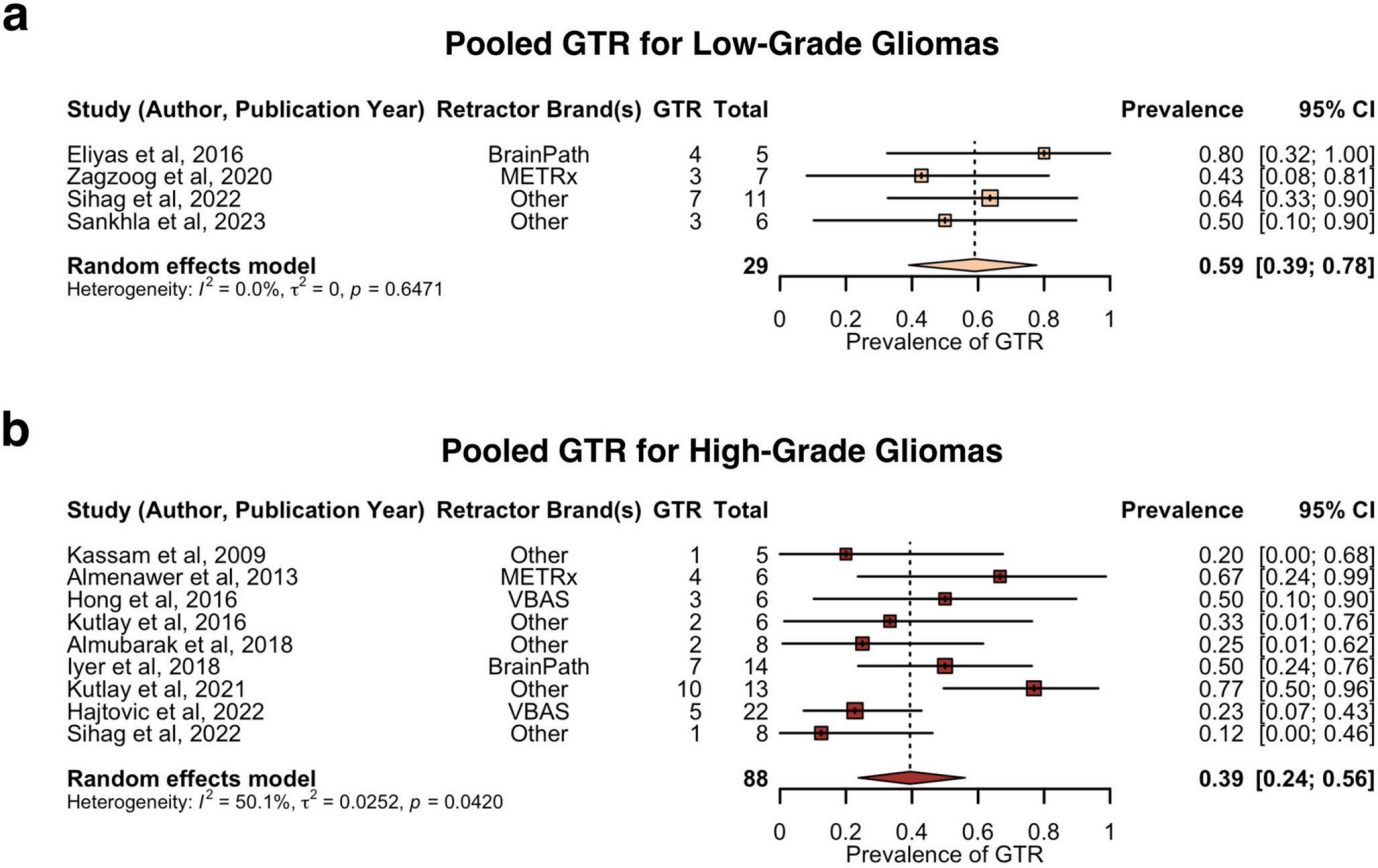
Forest plot of prevalence of GTR in low-grade gliomas versus high-grade gliomas. **a**. Pooled prevalence of GTR for low-grade gliomas. **b**. Pooled prevalence of GTR for high-grade gliomas. GTR = gross total resection; METRx = minimal exposure tubular retractor system; VBAS = viewsite brain access system

Sub-group analysis of the pooled prevalence of GTR comparing low-grade versus high-grade gliomas is shown in Fig. 4. The pooled prevalence of GTR was estimated as follows: 59% for low-grade gliomas (4 studies, 29 patients, 95% CI: 39–78%) and 39% for high-grade gliomas (9 studies, 88 patients, 95% CI: 24–56%). Sensitivity analysis revealed no influential outliers within the low-grade glioma analysis, but indicated Kutlay et al. (2021) as an influential outlier within the high-grade glioma analysis [53]. Excluding for this outlier, estimated pooled prevalence of GTR rate for high-grade gliomas were 32% (8 studies, 75 patients, 95% CI: 21–45%). The rate of GTR was significantly higher in low-grade gliomas compared to high-grade gliomas (*p* = 0.01083) (Supplementary Data 5).

**Fig. 5:**
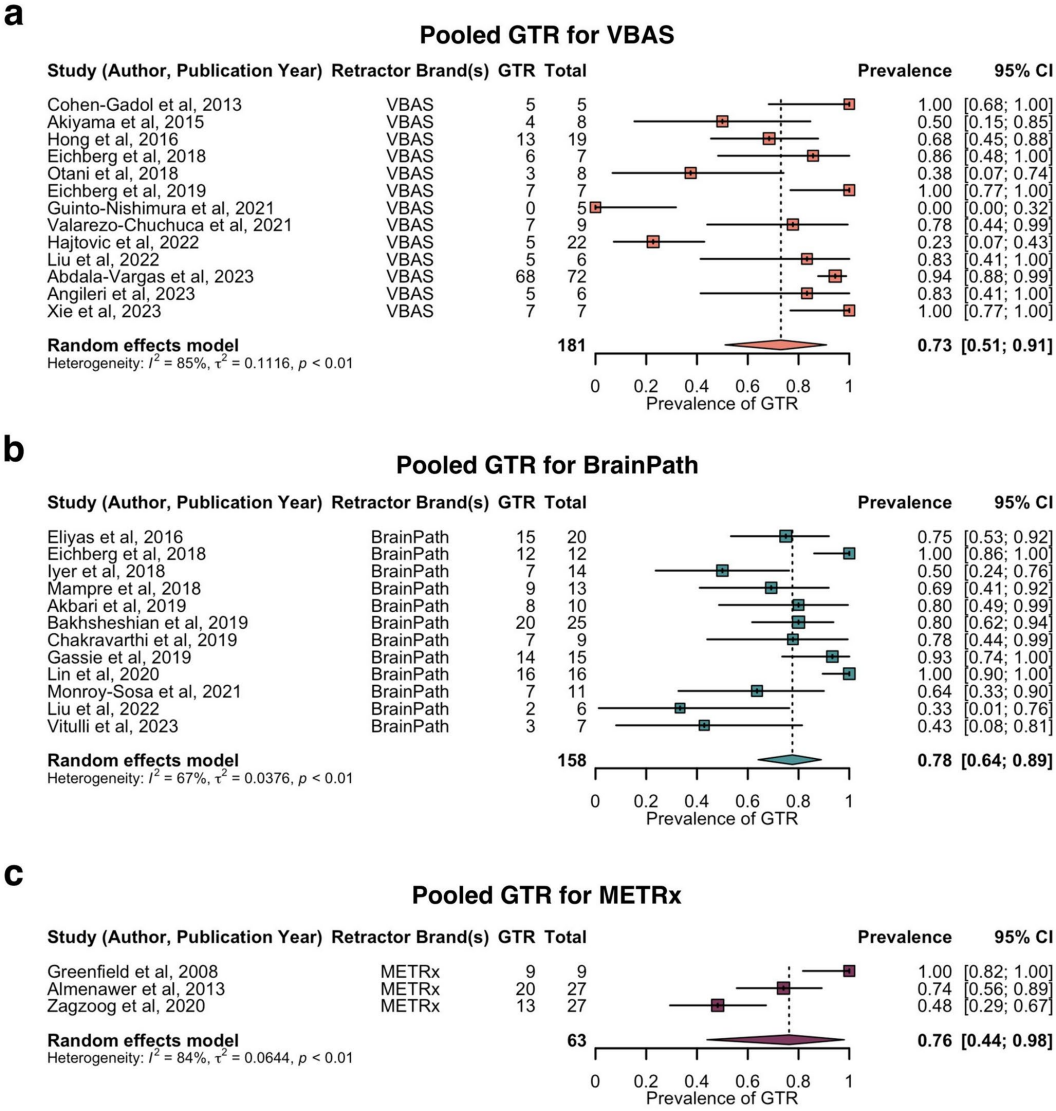
Forest plot of prevalence of GTR by brand of tubular retractor. **a**. Pooled prevalence of GTR for VBAS. **b**. Pooled prevalence of GTR for BrainPath. **c**. Pooled prevalence of GTR for METRx. GTR = gross total resection; METRx = minimal exposure tubular retractor system; VBAS = viewsite brain access system

Subgroup analysis of the pooled prevalence of GTR by brand of retractor is shown in Fig. 5. The pooled prevalence of GTR was estimated as follows: 73% for VBAS (13 studies, 181 patients, 95% CI: 51–91%), 78% for BrainPath (12 studies, 158 patients, 95% CI: 64–89%), and 76% for METRx (three studies, 63 patients, 95% CI: 44–98%). Sensitivity analysis revealed no influential outliers for the VBAS and BrainPath analysis, but indicated Greenfield et al. (2008) and Zagzoog et al. (2020) as influential outliers within the METRx analysis [24, 51], which were not excluded due to the small number of studies in this analysis. There was no significant difference between tubular retractor systems with regards to GTR rate (*p* > 0.05) (Supplementary Data 6).

### Complications

**Fig. 6:**
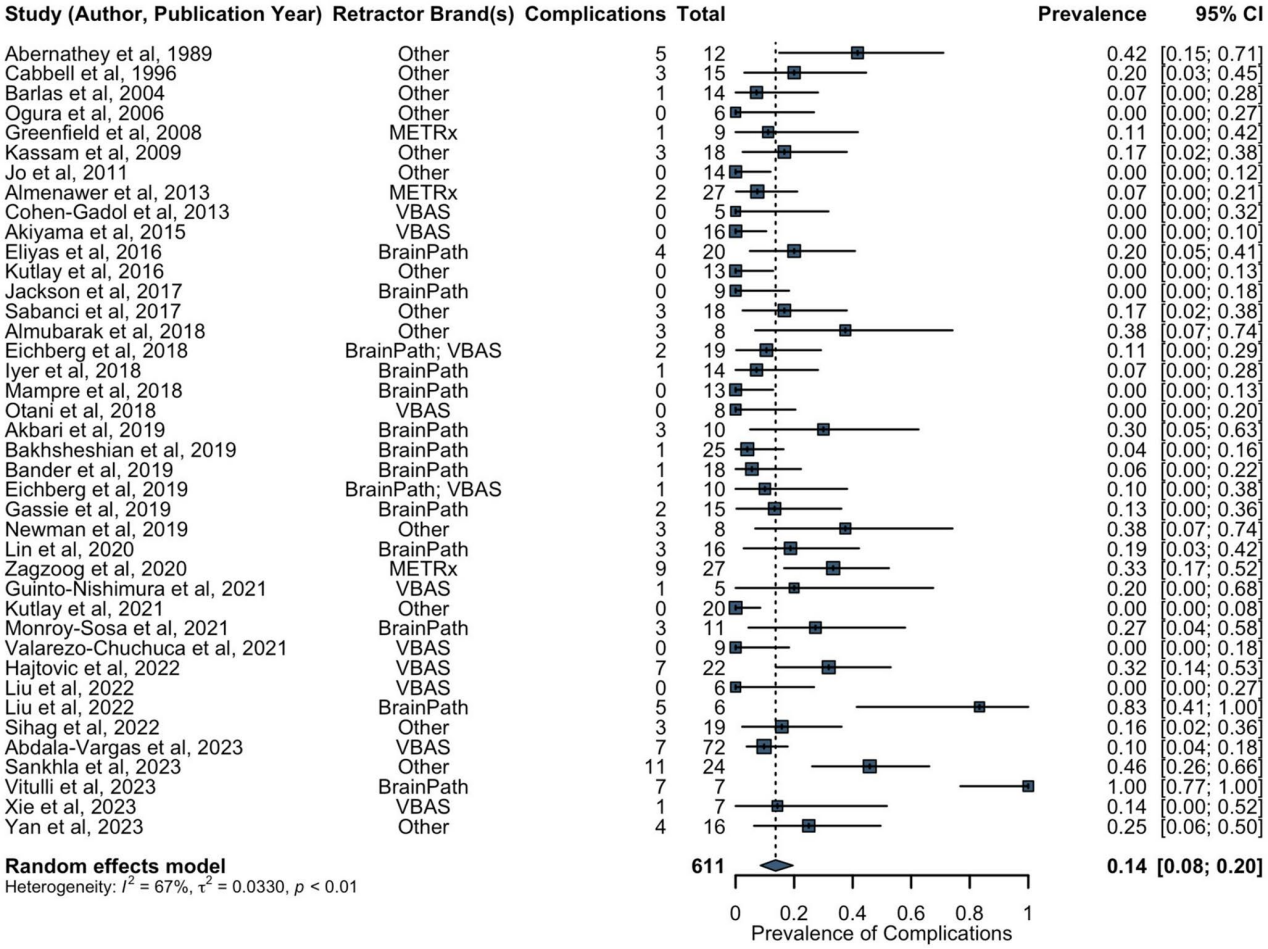
Forest plot of combined prevalence of complications. METRx = minimal exposure tubular retractor system; VBAS = viewsite brain access system

All studies, but three [31, 33, 45], reported on complications after surgery (Supplementary Data 4). In total, there were 109 patients (of 645 with complication data, 16.9%) reported to have complications following use of tubular retractors. The most commonly reported complications were: sensorimotor deficit (31/645, 4.8%), speech and language deficit (16/645, 2.4%), cognitive impairment (13/645, 2.0%), hydrocephalus (13/645, 2.0%), visual deficit (11/645, 1.7%), and surgical site infection (10/645, 1.6%). The rates of focal neurological deficit were 12.4% (28/226) for gliomas, 9.2% (12/130) for metastases, and 6.9% (7/101) for colloid cysts. The rates of hydrocephalus were 2.7% (6/226) for gliomas, 0% (0/130) for metastases, and 3.0% (3/101) for colloid cysts. The rates of surgical site infection were 0.9% (2/226) for gliomas, 0.8% (1/130) for metastases, and 5.0% (5/101) for colloid cysts. The combined pooled prevalence of complications using tubular retractors was estimated to be 14% (40 studies, 611 patients, 95% CI: 8–20%) (Fig. 6). Leave-one-out sensitivity analysis revealed Vitulli et al. (2023) as an influential outlier [65], which when excluded from the analysis results in an estimated combined pooled prevalence of complications of 12% (39 studies, 604 patients, 95% CI: 8–17%).

**Fig. 7:**
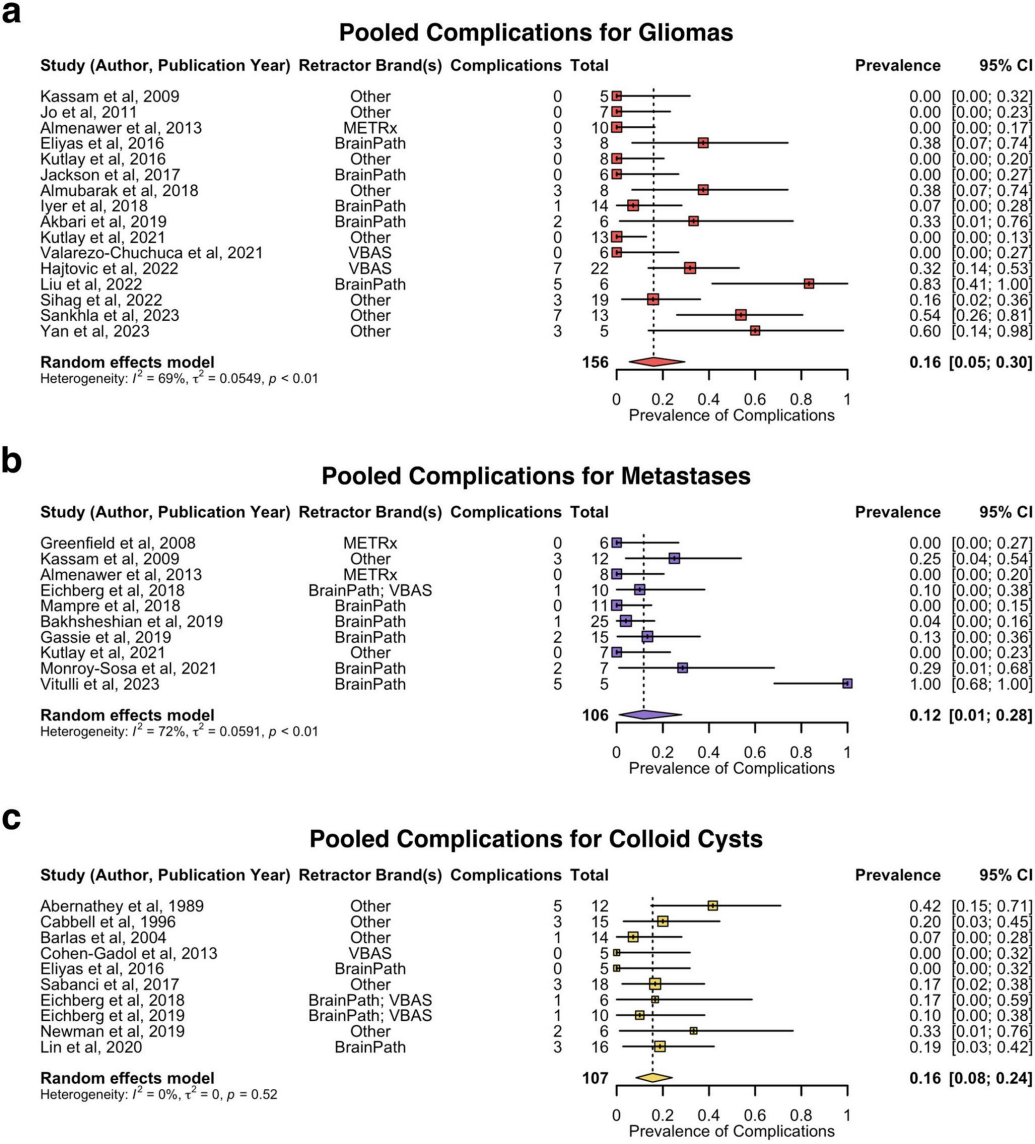
Forest plot of prevalence of complications by tumour histology. **a**. Pooled prevalence of complications for gliomas. **b**. Pooled prevalence of complications for metastases. **c**. Pooled prevalence of complications for colloid cysts. METRx = minimal exposure tubular retractor system; VBAS = viewsite brain access system

Subgroup analysis of the pooled prevalence of complications by tumour histology is shown in Fig. 7. The pooled prevalence of complications was estimated as follows: 16% for gliomas (16 studies, 156 patients, 95% CI: 5–30%), 12% for metastases (10 studies, 106 patients, 95% CI: 1–28%), and 16% for colloid cysts (10 studies, 107 patients, 95% CI: 8–24%). Sensitivity analysis revealed no influential outliers within the gliomas analysis, but indicated Vitulli et al. (2023) and Abernathey et al. (1989) as influential outliers within the metastases and colloid cysts analyses respectively [20, 65]. Excluding for these influential outliers, estimated pooled prevalence of complications for metastases were 6% (9 studies, 101 patients, 95% CI: 1–13%) and for colloid cysts were 13% (9 studies, 95 patients, 95% CI: 6–21%). There was no significant difference in complication rate between colloid cysts and gliomas (*p* > 0.05) or metastases (*p* > 0.05), although the rate of complications was significantly lower in metastases compared to gliomas (*p* = 0.034) (Supplementary Data 7).

**Fig. 8:**
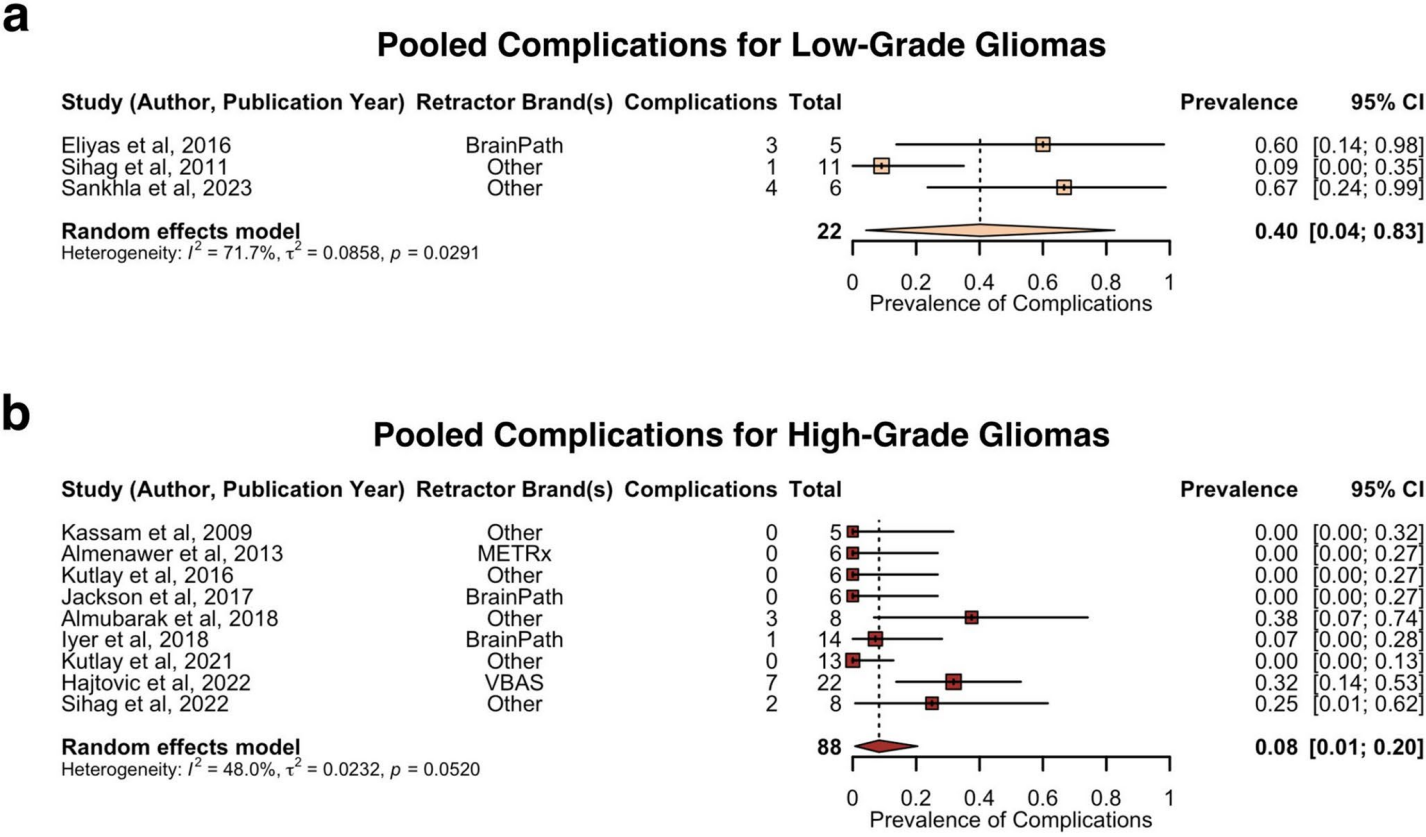
Forest plot of prevalence of complications in low-grade gliomas versus high-grade gliomas. **a**. Pooled prevalence of complications for low-grade gliomas. **b**. Pooled prevalence of complications for high-grade gliomas

Sub-group analysis of the pooled prevalence of complications comparing low-grade versus high-grade gliomas is shown in Fig. 8. The pooled prevalence of complications was estimated as follows: 40% for low-grade gliomas (3 studies, 22 patients, 95% CI: 4–83%) and 8% for high-grade gliomas (9 studies, 88 patients, 95% CI: 1–20%). Sensitivity analysis revealed no influential outliers within the high-grade glioma analysis, but indicated Sihag et al. (2022) as an influential outlier within the low-grade glioma analysis [61]. Due to the small number of studies in the low-grade glioma analysis, no indicated outliers were excluded. There was no significant difference in complication rate between low-grade gliomas compared to high-grade gliomas (*p* = 0.1368) (Supplementary Data 7).

**Fig. 9:**
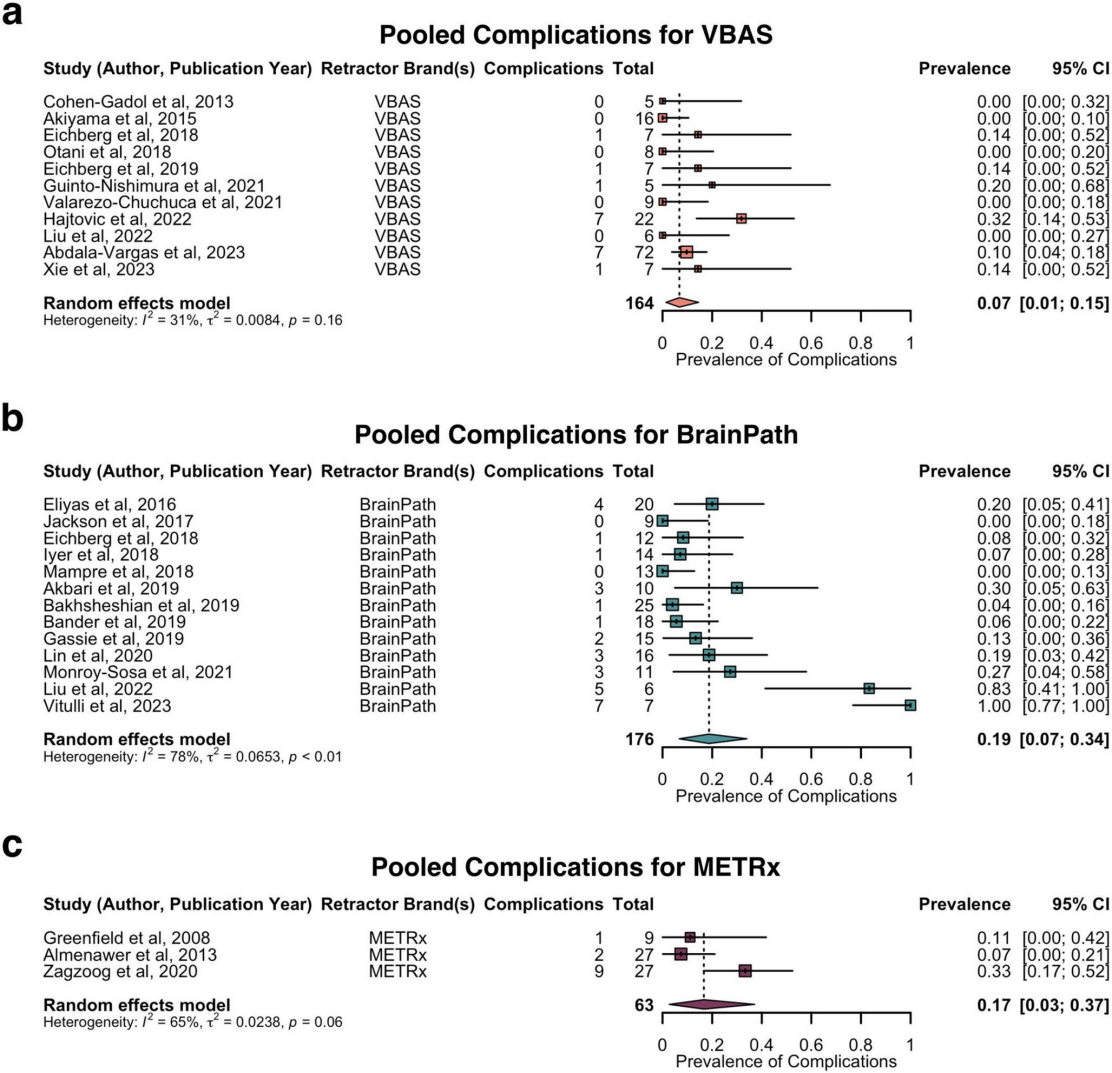
Forest plot of prevalence of complications by brand of tubular retractor. **a.**. Pooled prevalence of complications for VBAS. **b.** Pooled prevalence of complications for BrainPath. **c.** Pooled prevalence of complications for METRx. METRx = minimal exposure tubular retractor system; VBAS = viewsite brain access system

Subgroup analysis of the pooled prevalence of complications by brand of retractor is shown in Fig. 9. The pooled prevalence of complications was estimated as follows: 7% for VBAS (11 studies, 164 patients, 95% CI: 1–15%), 19% for BrainPath (13 studies, 176 patients, 95% CI: 7–34%), and 17% for METRx (three studies, 63 patients, 95% CI: 3–37%). Sensitivity analysis indicated the following studies as influential outliers: Valarezo-Chuchuca et al. (2021) for the VBAS analysis [55], Vitulli et al. (2023) for the BrainPath analysis [65], and Almenawer et al. (2013) and Zagzoog et al. (2020) for the METRx analysis [27, 51]. Excluding for outliers in the VBAS and BrainPath analyses, estimated pooled prevalence of complications were 8% (10 studies, 155 patients, 95% CI: 2–16%) and 13% (12 studies, 169 patients, 95% CI: 5–23%) respectively. Due to the small number of studies in the METRx analysis, no indicated outliers were excluded. There was no significant difference between tubular retractor systems with regards to complication rate (*p* > 0.05) (Supplementary Data 8).

### Assessment of bias and certainty of evidence

With regards to methodological bias, 45 studies were deemed low risk, whilst three studies were deemed to have a moderate risk of bias (Supplementary Data 9) [23, 29, 57]. The lack of clear reporting of patient clinical data, efficacy and safety outcomes, and site(s)/clinic(s) involved in the study were major sources of bias. With regards to publication bias, funnel plots for each forest plot generated are provided in Supplementary Data 10. Certainty in the body of evidence was very low for both efficacy and safety outcomes, largely owing to the predominantly case series design of included studies (Supplementary Data 11).

## Discussion

In this systematic review and meta-analysis, we aimed to take stock of the current literature to assess the utility and scope of tubular retractors in resection of brain tumours. Overall, 49 studies were included with a total of 684 patients, with our results showing that tubular retractors have an encouraging efficacy and safety profile in neuro-oncological surgery. However, the pool of evidence remains largely in the form of case series, which, despite most having a low risk of bias, results in a lack of high certainty in our findings.

The pooled GTR rate for tubular retractors in resection of brain tumours was found to be 76%, building upon findings of a previous meta-analysis of 13 studies, which reported a GTR rate of 75% [12]. The pooled complication rate of tubular retractors was found to be 12–14%, roughly two times greater than that reported in two previous meta-analyses [12, 13]. Differences in patient demographics (such as age and comorbidities), tumour characteristics (particularly histology and location), and length of follow-up may in part account for this difference. Furthermore, how complications are defined within studies, alongside underreporting of complications within the medical literature, are likely other important contributing factors [69]. There is currently a lack of direct comparison between tubular retractors and conventional retractors with regards to efficacy and safety, although our pooled GTR and complication rates for tubular retractors are comparable to that reported for metal blade retractors in the literature [70, 71]. Importantly, whilst previous reviews have estimated the incidence of retraction-induced brain injury to be around 5–10% [8], our estimation for the complication rate of tubular retractors takes into consideration all complications reported, including those that may not be directly related to brain retraction, such as deep vein thrombosis/pulmonary embolism.

Despite the lack of direct comparison to current convention, there is growing pre-clinical and clinical evidence that illustrates the potential role for tubular retractors in neuro-oncological surgery. Firstly, compared with conventional retractors, tubular retractors require a smaller incision, craniotomy, and durotomy, thereby reducing the area of exposed brain. This might reduce risk of surgical site infections [72], consistent with our findings of a surgical site infection rate of 1.6% compared to the 2.2–4.7% reported for traditional craniotomy procedures [73]. Moreover, the atraumatic design of their obturator tip, particularly in VBAS and BrainPath, enables tubular retractors to gradually dilate surrounding healthy brain, which has been theorised to separate white matter bundles instead of dissecting them. More clinical evidence is still required to establish this hypothesis– for example, tractography studies comparing white matter tract integrity before and after tubular retractor-assisted surgery [74]. 

Once inserted through the brain parenchyma, tubular retractors uniformly distribute the pressure required for retraction through their canonical shape, which has been proposed to reduce complications associated with traction, such as post-operative seizures [7]. In support of this, whilst pre-clinical studies using animals have shown that pressures on the brain as low as 25 mmHg can result in electroencephalographic changes with pressures of 30 mmHg reducing cerebral blood flow [75, 76], clinical studies have shown that the pressure from tubular retractors in human patients did not exceed 10 mmHg when used correctly [23]. Furthermore, finite element analyses of brain retraction have predicted that tubular retractors result in lower stresses than conventional retraction [77], whilst quantitative MRI analyses have shown that tubular retractors do not cause significant FLAIR signals in human patients [78], supporting the notion that tubular retractors can minimise retraction-induced brain injury. Despite these findings, several studies have reported diffusion-tensor intensities around areas of tubular retraction, indicating that tubular retractors may result in some level of cytotoxic oedema and cellular damage to the brain [78–80]. Importantly, retraction-induced brain injury is likely a function of both magnitude and duration of retraction [81], so studies should establish whether there are differences in length of time required for each type of retraction.

Whilst we found that the rates of GTR and complications did not differ significantly between brands of tubular retractor, there are significant differences when considering tumour histology. The core designs of tubular retractors have several similarities, rendering it unlikely to identify statistically significant differences in efficacy or safety at case series level (although not excluding an individual surgeon preference). Meanwhile, differences in rates of GTR and complications for tubular retractors dependent on tumour histology is consistent with the literature for conventional retraction, where diverse rates of GTR are similarly reported [82, 83]. Considering tumour histology directly influences tumour physical characteristics, this finding is not surprising. For example, whilst brain metastases are typically well-demarcated tumours, high-grade gliomas are often poorly-defined with oedematous infiltration rendering them often more surgically challenging [84]. As such, future studies should control for tumour histology, location, and size as likely cofounders, through stringent selection criteria or in subsequent multivariate regression analyses.

Notably, tubular retractors are increasingly combined with other adjuncts, including pre-operative DTI and fMRI and intra-operative ultrasound and neurophysiological monitoring. With the advent of large multicentred RCTs, such as FUTURE-GB [85], the benefits conferred by each individual tool can be assessed. However, it is likely that adjuncts work synergistically together, so smaller exploratory studies are still beneficial to optimise surgical workflows [65]. There is on-going debate regarding the advantages of trans-cortical or trans-sulcal approaches in neuro-oncological surgery [86], particularly with the emergence of MIPS [14]. In reality, there is likely no universally optimised approach, with the surgeon’s experience and the individual needs of each patient’s unique case guiding the choice of surgical approach and adjuncts.

Ultimately, whilst pre-clinical and early clinical data provide a proof-of-concept for the utility of tubular retractors in neuro-oncological surgery, the crucial next step will be direct comparison against conventional retraction. Most studies included in our review were retrospective case series with small sample sizes, thus limiting certainty in our findings. Prospective cohort, non-randomised, or randomised controlled studies with larger cohort sizes and that directly compare tubular retractors against conventional retraction are now needed to robustly establish the role of tubular retractors in neuro-oncological surgery and to enable evidence-based decisions when planning procedures [87]. RCTs will be especially beneficial to limit potential selection bias, which may be present in our included studies, whilst additionally determining metrics beyond efficacy and safety, such as cost-effectiveness and clinical feasibility of tubular retractors that were unable to be evaluated in this review [88]. Encouragingly, there is an on-going prospective multicentre RCT investigating the utility of tubular retractors in intracranial surgery [68]. Furthermore, whilst our review focusses on neuro-oncological surgery, tubular retractors have also been assessed in the subfield of vascular neurosurgery, particularly the ENRICH trial for the management of intracerebral haemorrhages [14], further supporting the potential role of tubular retractors as a useful adjunct in the management of neurosurgical patients.

Other limitations of our review include the significant heterogeneity across studies included in our meta-analysis as indicated by the I^2^ characteristics > 50% for most analyses. Given the small sample size of most papers, we aimed to include all studies in our analysis to best estimate rate of GTR and complications. We mitigated some heterogeneity by running random effects models and by performing sub-group analysis for specific tumour histology and brand of tubular retractor. Encouragingly, we did not identify significant publication bias for most analyses, except in three cases, of which two were likely in part due to the analyses including only three studies. The heterogeneity also extends to completeness of data reporting across included studies. The most effective papers reported all of tumour histology, location, and dimensional characteristics. Most studies reported EOR in terms of categorical variables based on GTR/NTR/STR, which precluded a meta-analysis beyond an assessment of prevalence of GTR. Reporting of exact numerical EOR data in future studies would enable a more comprehensive analysis of volumetric resection estimates, and hence tubular retractor efficacy. Moreover, EOR may not always be an appropriate measure of tubular retractor efficacy, particularly in cases where planned EOR is subtotal, so future studies would benefit from clearly reporting their pre-operative EOR goal. Finally, the lack of reporting extends to survival outcomes and length of follow-up, which further limits the findings of this review, with the latter potentially resulting in failure to capture the complete extent of less common complications, such as seizures. Future studies with more extensive reporting beyond EOR and complications, such as overall survival and quality of life, and longer follow-up will therefore be greatly beneficial in validating the long-term benefits of tubular retractors in neuro-oncological surgery.

## Conclusion

In conclusion, the literature provides mounting interest regarding the utility of tubular retractors in neuro-oncological surgery for select cases. Despite the promising efficacy and safety profiles of tubular retractors, with an overall pooled prevalence of 76% for GTR and 14% for complications, providing a proof-of-concept for their utility in neuro-oncological surgery, the certainty in the body of evidence remains limited, largely due to the predominately case series evidence base. Extent of resection using tubular retractors differed significantly between different types of tumour histology, with a GTR rate of 52% for gliomas, 80% for metastases, and 100% for colloid cysts, but to a lesser extent for complications, with a rate of 16% for gliomas, 12% for metastases, and 16% for colloid cysts. We found no significant difference in terms of both efficacy and safety between different brands of tubular retractors. Studies with larger cohort sizes, longer follow-up, and controlled comparison with conventional retraction are now needed to more robustly establish the position of tubular retractors in neuro-oncological surgery.

## Electronic supplementary material

Below is the link to the electronic supplementary material.

Supplementary Material 1

## Data Availability

The data collected for this study can be provided upon reasonable request.
