# Supplementary material for: Tubular retractors in neuro-oncological surgery: a systematic review and meta-analysis
Source: Neurosurg Rev. 2025 Jun 27;48(1):530. doi: 10.1007/s10143-025-03677-w (PMC12204906; doi:10.1007/s10143-025-03677-w)
Supplement: Supplementary file 1 — Supplementary Material 1 [file 10143_2025_3677_MOESM1_ESM.docx]

**Supplementary Data 1 – PRISMA Checklist**

| **Section and Topic** | **Item #** | **Checklist item** | **Location where item is reported** |
| --- | --- | --- | --- |
| **TITLE** | | |  |
| Title | 1 | Identify the report as a systematic review. | Title – Pg 1 |
| **ABSTRACT** | | |  |
| Abstract | 2 | See the PRISMA 2020 for Abstracts checklist. | Abstract – Pg 2,3 |
| **INTRODUCTION** | | |  |
| Rationale | 3 | Describe the rationale for the review in the context of existing knowledge. | Introduction – Pg 4-5 |
| Objectives | 4 | Provide an explicit statement of the objective(s) or question(s) the review addresses. | Introduction – Pg 5 |
| **METHODS** | | |  |
| Eligibility criteria | 5 | Specify the inclusion and exclusion criteria for the review and how studies were grouped for the syntheses. | Methods – Pg 6 |
| Information sources | 6 | Specify all databases, registers, websites, organisations, reference lists and other sources searched or consulted to identify studies. Specify the date when each source was last searched or consulted. | Methods – Pg 6 |
| Search strategy | 7 | Present the full search strategies for all databases, registers and websites, including any filters and limits used. | Methods – Pg 6  Supplementary Data 2 |
| Selection process | 8 | Specify the methods used to decide whether a study met the inclusion criteria of the review, including how many reviewers screened each record and each report retrieved, whether they worked independently, and if applicable, details of automation tools used in the process. | Methods – Pg 6 |
| Data collection process | 9 | Specify the methods used to collect data from reports, including how many reviewers collected data from each report, whether they worked independently, any processes for obtaining or confirming data from study investigators, and if applicable, details of automation tools used in the process. | Methods – Pg 6,7 |
| Data items | 10a | List and define all outcomes for which data were sought. Specify whether all results that were compatible with each outcome domain in each study were sought (e.g. for all measures, time points, analyses), and if not, the methods used to decide which results to collect. | Methods – Pg 6,7 |
|  | 10b | List and define all other variables for which data were sought (e.g. participant and intervention characteristics, funding sources). Describe any assumptions made about any missing or unclear information. | Methods – Pg 6,7 |
| Study risk of bias assessment | 11 | Specify the methods used to assess risk of bias in the included studies, including details of the tool(s) used, how many reviewers assessed each study and whether they worked independently, and if applicable, details of automation tools used in the process. | Methods – Pg 7 |
| Effect measures | 12 | Specify for each outcome the effect measure(s) (e.g. risk ratio, mean difference) used in the synthesis or presentation of results. | Methods – Pg 7,8 |
| Synthesis methods | 13a | Describe the processes used to decide which studies were eligible for each synthesis (e.g. tabulating the study intervention characteristics and comparing against the planned groups for each synthesis (item #5)). | Methods – Pg 7,8 |
|  | 13b | Describe any methods required to prepare the data for presentation or synthesis, such as handling of missing summary statistics, or data conversions. | Methods – Pg 7,8 |
|  | 13c | Describe any methods used to tabulate or visually display results of individual studies and syntheses. | Methods – Pg 7,8 |
|  | 13d | Describe any methods used to synthesize results and provide a rationale for the choice(s). If meta-analysis was performed, describe the model(s), method(s) to identify the presence and extent of statistical heterogeneity, and software package(s) used. | Methods – Pg 7,8 |
|  | 13e | Describe any methods used to explore possible causes of heterogeneity among study results (e.g. subgroup analysis, meta-regression). | Methods – Pg 7,8 |
|  | 13f | Describe any sensitivity analyses conducted to assess robustness of the synthesized results. | Methods – Pg 8 |
| Reporting bias assessment | 14 | Describe any methods used to assess risk of bias due to missing results in a synthesis (arising from reporting biases). | Methods – Pg 8 |
| Certainty assessment | 15 | Describe any methods used to assess certainty (or confidence) in the body of evidence for an outcome. | Methods – Pg 7 |
| **RESULTS** | | |  |
| Study selection | 16a | Describe the results of the search and selection process, from the number of records identified in the search to the number of studies included in the review, ideally using a flow diagram. | Results – Pg 9 |
|  | 16b | Cite studies that might appear to meet the inclusion criteria, but which were excluded, and explain why they were excluded. | Results – Pg 9  Figure 1 |
| Study characteristics | 17 | Cite each included study and present its characteristics. | Results – Pg 9  Supplementary Data 3 |
| Risk of bias in studies | 18 | Present assessments of risk of bias for each included study. | Results – Pg 12  Supplementary Data 9 |
| Results of individual studies | 19 | For all outcomes, present, for each study: (a) summary statistics for each group (where appropriate) and (b) an effect estimate and its precision (e.g. confidence/credible interval), ideally using structured tables or plots. | Results – Pg 10-12  Figures 2-7  Supplementary Data 4 |
| Results of syntheses | 20a | For each synthesis, briefly summarise the characteristics and risk of bias among contributing studies. | Results – Pg 10-12  Supplementary Data 4,9 |
|  | 20b | Present results of all statistical syntheses conducted. If meta-analysis was done, present for each the summary estimate and its precision (e.g. confidence/credible interval) and measures of statistical heterogeneity. If comparing groups, describe the direction of the effect. | Results – Pg 10-12  Figures 2-7 |
|  | 20c | Present results of all investigations of possible causes of heterogeneity among study results. | Results – Pg 10-12  Figures 3,4,6,7 |
|  | 20d | Present results of all sensitivity analyses conducted to assess the robustness of the synthesized results. | Results – Pg 10-12 |
| Reporting biases | 21 | Present assessments of risk of bias due to missing results (arising from reporting biases) for each synthesis assessed. | Results – Pg 12  Supplementary Data 10 |
| Certainty of evidence | 22 | Present assessments of certainty (or confidence) in the body of evidence for each outcome assessed. | Results – Pg 12  Supplementary Data 11 |
| **DISCUSSION** | | |  |
| Discussion | 23a | Provide a general interpretation of the results in the context of other evidence. | Discussion – Pg 13 |
|  | 23b | Discuss any limitations of the evidence included in the review. | Discussion – Pg 16,17 |
|  | 23c | Discuss any limitations of the review processes used. | Discussion – Pg 16,17 |
|  | 23d | Discuss implications of the results for practice, policy, and future research. | Discussion – Pg 13-17 |
| **OTHER INFORMATION** | | |  |
| Registration and protocol | 24a | Provide registration information for the review, including register name and registration number, or state that the review was not registered. | Abstract – Pg 2  Methods – Pg 6 |
|  | 24b | Indicate where the review protocol can be accessed, or state that a protocol was not prepared. | Abstract – Pg 2  Methods – Pg 6 |
|  | 24c | Describe and explain any amendments to information provided at registration or in the protocol. | N/A |
| Support | 25 | Describe sources of financial or non-financial support for the review, and the role of the funders or sponsors in the review. | Pg 19 |
| Competing interests | 26 | Declare any competing interests of review authors. | Pg 19 |
| Availability of data, code and other materials | 27 | Report which of the following are publicly available and where they can be found: template data collection forms; data extracted from included studies; data used for all analyses; analytic code; any other materials used in the review. | Pg 19 |

**Supplementary Data 2 – Search Strategy**

Full search phrases used for Medline, Embase and Cochrane on 14^th^ July 2024

| **Ovid Medline** | | 1403 articles |
| --- | --- | --- |
| Brain Tumour concept | | |
| 1 | exp brain neoplasms/ | |
| 2 | ((brain or cerebral) adj3 (neoplasm* or tum?r* or cancer* or lesion* or disease* or malignancy or metasta*)).ti,ab,kf,kw. | |
| 3 | (neurooncology or neuro-oncology).ti,ab,kf,kw. | |
| 4 | (deep adj3 lesion* or deep-seated or deep seated).ti,ab,kf,kw. | |
| 5 | exp basal ganglia/ or exp thalamus/ or exp pituitary gland/ or exp pineal gland/ or (basal gangli* or thalam* or pituitary or hypophy* or insula* or pineal or eloquent).ti,ab,kf,kw. | |
| 6 | exp cerebellum/ or exp middle cerebellar peduncle/ or exp cranial fossa, posterior/ or (cerebell* or vermis or peduncle or posterior fossa).ti,ab,kf,kw. | |
| 7 | exp brain ventricle/ or exp choroid/ or (ventric* or choroid).ti,ab,kf,kw. | |
| 8 | exp glioma/ or (glioma* or astrocytoma* or glioblastoma* or GBM or ependymoma*).ti,ab,kf,kw. | |
| 9 | exp meningioma/ or meningioma*.ti,ab,kf,kw. | |
| 10 | exp neuroectodermal tumors/ or (neuroectodermal or neurocytoma* or craniopharyngioma*).ti,ab,kf,kw. | |
| 11 | exp colloid cysts/ or (colloid cyst* or cyst*).ti,ab,kf,kw. | |
| 12 | 1 or 2 or 3 or 4 or 5 or 6 or 7 or 8 or 9 or 10 or 11 | |
| Tubular Retractor concept | | |
| 13 | ((tubular or tube or port) adj3 (retract*)).ti,ab,kf,kw. | |
| 14 | endoport.ti,ab,kf,kw. | |
| 15 | ((minimal* invasi* adj3 tubular retract*) or MITR or (minimal* invasi* adj3 parafasicular) or MIPS or (tubular adj3 minimal* invasi*)).ti,ab,kf,kw. | |
| 16 | (METRx or Vycor or ViewSite Brain Access System or VBAS or BrainPath or brain path or Myriad).ti,ab,kf,kw. | |
| 17 | (cylindrical retractor).ti,ab,kf,kw. | |
| 18 | 13 or 14 or 15 or 16 or 17 | |
| Combined concepts | | |
| 19 | 12 and 18 | |

| **Ovid Embase** | | 2315 articles |
| --- | --- | --- |
| Brain Tumour concept | | |
| 1 | exp brain tumor/ | |
| 2 | ((brain or cerebral) adj3 (neoplasm* or tum?r* or cancer* or lesion* or disease* or malignancy or metasta*)).ti,ab,kf,kw. | |
| 3 | (neurooncology or neuro-oncology).ti,ab,kf,kw. | |
| 4 | (deep adj3 lesion* or deep-seated or deep seated).ti,ab,kf,kw. | |
| 5 | exp basal ganglion/ or exp hypophysis/ or exp thalamus/ or exp pineal body/ or (basal gangli* or thalam* or pituitary or hypophy* or insula* or pineal or eloquent).ti,ab,kf,kw. | |
| 6 | exp cerebellum vermis/ or exp posterior fossa/ or (cerebell* or vermis or peduncle or posterior fossa).ti,ab,kf,kw. | |
| 7 | exp brain ventricle/ or exp choroid/ or (ventric* or choroid).ti,ab,kf,kw. | |
| 8 | exp glioma/ or (glioma* or astrocytoma* or glioblastoma* or GBM or ependymoma*).ti,ab,kf,kw. | |
| 9 | exp meningioma/ or meningioma*.ti,ab,kf,kw. | |
| 10 | exp neuroectoderm tumor/ or exp neuroepithelioma/ or (neuroectodermal or neurocytoma* or craniopharyngioma*).ti,ab,kf,kw. | |
| 11 | exp colloid cyst/ or (colloid cyst* or cyst*).ti,ab,kf,kw. | |
| 12 | 1 or 2 or 3 or 4 or 5 or 6 or 7 or 8 or 9 or 10 or 11 | |
| Tubular Retractor concept | | |
| 13 | exp tubular retractor/ | |
| 14 | ((tubular or tube or port) adj3 (retract*)).ti,ab,kf,kw. | |
| 15 | endoport.ti,ab,kf,kw. | |
| 16 | ((minimal* invasi* adj3 tubular retract*) or MITR or (minimal* invasi* adj3 parafasicular) or MIPS or (tubular adj3 minimal* invasi*)).ti,ab,kf,kw. | |
| 17 | (METRx or Vycor or ViewSite Brain Access System or VBAS or BrainPath or brain path or Myriad).ti,ab,kf,kw. | |
| 18 | (cylindrical retractor).ti,ab,kf,kw. | |
| 19 | 13 or 14 or 15 or 16 or 17 or 18 | |
| Combined concepts | | |
| 20 | 12 and 19 | |

| **Cochrane** | | 172 articles |
| --- | --- | --- |
| Brain Tumour concept | | |
| 1 | MeSH descriptor: [Brain Neoplasms] explode all trees | |
| 2 | ((brain or cerebral) near/3 (neoplasm* or tum?r* or cancer* or lesion* or disease* or malignancy or metasta*)):ti,ab,kw (Word variations have been searched) | |
| 3 | (neurooncology or neuro-oncology):ti,ab,kw (Word variations have been searched | |
| 4 | (deep near/3 lesion* or deep-seated or deep seated):ti,ab,kw (Word variations have been searched) | |
| 5 | MeSH descriptor: [Basal Ganglia] explode all trees | |
| 6 | MeSH descriptor: [Thalamus] explode all trees | |
| 7 | MeSH descriptor: [Pituitary Gland] explode all trees | |
| 8 | MeSH descriptor: [Pineal Gland] explode all trees | |
| 9 | (basal gangli* or thalam* or pituitary or hypophy* or insula* or pineal or eloquent):ti,ab,kw (Word variations have been searched) | |
| 10 | MeSH descriptor: [Cerebellum] explode all trees | |
| 11 | MeSH descriptor: [Middle Cerebellar Peduncle] explode all trees | |
| 12 | MeSH descriptor: [Cranial Fossa, Posterior] explode all trees | |
| 13 | (cerebell* or vermis or peduncle or posterior fossa):ti,ab,kw (Word variations have been searched) | |
| 14 | MeSH descriptor: [Cerebral Ventricles] explode all trees | |
| 15 | (ventric* or choroid):ti,ab,kw (Word variations have been searched) | |
| 16 | MeSH descriptor: [Glioma] explode all trees | |
| 17 | (glioma* or astrocytoma* or glioblastoma* or GBM or ependymoma*):ti,ab,kw (Word variations have been searched) | |
| 18 | MeSH descriptor: [Meningioma] explode all trees | |
| 19 | (meningioma*):ti,ab,kw (Word variations have been searched) | |
| 20 | MeSH descriptor: [Neuroectodermal Tumors] explode all trees | |
| 21 | (neuroectodermal or neurocytoma* or craniopharyngioma*):ti,ab,kw (Word variations have been searched) | |
| 22 | MeSH descriptor: [Colloid Cysts] explode all trees | |
| 23 | (colloid cyst* or cyst*):ti,ab,kw (Word variations have been searched) | |
| 24 | #1 or #2 or #3 or #4 or #5 or #6 or #7 or #8 or #9 or #10 or #11 or #12 or #13 or #14 or #15 or #16 or #17 or #18 or #19 or #20 or #21 or #22 or #23 | |
| Tubular Retractor concept | | |
| 25 | ((tubular or tube or port) near/3 (retract*)):ti,ab,kw (Word variations have been searched) | |
| 26 | (endoport):ti,ab,kw (Word variations have been searched) | |
| 27 | ((minimal* invasi* adj3 tubular retract*) or MITR or (minimal* invasi* adj3 parafasicular) or MIPS or (tubular adj3 minimal* invasi*)):ti,ab,kw (Word variations have been searched) | |
| 28 | (METRx or Vycor or ViewSite Brain Access System or VBAS or BrainPath or brain path or Myriad):ti,ab,kw (Word variations have been searched) | |
| 29 | (cylindrical retractor):ti,ab,kw (Word variations have been searched) | |
| 30 | #25 or #26 or #27 or #28 | |
| Combined concepts | | |
| 31 | #24 and #30 | |

Search phrases used for ClinicalTrials.gov and ICTRP on 14^th^ July 2024

| **Search Terms** | **ClinicalTrials.gov** | **ICTRP** |
| --- | --- | --- |
| 1. “brain neoplasm*” and “tubular retract*” | 0 | 0 |
| 2. “brain neoplasm*” and “minimally invasive” | 8 | 0 |
| 3. “brain tumor*” and “tubular retract*” | 0 | 1 |
| 4. “brain tumor*” and “minimally invasive” | 8 | 0 |
| 5. “brain tumour*” and “tubular retract*” | 0 | 0 |
| 6. “brain tumour*” and “minimally invasive” | 8 | 0 |

**Supplementary Data 3 – Study Characteristics**

**Summary of study characteristics of included studies**.

*DNT = dysembryoplastic neuroepithelial tumour; HGG = high-grade glioma; LGG = low-grade glioma; METRx = minimal exposure tubular retractor system; NR = not reported; PNT = primitive neuroectodermal tumour; VBAS = viewsite brain access system.*

| **Reference** | **Retractor Brand(s)** | **Sample Size (M/F)** | **Mean Age (Range)** | **Tumour Histology (n)** | **Tumour Location (n)** | **Mean Tumour Depth (Range)** | **Mean Tumour Diameter (Range)** | **Mean Tumour Volume (Range)** |
| --- | --- | --- | --- | --- | --- | --- | --- | --- |
| Abernathey et al, 1989 | Other (Cylindrical Retractor) | 12 (6/6) | 51.0 (25-71) | • colloid cyst (12) | • third ventricle (12) | NR | NR | NR |
| Cabbell et al, 1996 | Other (Cylindrical Retractor) | 15 (7/8) | 44.8 (30-58) | • colloid cyst (15) | • third ventricle (15) | NR | NR | NR |
| Barlas et al, 2004 | Other (Cylindrical Retractor) | 14 (9/5) | 39.0 (22-62) | • colloid cyst (14) | • third ventricle (14) | NR | 17.9mm (8-23mm) | NR |
| Ogura et al, 2006 | Other (Transparent Plastic Tube) | 6 (4/2) | 55.6 (18-76) | • HGG (3)  • metastasis (2)  • primary CNS lymphoma (1) | • frontal (4)  • intraventricular thalamic (2) | NR | NR | NR |
| Greenfield et al, 2008 | METRx | 9 (3/6) | 66.1 (48-81) | • HGG (2)  • meningioma (1)  • metastasis (6) | • frontal (1)  • parietal (3)  • temporal (3)  • occipital (1)  • lateral ventricle (1) | >20mm (no mean or range data) | NR | NR |
| Kassam et al, 2009 | Other (Omni Transparent Conduit) | 18 (9/9) | 59.7 (31-79) | • hemangioblastoma (1)  • HGG (5)  • metastasis (12) | • frontal (2)  • parietal (1)  • temporal (1)  • cerebellum (7)  • multifocal (7) | 13.4mm (1-26mm) | 40.6mm (18-64mm) | NR |
| Jo et al, 2011 | Other (Modified Tubular Conduit) | 14 (11/3) | 40.8 (19-68) | • colloid cyst (1)  • HGG (4)  • LGG (3)  • meningioma (2)  • metastasis (2)  • neurocytoma (1)  • pituitary adenoma (1) | • frontal (2)  • parietal (1)  • lateral ventricle (6)  • third ventricle (2)  • basal ganglia (2)  • thalamus (1) | NR | 35.0mm (10-60mm) | NR |
| Almenawer et al, 2013 | METRx | 27 (14/13) | 51.7 (18-77) | • colloid cyst (1)  • craniopharyngioma (1)  • epidermoid cyst (2)  • HGG (6)  • LGG (3)  • LGG or HGG (1)  • meningioma (1)  • metastasis (8)  • neurocytoma (4) | • frontal (7)  • parietal (5)  • temporal (5)  • lateral ventricle (5)  • third ventricle (1)  • fourth ventricle (1)  • cerebellum (1)  • cerebellopontine angle (1)  • pineal (1) | NR | NR | NR |
| Cohen-Gadol et al, 2013 | VBAS | 5 (NR) | 35.0 (26-54) | • colloid cyst (5) | • intraventricular (5) | NR | 15.0mm (10-22mm) | NR |
| Akiyama et al, 2015 | VBAS | 16 (11/5) | 56.0 (26-81) | • germinoma (2)  • HGG (3)  • LGG (1)  • metastasis (2)  • neurocytoma (1)  • primary CNS lymphoma (7) | • frontal (6)  • lateral ventricle (3)  • thalamus (4)  • putamen (1)  • caudate (1)  • multifocal (1) | NR | NR | 13.9cm^3^ (4.3-48.6cm^3^) |
| Eliyas et al, 2016 | BrainPath | 20 (8/12) | 64.0 (19-74) | • colloid cyst (5)  • HGG (3)  • LGG (5)  • meningioma (2)  • metastasis (3)  • neurocytoma (1)  • primary CNS lymphoma (1) | • parietal (1)  • lateral ventricle (6)  • third ventricle (5)  • intraventricular (3)  • basal ganglia (1)  • thalamus (2)  • multifocal (1)  • periatrial (1) | 44.0mm (20-58mm) | NR | NR |
| Hong et al, 2016 | VBAS | 19 (12/7) | 55.8 (24-86) | • DNT or PNT (1)  • hemangioblastoma (1)  • HGG (6)  • LGG (1)  • meningioma (1)  • metastasis (8)  • neurocytoma (1) | • frontal (2)  • parietal (1)  • temporal (1)  • cerebellum (4)  • basal ganglia (9)  • multifocal (2) | NR | NR | NR |
| Kutlay et al, 2016 | Other (Modified Paediatric Anoscope) | 13 (7/6) | 49.6 (19-76) | • HGG (6)  • LGG (2)  • meningioma (3)  • metastasis (2) | • frontal (4)  • parietal (1)  • temporal (2)  • intraventricular (3)  • thalamus (1)  • multifocal (2) | NR | 45.5mm (33-65mm) | NR |
| Vaish et al, 2016 | Other (Tailored 5-ml Syringe) | 18 (6/12) | 34.9 (18-59) | • colloid cyst (18) | • third ventricle (18) | NR | NR | NR |
| Wright et al, 2016 | VBAS | 8 (4/4) | 66.8 (51-84) | • HGG (7)  • metastasis (1) | • frontal (3)  • parietal (1)  • corpus collosum (2)  • multifocal (2) | NR | NR | 35.4cm^3^ (10.6-69.6cm^3^) |
| Jackson et al, 2017 | BrainPath | 9 (6/3) | 51.0 (18-71) | • HGG (6)  • metastasis (1)  • primary CNS lymphoma (2) | • thalamus (2)  • optic pathway (2)  • intraventricular (1)  • centrum semiovale (1)  • multifocal (1) | NR | NR | 17.0cm^3^ (1.1-32.0cm^3^) |
| Sabanci et al, 2017 | Other (Cylindrical Retractor) | 18 (12/6) | 38.7 (20-62) | • colloid cyst (18) | • third ventricle (18) | NR | 15.5mm (7-22mm) | NR |
| Almubarak et al, 2018 | Other (Modified Plastic Syringe) | 8 (6/2) | 45.4 (24-75) | • HGG (8) | • temporal (1)  • basal ganglia (2)  • thalamus (2)  • insula (2)  • multifocal (1) | NR | NR | NR |
| Eichberg et al, 2018 | • BrainPath (12)  • VBAS (7) | 19 (10/9) | 56.2 (20-76) | • colloid cyst (6)  • HGG (2)  • LGG (1)  • metastasis (10) | • frontal (8)  • parietal (9)  • multifocal (2) | NR | NR | NR |
| Iyer er al, 2018 | BrainPath | 14 (10/4) | 42.2 (18-73) | • HGG (14) | • optic pathway (1)  • basal ganglia (1)  • thalamus (7)  • corticospinal tract (3)  • inferior frontal-occipital fasciculus (2) | NR | NR | 11.7cm^3^ (1.2-25.9cm^3^) |
| Mampre et al, 2018 | BrainPath | 13 (8/5) | 57.2 (23-78) | • hemangioblastoma (2)  • metastasis (11) | • cerebellum (13) | NR | NR | NR |
| Otani et al, 2018 | VBAS | 8 (5/3) | 56.0 (34-77) | • HGG (1)  • metastasis (2)  • neurocytoma (5) | • parietal (2)  • lateral ventricle (5)  • multifocal (1) | NR | NR | NR |
| Akbari et al, 2019 | BrainPath | 10 (8/2) | 53.2 (37-64) | • HGG (4)  • LGG (2)  • metastasis (4) | • frontal (6)  • temporal (2)  • occipital (1)  • lateral ventricle (1) | 26.0mm (12-35mm) | 29.0mm (12-43mm) | NR |
| Bakhsheshian et al, 2019 | BrainPath | 25 (12/13) | 61.0 (24-81) | • metastasis (25) | • frontal (5)  • parietal (8)  • occipital (3)  • cerebellum (8)  • corpus collosum (1) | • <20mm (6)  • 20-40mm (5)  • >40mm (14) | • 20-30mm (5)  • >30mm (20) | NR |
| Bander et al, 2019 | BrainPath | 18 (10/8) | 61.0 (NR) | • LGG or HGG (16)  • primary CNS lymphoma (2) | • frontal (3)  • parietal (5)  • temporal (5)  • multifocal (3)  • NR (2) | NR | NR | NR |
| Chakravarthi et al, 2019 | BrainPath | 12 (4/8) | 46.0 (20-74) | • craniopharyngioma (1)  • colloid cyst (6)  • epidermoid cyst (1)  • HGG (2)  • neurocytoma (2) | • lateral ventricle (1)  • third ventricle (6)  • intraventricular (1)  • multifocal (4) | NR | NR | 2.48cm^2^ (0.58-4.6cm^2^ |
| Eichberg et al, 2019 | • BrainPath (3)  • VBAS (7) | 10 (4/6) | 49.1 (20-74) | • colloid cyst (10) | • intraventricular (10) | NR | NR | NR |
| Gassie et al, 2019 | BrainPath | 15 (8/7) | 63.0 (SD ± 12) | • metastasis (15) | • cerebellum (3)  • basal ganglia (3)  • thalamus (2)  • centrum semiovale (7) | NR | NR | NR |
| Newman et al, 2019 | Other (Dilatable Endoscopic Port System) | 8 (2/6) | 51.1 (30-67) | • colloid cyst (6)  • meningioma (1)  • metastasis (1) | • parietal (1)  • third ventricle (6)  • lateral ventricle (1) | NR | NR | 17.3cm^3^ (0.4-65.4cm^3^) |
| Lin et al, 2020 | BrainPath | 4 (0/4) | 47.0 (37-54) | • meningioma (4) | • lateral ventricle (4) | NR | 25.5mm (22-28mm) | NR |
| Lin et al, 2020 | BrainPath | 16 (6/10) | 45.3 (23-62) | • colloid cyst (16) | • third ventricle (16) | NR | 14.0mm (7-28mm) | NR |
| Zagzoog et al, 2020 | METRx | 27 (NR) | NR | • colloid cyst (1)  • craniopharyngioma (1)  • epidermoid cyst (2)  • glioneural (1)  • HGG (4)  • LGG (7)  • LGG or HGG (2)  • meningioma (1)  • metastasis (5)  • neurocytoma (2)  • unspecified cyst (1) | • intraventricular (10)  • suprasellar (1)  • posterior fossa (1)  • cerebellopontine angle (2)  • parenchymal lobar (13) | NR | NR | NR |
| Guinto-Nishimura et al, 2021 | VBAS | 5 (3/2) | 42.6 (34-58) | • pituitary adenoma (5) | • multifocal (5) | NR | 61.2mm (48-81mm) | 56.5cm^3^ (20.2-97.0cm^3^) |
| Kutlay et al, 2021 | Other (Thoracoport) | 20 (12/8) | 51.2 (18-78) | • HGG (13)  • metastasis (7) | • lateral ventricle (11)  • third ventricle (1)  • thalamus (4)  • caudate nucleus (2)  • multifocal (2) | NR | NR | NR |
| Monroy-Sosa et al, 2021 | BrainPath | 11 (5/6) | 54.0 (18-57) | • HGG (3)  • LGG (1)  • metastasis (7) | • frontal (6)  • parietal (1)  • insular (1)  • multifocal (3) | 34.4mm (11-52mm) | NR | 4.3cm^3^ (2.7-6.4cm^3^) |
| Valarezo-Chuchuca et al, 2021 | VBAS | 9 (4/5) | 47.0 (19-75) | • HGG (3)  • LGG (2)  • LGG or HGG (1)  • metastasis (2)  • primary CNS lymphoma (1) | • frontal (2)  • intraventricular (1)  • cerebellum (3)  • thalamus (1)  • multifocal (2) | 35.0mm (18-45mm) | NR | 21.8cm^3^ (1.5-40.4cm^3^) |
| Achey et al, 2022 | Other (Aurora Surgiscope) | 1 (0/1) | 56.0 (56-56) | • LGG (1) | • cerebellum (1) | NR | 25.0mm (25-25mm) | NR |
| Gallagher et al, 2022 | BrainPath | 4 (2/2) | 56.5 (28-72) | • HGG (2)  • LGG (1)  • metastasis (1) | • frontal (1)  • parietal (1)  • temporal (1)  • optic pathway (1) | NR | NR | NR |
| Hajtovic et al, 2022 | VBAS | 22 (10/12) | 54.8 (21-82) | • HGG (22) | • frontal (1)  • parietal (3)  • temporal (2)  • occipital (4)  • basal ganglia (1)  • multifocal (11) | 23.0mm (0.3-4mm) | 42.0mm (2-8mm) | 33.1cm^3^ (1.3-73.0cm^3^) |
| Liu et al, 2022 | VBAS | 6 (0/6) | 52.3 (25-84) | • craniopharyngioma (2)  • germinoma (1)  • LGG (1)  • papilloma (1)  • pineal parenchymal cell tumour (1) | • third ventricle (6) | NR | 30.6mm (16-42mm) | NR |
| Liu et al, 2022 | BrainPath | 6 (4/2) | 53.0 (36-63) | • HGG (4)  • LGG (2) | • thalamus (6) | NR | 33.2mm (16-52mm) | NR |
| Sihag et al, 2022 | Other (Modified Silicon Retractor) | 19 (8/11) | 36.1 (19-60) | • HGG (8)  • LGG (11) | • thalamus (16)  • multifocal (3) | NR | NR | NR |
| Abdala-Vargas et al, 2023 | VBAS | 72 (46/26) | 49.6 (NR) | • HGG (25)  • LGG (21)  • metastasis (18)  • NR (8) | • frontal (19)  • parietal (8)  • multifocal (18)  • NR (27) | NR | NR | NR |
| Angileri et al, 2023 | VBAS | 7 (3/4) | 58.1 (36-70) | • HGG (2)  • meningioma (3)  • metastasis (2) | • temporal (1)  • lateral ventricle (3)  • intraventricular (2)  • central sulcus (1) | NR | 36.1mm (20-51mm) | 29.5cm^3^ (0.4-82.6cm^3^) |
| Sankhla et al, 2023 | Other (Transparent Plastic Tube) | 24 (10/14) | 40.5 (20-65) | • colloid cyst (3)  • DNT or PNT (1)  • HGG (2)  • LGG (6)  • LGG or HGG (5)  • neurocytoma (7) | • lateral ventricle (20)  • multifocal (5) | NR | NR | NR |
| Vitulli et al, 2023 | BrainPath | 7 (2/5) | 56.3 (26-79) | • HGG (1)  • LGG (1)  • metastasis (5) | • frontal (2)  • basal ganglia (2)  • cingulate (3) | 30.9mm (7-46mm) | NR | 13.2cm^3^ (3.3-32.6cm^3^) |
| Xie et al, 2023 | VBAS | 7 (2/5) | 47.0 (22-70) | • colloid cyst (2)  • LGG (1)  • LGG or HGG (1)  • metastasis (1)  • neuroblastoma (2) | • intraventricular (7) | NR | 35.0mm (20-60mm) | NR |
| Yan et al, 2023 | VBAS | 16 (5/11) | 43.2 (18-70) | • colloid cyst (1)  • HGG (1)  • LGG (3)  • LGG or HGG (1)  • meningioma (5)  • neurocytoma (5) | • lateral ventricle (16) | NR | NR | NR |

**Supplementary Data 4 – Surgical Characteristics and Outcomes**

**Summary of surgical characteristics and outcomes of included studies**.

*ALA = aminolaevulinic acid; CSF = cerebrospinal fluid; DTI = diffusion tensor imaging; EOR = extent of resection; fMRI = functional MRI; GTR = gross total resection; IONM = intra-operative neurophysiological monitoring; LITT = laser interstitial thermal therapy; METRx = minimal exposure tubular retractor system; NR = not reported; nTMS = navigated transcranial magnetic stimulation; NTR = near total resection; N/A = not applicable; STR = subtotal resection; VBAS = viewsite brain access system.*

| **Reference** | **Retractor Brand(s)** | **Mean Retractor Dimensions (Range)** | **Surgical Approach** | **Visualisation** | **Surgical Adjuncts** | **Mean EOR (Range)** | **Detailed EOR Data** | **Detailed Complications Data (n)** | **Mean Operative Time (Range)** | **Mean Post-Operative Hospital Stay (Range)** |
| --- | --- | --- | --- | --- | --- | --- | --- | --- | --- | --- |
| Abernathey et al, 1989 | Other (Cylindrical Retractor) | • Diameter: 20mm (20-20mm)  • Length NR (NR) | Trans-cortical | • Microscope | — | 100% (100-100%) | • GTR: 12/12 | 5/12 (41.7%)  • aseptic meningitis (1)  • aseptic meningitis + memory disturbance (1)  • dilantin toxicity (1)  • subdural hygroma (1)  • hydrocephalus (1) | NR | NR |
| Cabbell et al, 1996 | Other (Cylindrical Retractor) | • Diameter: 16mm (16-16mm)  • Length: NR (NR) | Trans-cortical | • Microscope | — | NR (NR) | NR | 3/15 (20%)  • transient cognitive and memory deficits (1)  • subdural hygroma (1)  • subgaleal fluid collection (1) | 129.0 minutes (90-162 minutes) | NR |
| Barlas et al, 2004 | Other (Cylindrical Retractor) | • Diameter: 14mm (14-14mm)  • Length: NR (NR) | Trans-cortical | • Microscope | — | 100% (100-100%) | • GTR: 14/14 | 1/14 (7.1%)  • meningitis (1) | NR | NR |
| Ogura et al, 2006 | Other (Transparent Plastic Tube) | • Diameter: 20mm (20-20mm)  • Length: NR (5-7cm) | Trans-cortical | • Microscope | • intra-operative ultrasound  • intra-operative fibreoptic pressure monitor | NR (NR) | • GTR: 3/6  • STR: 1/6  • Biopsy: 2/6 | 0/6 (0.0%) | NR | NR |
| Greenfield et al, 2008 | METRx | • Diameter: 18mm (14-22mm)  • Length: 4.4cm (3-7cm) | Trans-cortical | • Microscope | — | 100% (100-100%) | • GTR: 9/9 | 1/9 (11.1%)  • transient worsening of pre-operative aphasia (1) | 149.3 minutes (93-216 minutes) | 5.0 days (2-8 days) |
| Kassam et al, 2009 | Other (Omni Transparent Conduit) | • Diameter: 11.5mm (11.5-11.5mm)  • Length: NR (5.5-6.5cm) | Trans-cortical | • Endoscope | — | NR (NR) | • GTR: 5/18  • NTR: 6/18  • STR: 7/18 | 3/18 (16.7%)  • brain abscess + pulmonary embolus (1)  • second corticectomy and cannulation (1)  • haemothorax (1) | NR | NR |
| Jo et al, 2011 | Other (Modified Tubular Conduit) | • Diameter: 11mm (11-11mm)  • Length: NR (5-6cm) | Trans-cortical | • Endoscope | • pre-operative fMRI or DTI | NR (NR) | • GTR: 9/14  • STR: 3/14  • Failure: 2/14 | 0/14 (0.0%) | NR | NR |
| Almenawer et al, 2013 | METRx | • Diameter: 18mm (18-18mm)  • Length: NR (3-9cm) | NR | • Microscope  • Endoscope | — | NR (NR) | • GTR: 20/27  • STR: 7/27 | 2/27 (7.4%)  • hydrocephalus (1)  • CSF leak (1) | NR | 5.9 days (1-18 days) |
| Cohen-Gadol et al, 2013 | VBAS | • Diameter: 12mm (12-12mm)  • Length: 5cm (5-5cm) | Trans-cortical | • Microscope | — | 100% (100-100%) | • GTR: 5/5 | 0/5 (0.0%) | NR | NR |
| Akiyama et al, 2015 | VBAS | • Diameter: 17mm (17-17mm)  • Length: 7cm (7-7cm) | Trans-cortical | • Endoscope | — | NR (NR) | • GTR: 4/16  • STR: 4/16  • Biopsy: 8/16 | 0/16 (0.0%) | NR | NR |
| Eliyas et al, 2016 | BrainPath | • Diameter: 13.5mm (13.5-13.5mm)  • Length: NR (NR) | Trans-sulcal | • Microscope  • Exoscope | • pre-operative DTI  • NICO Myriad | 96.6% (75-100%) | • GTR: 15/20  • NTR: 2/20  • STR: 3/20 | 4/20 (20%)  • weakness (1)  • short-term memory loss (2)  • permanent CSF diversion (1) | NR | NR |
| Hong et al, 2016 | VBAS | • Diameter: NR (12-17mm)  • Length: NR (5-7cm) | • Trans-cortical  • Trans-sulcal | • Microscope  • Endoscope | — | NR (NR) | • GTR: 13/19  • NTR: 4/19  • STR: 2/19 | NR | NR | NR |
| Kutlay et al, 2016 | Other (Modified Paediatric Anoscope) | • Diameter: 18mm (18-18mm)  • Length: 5.4cm (5.4-5.4cm) | • Trans-cortical  • Trans-sulcal | • Endoscope | — | NR (NR) | • GTR: 7/13  • NTR: 3/13  • STR: 3/13 | 0/13 (0.0%) | 90.0 minutes (60-110 minutes) | 2.7 days (NR) |
| Vaish et al, 2016 | Other (Tailored 5-ml Syringe) | • Diameter: 13mm (13-13mm)  • Length: NR (NR) | Trans-cortical | • Microscope | — | 100% (100-100%) | • GTR: 18/18 | NR | NR | 4.0 days (3-7 days) |
| Wright et al, 2016 | VBAS | • Diameter: NR (NR)  • Length: NR (NR) | Trans-sulcal | • Microscope | • pre-operative LITT  • pre-operative DTI  • simulation via Surgical Theatre | 91.9% (85-100%) | • GTR: 1/8  • NTR: 2/8  • STR: 5/8 | 3/8 (37.5%)  • imbalance (1)  • pin site infection (1)  • hydrocephalus (1) | NR | NR |
| Jackson et al, 2017 | BrainPath | • Diameter: NR (NR)  • Length: NR (NR) | • Trans-cortical  • Trans-sulcal | • Exoscope | • pre-operative DTI  • intra-operative ultrasound  • NICO Myriad | N/A | • Biopsy: 9/9 | 0/9 (0.0%) | NR | NR |
| Sabanci et al, 2017 | Other (Cylindrical Retractor) | • Diameter: 14mm (14-14mm)  • Length: NR (NR) | Trans-cortical | • Endoscope | — | 100% (100-100%) | • GTR: 18/18 | 3/18 (16.7%)  • meningitis (2)  • extradural haematoma (1) | NR | NR |
| Almubarak et al, 2018 | Other (Modified Plastic Syringe) | • Diameter: 17mm (17-17mm)  • Length: NR (NR) | • Trans-cortical  • Trans-sulcal | • Microscope | • pre-operative DTI  • IONM | NR (NR) | • GTR: 2/8  • STR: 6/8 | 3/8 (37.5%)  • transient aphasia + weakness (2)  • transient weakness + CN VI palsy (1) | NR | NR |
| Eichberg et al, 2018 | • BrainPath (12)  • VBAS (7) | • Diameter: NR (NR)  • Length: NR (NR) | • Trans-cortical  • Trans-sulcal | • Endoscope | • pre-operative DTI | NR (NR) | • GTR: 18/19  • STR: 1/19 | 2/18 (10.5%)  • transient short-term memory difficulty (1)  • stroke (1) | NR | NR |
| Iyer er al, 2018 | BrainPath | • Diameter: NR (NR)  • Length: (NR) | Trans-sulcal | • Exoscope | • pre-operative DTI  • IONM  • intra-operative ultrasound  • NICO Myriad | 97.0% (SD ± 1.2%) | • GTR: 7/14  • NTR: 4/14  • STR: 3/14 | 1/14 (7.1%)  • worsened upper extremity weakness (1) | NR | 4.0 days (2-7 days) |
| Mampre et al, 2018 | BrainPath | • Diameter: NR (NR)  • Length: NR (NR) | NR | • Exoscope | • intra-operative ultrasound  • NICO Myriad | 96.5% (84-100%) | • GTR: 9/13  • STR: 4/13 | 0/13 (0.0%) | NR | NR |
| Otani et al, 2018 | VBAS | • Diameter: NR (NR)  • Length: NR (NR) | Trans-cortical | • Microscope  • Endoscope | • pre-operative fMRI or DTI | NR (NR) | • GTR: 3/8  • NTR: 4/8  • STR: 1/8 | 0/8 (0.0%) | NR | NR |
| Akbari et al, 2019 | BrainPath | • Diameter: NR (NR)  • Length: NR (NR) | Trans-sulcal | • Exoscope | • pre-operative fMRI or DTI  • intra-operative MRI  • IONM  • NICO Myriad | NR (NR) | • GTR: 8/10  • STR: 2/10 | 3/10 (30.0%)  • diplopia, hemiparesis, aphasia (1)  • hemiparesis, neglect (1)  • hemiparesis, aphasia (1) | 335.7 minutes (230-440 minutes) | 3.4 days (1-7 days) |
| Bakhsheshian et al, 2019 | BrainPath | • Diameter: NR (NR)  • Length: NR (NR) | Trans-sulcal | • Exoscope | • pre-operative DTI  • NICO Myriad | NR (NR) | • GTR: 20/25  • STR: 5/25 | 1/25 (4.0%)  • hemiparesis with subsequent DVT (1) | NR | 3.5 days (1-12 days) |
| Bander et al, 2019 | BrainPath | • Diameter: NR (NR)  • Length: NR (NR) | Trans-sulcal | • Exoscope | • pre-operative fMRI or DTI  • pre-operative perfusion analysis | N/A | • Biopsy: 18/18 | 1/18 (5.6%)  • haemorrhage (1) | NR | NR |
| Chakravarthi et al, 2019 | BrainPath | • Diameter: NR (NR)  • Length: NR (NR) | Trans-sulcal | • Microscope | • pre-operative DTI  • IONM  • intra-operative awake neurological testing | NR (NR) | • GTR: 7/12  • STR: 2/12  • Biopsy: 3/12 | NR | 180.0 minutes (90-302 minutes) | 6.1 days (2-20 days) |
| Eichberg et al, 2019 | • BrainPath (3)  • VBAS (7) | BrainPath  • Diameter: NR (NR)  • Length: NR (NR)  VBAS  • Diameter: 17mm (17-17mm)  • Length: 7cm (7-7cm) | Trans-sulcal | • Microscope | — | 100% (100-100%) | • GTR: 10/10 | 1/10 (10.0%)  • short-term memory impairment (1) | NR | 2.0 days (1-4 days) |
| Gassie et al, 2019 | BrainPath | • Diameter: NR (NR)  • Length: NR (NR) | Trans-sulcal | • Exoscope | • pre-operative DTI  • intra-operative ultrasound  • NICO Myriad | 99.7% (95.7-100%) | • GTR: 14/15  • NTR: 1/15 | 2/15 (13.3%)  • DVT (1)  • worsened leg weakness (1) | NR | 2.8 days* (2-5 days)  *median |
| Newman et al, 2019 | Other (Dilatable Endoscopic Port System) | • Diameter: 14mm (14-14mm)  • Length: 4cm (4-4cm) | Trans-sulcal | • Endoscope | — | 100% (100-100%) | • GTR: 8/8 | 3/8 (37.5%)  • hydrocephalus (1)  • haemorrhage (1)  • haematoma (1) | NR | NR |
| Lin et al, 2020 | BrainPath | • Diameter: NR (NR)  • Length: 5.8cm (5-6cm) | Trans-sulcal | • Exoscope | • IONM  • NICO Myriad | 100% (100-100%) | • GTR: 4/4 | 2/4 (50.0%)  • homonymous hemianopsia (1)  • lower extremity paraesthesia (1) | NR | 3.3 days (3-4 days) |
| Lin et al, 2020 | BrainPath | • Diameter: NR (NR)  • Length: 6.4cm (5-7.5cm) | Trans-sulcal | • Microscope  • Exoscope | — | 100% (100-100%) | • GTR: 16/16 | 3/16 (18.8%)  • transient memory deficit (2)  • transient memory deficit + pulmonary embolus (1) | NR | 4.0 days* (2-18 days)  *median |
| Zagzoog et al, 2020 | METRx | • Diameter: NR (14-18mm)  • Length: NR (NR) | Trans-cortical | • Microscope | • pre-operative DTI | NR (NR) | • GTR: 13/27  • STR: 14/27 | 9/27 (33.3%)  • neurological deficit (6)  • infection (meningitis, ventriculitis) (2)  • pulmonary embolus (1) | NR | 6.4 days (1-20 days) |
| Guinto-Nishimura et al, 2021 | VBAS | • Diameter: NR (NR)  • Length: NR (NR) | Trans-sulcal | • Microscope  • Endoscope  • Exoscope | — | 94.6% (89-98%) | • NTR: 3/5  • STR: 2/5 | 1/5 (20.0%)  • CSF leak (1) | NR | NR |
| Kutlay et al, 2021 | Other (Thoracoport) | • Diameter: NR (NR)  • Length: NR (NR) | NR | • Endoscope | • intra-operative fluorescein | NR (NR) | • GTR: 16/20  • NTR: 3/20  • STR: 1/20 | 0/20 (0.0%) | 122.1 minutes (65-176 minutes) | 3.9 days (3-6 days) |
| Monroy-Sosa et al, 2021 | BrainPath | • Diameter: 13.5 mm (13.5-13.5mm)  • Length: NR (5-7.5 cm) | Trans-sulcal | • Microscope | • pre-operative DTI  • IONM  • intra-operative awake neurological testing | NR (NR) | • GTR: 7/11  • NTR: 3/11  • STR: 1/11 | 3/11 (27.3%)  • superior monoparesis (1)  • hemiparesis and dysphasia (1)  • hypoesthesia (1) | NR | 2.7 days (2-5 days) |
| Valarezo-Chuchuca et al, 2021 | VBAS | • Diameter: NR (17-21mm)  • Length: NR (5-7cm) | Trans-cortical | • Microscope  • Endoscope | • pre-operative DTI | NR (NR) | • GTR: 7/9  • STR: 2/9 | 0/9 (0.0%) | NR | NR |
| Achey et al, 2022 | Other (Aurora Surgiscope) | • Diameter: 15mm (15-15mm)  • Length: NR (NR) | NR | • Endoscope | — | 100% (100-100%) | • GTR: 1/1 | 0/1 (0.0%) | 172.0 minutes (172-172 minutes) | NR |
| Gallagher et al, 2022 | BrainPath | • Diameter: 13.5mm (13.5-13.5mm)  • Length: 6.6cm (6-7.5cm) | Trans-sulcal | • Microscope  • Endoscope | • pre-operative transcranial magnetic stimulation  • pre-operative fMRI or DTI  • IONM  • intra-operative ultrasound  • NICO Myriad  • 5-ALA | NR (NR) | • GTR: 2/4  • NTR: 1/4  • STR: 1/4 | 1/4 (25.0%)  • word finding difficulties and visual agnosia (1) | NR | NR |
| Hajtovic et al, 2022 | VBAS | • Diameter: 18.8mm (12-21mm)  • Length: 6.1cm (5-7cm) | Trans-sulcal | • Microscope | • pre-operative DTI  • simulation via Surgical Theatre  • intra-operative MRI  • IONM  • intra-operative stimulation for language mapping  • intra-operative fluorescein | 73.4% (4.7-100%) | • GTR: 5/22  • NTR: 6/22  • STR: 11/22 | 7/22 (31.8%)  • worsening visual deficit (2)  • agitation (1)  • transient visual deficit (1)  • new visual deficit + agitation + dysphasia (1)  • wound infection + pulmonary embolus + new visual deficit + worsening alexia (1)  • pneumonia + worsening hemiparesis + worsening aphasia (1) | NR | 5.5 days (2-22 days) |
| Liu et al, 2022 | VBAS | • Diameter: 21mm (21-21mm)  • Length: 7cm (7-7cm) | Trans-cortical | • Endoscope | — | NR (NR) | • GTR: 5/6  • STR: 1/6 | 0/6 (0.0%) | NR | NR |
| Liu et al, 2022 | BrainPath | • Diameter: NR (21-28mm)  • Length: 7cm (7-7cm) | Trans-cortical | • Endoscope | — | NR (NR) | • GTR: 2/6  • STR: 4/6 | 5/6 (83.3%)  • electrolyte imbalance (2)  • subdural haematoma (1)  • pneumonia (1)  • DVT (1) | NR | 3.2 days (1-7 days) |
| Sihag et al, 2022 | Other (Modified Silicon Retractor) | • Diameter: 18mm (18-18mm)  • Length: NR (NR) | • Trans-cortical  • Trans-sulcal | • Endoscope | — | NR (NR) | • GTR: 8/19  • NTR: 8/19  • STR: 3/19 | 3/19 (15.8%)  • motor deficit (1)  • speech deficit (1)  • brain contusion (1) | 157.1 minutes (110-210 minutes) | NR |
| Abdala-Vargas et al, 2023 | VBAS | • Diameter: NR (NR)  • Length: NR (NR) | Trans-sulcal | NR | • pre-operative DTI  • intra-operative cortical stimulation | NR (NR) | • GTR: 68/72  • STR: 4/72 | 7/72 (9.7%)  • impaired muscle strength (4)  • aphasia (3) | NR | NR |
| Angileri et al, 2023 | VBAS | • Diameter: NR (17-28mm)  • Length: NR (5-7cm) | Trans-sulcal | • Microscope | • pre-operative DTI  • pre-operative nTMS mapping | NR (NR) | • GTR: 5/7  • STR: 1/7  • Biopsy: 1/7 | 3/7 (42.9%)  • visual field deficit (1)  • sensory aphasia (2) | NR | NR |
| Sankhla et al, 2023 | Other (Transparent Plastic Tube) | • Diameter: 11mm (11-11mm)  • Length: NR (NR) | Trans-cortical | • Endoscope | — | NR (NR) | • GTR: 16/24  • STR: 8/24 | 11/24 (45.8%)  • seizures, hemiparesis (1)  • visual field defect (1)  • persistent hydrocephalus (3)  • seizures, subdural hygroma (1)  • persistent hydrocephalus, seizures (1)  • persistent hydrocephalus, memory impairment (2)  • persistent hydrocephalus, hemiparesis (1)  • subdural hygroma, seizures, visual field deficit (1) | NR | NR |
| Vitulli et al, 2023 | BrainPath | • Diameter: NR (NR)  • Length: NR (NR) | Trans-sulcal | NR | • pre-operative DTI  • IONM  • NICO Myriad | NR (NR) | • GTR: 3/7  • STR: 4/7 | 7/7 (100.0%)  • decreased motor power (7) | NR | 18.5 days (3-56 days) |
| Xie et al, 2023 | VBAS | • Diameter: NR (12-28mm)  • Length: NR (3-7cm) | • Trans-cortical  • Trans-sulcal | • Endoscope | — | 100% (100-100%) | • GTR: 7/7 | 1/7 (14.3%)  • haematoma (1) | NR | NR |
| Yan et al, 2023 | VBAS | • Diameter: 21mm (21-21mm)  • Length: 7cm (7-7cm) | Trans-cortical | • Endoscope | — | NR (NR) | • GTR: 14/16  • STR: 2/16 | 4/16 (25.0%)  • transient hemiplegia (1)  • epidural haematoma (1)  • obstructive hydrocephalus (2) | NR | NR |

**Supplementary Data 5 – Extent of Resection (EOR) based on Tumour Histology and Location**

**Extent of resection based on tumour histology and location**.

*DNT = dysembryoplastic neuroepithelial tumour; GTR = gross total resection; HGG = high-grade glioma; LGG = low-grade glioma; NR = not reported; NTR = near total resection; PNT = primitive neuroectodermal tumour; STR = subtotal resection.*

| **Histology** | **GTR (100%)** | **NTR/STR/Failure (<100%)** | **Total Patients** |  |
| --- | --- | --- | --- | --- |
| Gliomas | 110 | 99 | 209 |  |
| Metastases | 103 | 33 | 136 |  |
| Colloid Cysts | 125 | 0 | 125 |  |
| **Retractor Brand Comparison** | | **χ^2^ difference in rate of GTR (p value)** | | |
| Gliomas vs Metastases | | 2.6520 x 10^-5^ | | |
| Gliomas vs Colloid Cysts | | 2.2000 x 10^-16^ | | |
| Metastases vs Colloid Cysts | | 1.1560 x 10^-11^ | | |

| **Histology** | **GTR (100%)** | **NTR/STR/Failure (<100%)** | **Total Patients** |  |
| --- | --- | --- | --- | --- |
| LGG | 37 | 18 | 55 |  |
| HGG | 66 | 78 | 144 |  |
| **Retractor Brand Comparison** | | **χ^2^ difference in rate of GTR (p value)** | | |
| LGG vs HGG | | 0.01083 | | |

| **Histology** | **GTR (100%)** | **NTR (95-99%)** | **STR (<95%)** | **Failure** | **Biopsy** | **Total Patients** |
| --- | --- | --- | --- | --- | --- | --- |
| HGG | 66 | 26 | 52 | 0 | 10 | 154 |
| LGG | 37 | 4 | 14 | 0 | 0 | 55 |
| LGG or HGG | 7 | 0 | 3 | 0 | 16 | 26 |
| Metastasis | 103 | 10 | 22 | 1 | 2 | 138 |
| Colloid cyst | 125 | 0 | 0 | 0 | 0 | 125 |
| Neurocytoma | 15 | 5 | 8 | 1 | 0 | 29 |
| Meningioma | 24 | 0 | 0 | 0 | 0 | 24 |
| Primary CNS lymphoma | 1 | 1 | 2 | 0 | 10 | 14 |
| Epidermoid cyst | 2 | 0 | 3 | 0 | 1 | 6 |
| Pituitary adenoma | 0 | 2 | 4 | 0 | 0 | 6 |
| Craniopharyngioma | 2 | 0 | 3 | 0 | 0 | 5 |
| Hemangioblastoma | 3 | 1 | 0 | 0 | 0 | 4 |
| Germinoma | 0 | 0 | 1 | 0 | 2 | 3 |
| DNT or PNT | 1 | 0 | 1 | 0 | 0 | 2 |
| Neuroblastoma | 2 | 0 | 0 | 0 | 0 | 2 |
| Cyst (not specified) | 0 | 0 | 1 | 0 | 0 | 1 |
| Glioneural tumour | 0 | 0 | 1 | 0 | 0 | 1 |
| Papilloma | 1 | 0 | 0 | 0 | 0 | 1 |
| Pineal parenchymal cell tumour | 1 | 0 | 0 | 0 | 0 | 1 |
| **Total** | **390** | **49** | **115** | **2** | **41** | **597** |

Cabbell et al (1996) and Abdala-Vargas et al (2023) were excluded from this analysis due to having no detailed histology EOR data.

| **Location** | **GTR (100%)** | **NTR (95-99%)** | **STR (<95%)** | **Failure** | **Biopsy** | **Total Patients** |
| --- | --- | --- | --- | --- | --- | --- |
| Ventricular | 193 | 7 | 16 | 2 | 4 | 222 |
| Frontal lobe | 42 | 5 | 13 | 0 | 5 | 65 |
| Thalamus | 18 | 12 | 14 | 0 | 4 | 48 |
| Parietal lobe | 30 | 4 | 5 | 0 | 5 | 44 |
| Cerebellum | 30 | 2 | 8 | 0 | 0 | 40 |
| Basal ganglia | 15 | 2 | 6 | 0 | 2 | 25 |
| Temporal lobe | 13 | 3 | 3 | 0 | 5 | 24 |
| Occipital lobe | 8 | 0 | 1 | 0 | 0 | 9 |
| Centrum semiovale | 6 | 1 | 0 | 0 | 1 | 8 |
| Optic pathway | 1 | 0 | 1 | 0 | 2 | 4 |
| Cingulate | 0 | 0 | 3 | 0 | 0 | 3 |
| Corpus collosum | 1 | 0 | 2 | 0 | 0 | 3 |
| Corticospinal tract | 2 | 0 | 1 | 0 | 0 | 3 |
| Insular | 3 | 0 | 0 | 0 | 0 | 3 |
| Inferior frontal-occipital fasciculus | 2 | 0 | 0 | 0 | 0 | 2 |
| Central sulcus | 1 | 0 | 0 | 0 | 0 | 1 |
| Cerebellopontine angle | 0 | 0 | 1 | 0 | 0 | 1 |
| Periatrial | 1 | 0 | 0 | 0 | 0 | 1 |
| Pineal | 0 | 0 | 1 | 0 | 0 | 1 |
| Multifocal | 11 | 13 | 26 | 0 | 11 | 61 |
| Other (e.g. specifics NR) | 0 | 0 | 0 | 0 | 2 | 2 |
| **Total** | **377** | **49** | **101** | **2** | **41** | **570** |

Cabbell et al (1996), Zagzoog et al (2020), and Abdala-Vargas et al (2023) were excluded from this analysis due to having no detailed location EOR data.

**Supplementary Data 6 – Extent of Resection (EOR) based on Brand of Tubular Retractor**

**Extent of resection based on brand of tubular retractor**.

*GTR = gross total resection; METRx = minimal exposure tubular retractor system; NTR = near total resection; STR = subtotal resection; VBAS = viewsite brain access system.*

| **Retractor Brand** | **GTR (100%)** | **NTR/STR/Failure (<100%)** | **Total Patients** |  |
| --- | --- | --- | --- | --- |
| VBAS | 143 | 55 | 198 |  |
| BrainPath | 136 | 40 | 176 |  |
| METRx | 42 | 21 | 63 |  |
| Other | 137 | 54 | 191 |  |
| **Retractor Brand Comparison** | | **χ^2^ difference in rate of GTR (p value)** | | |
| VBAS vs BrainPath | | 0.3169 | | |
| VBAS vs METRx | | 0.4926 | | |
| VBAS vs Other | | 1.0000 | | |
| BrainPath vs METRx | | 0.1366 | | |
| BrainPath vs Other | | 0.2730 | | |
| METRx vs Other | | 0.5456 | | |

**Supplementary Data 7 – Complications based on Tumour Histology and Location**

**Complications based on tumour histology and location**.

*DNT = dysembryoplastic neuroepithelial tumour; HGG = high-grade glioma; LGG = low-grade glioma; NR = not reported; PNT = primitive neuroectodermal tumour.*

| **Histology** | **Number of Complications** | **Number Complication-Free** | **Total Patients** |
| --- | --- | --- | --- |
| Gliomas | 40 | 145 | 185 |
| Metastases | 18 | 130 | 148 |
| Colloid Cysts | 20 | 115 | 135 |
| **Retractor Brand Comparison** | | **χ^2^ difference in rate of complications (p value)** | |
| Gliomas vs Metastases | | 0.0343 | |
| Gliomas vs Colloid Cysts | | 0.1628 | |
| Metastases vs Colloid Cysts | | 0.6318 | |

| **Histology** | **Number of Complications** | **Number Complication-Free** | **Total Patients** |
| --- | --- | --- | --- |
| LGG | 14 | 33 | 47 |
| HGG | 24 | 109 | 133 |
| **Retractor Brand Comparison** | | **χ^2^ difference in rate of complications (p value)** | |
| LGG vs HGG | | 0.1368 | |

| **Histology** | **Number of Complications** | **Total Patients** |
| --- | --- | --- |
| HGG | 24 | 133 |
| LGG | 14 | 47 |
| LGG or HGG | 2 | 8 |
| Metastasis | 18 | 130 |
| Colloid cyst | 20 | 115 |
| Neurocytoma | 4 | 24 |
| Meningioma | 3 | 19 |
| Primary CNS lymphoma | 0 | 12 |
| Pituitary adenoma | 1 | 6 |
| Craniopharyngioma | 1 | 3 |
| Epidermoid cyst | 0 | 3 |
| Germinoma | 0 | 3 |
| Hemangioblastoma | 0 | 3 |
| Neuroblastoma | 1 | 2 |
| DNT or PNT | 1 | 1 |
| Papilloma | 0 | 1 |
| Pineal parenchymal cell tumour | 0 | 1 |
| **Total** | **89** | **511** |

Hong et al (2016), Vaish et al (2016), Bander et al (2019), Chakravarthi et al (2019), Zagzoog et al (2020), Abdala-Vargas et al (2023), and Angileri et al (2023) were excluded from this analysis due to having no detailed histology complication data.

| **Location** | **Number of Complications** | **Total Patients** |
| --- | --- | --- |
| Ventricular | 37 | 206 |
| Frontal lobe | 11 | 59 |
| Thalamus | 8 | 46 |
| Parietal lobe | 2 | 35 |
| Cerebellum | 2 | 33 |
| Temporal lobe | 2 | 15 |
| Basal ganglia | 4 | 12 |
| Occipital lobe | 0 | 5 |
| Optic pathway | 0 | 4 |
| Cingulate | 1 | 3 |
| Corpus collosum | 0 | 3 |
| Corticospinal tract | 0 | 3 |
| Insular | 2 | 3 |
| Inferior frontal-occipital fasciculus | 0 | 2 |
| Centrum semiovale | 0 | 1 |
| Cerebellopontine angle | 0 | 1 |
| Periatrial | 0 | 1 |
| Pineal | 1 | 1 |
| Multifocal | 8 | 41 |
| **Total** | **78** | **474** |

Hong et al (2016), Vaish et al (2016), Bander et al (2019), Chakravarthi et al (2019), Gassie et al (2019), Zagzoog et al (2020), Hajtovic et al (2022), Abdala-Vargas et al (2023), and Angileri et al (2023) were excluded from this analysis due to having no detailed location complication data.

**Supplementary Data 8 – Complications based on Brand of Tubular Retractor**

**Complications based on brand of tubular retractor**.

*METRx = minimal exposure tubular retractor system; VBAS = viewsite brain access system.*

| **Retractor Brand** | **Number of Complications** | **Number Complication-Free** | **Total Patients** |
| --- | --- | --- | --- |
| VBAS | 22 | 159 | 181 |
| BrainPath | 34 | 157 | 191 |
| METRx | 12 | 53 | 65 |
| Other | 35 | 155 | 190 |
| **Retractor Brand Comparison** | | **χ^2^ difference in rate of complications (p value)** | |
| VBAS vs BrainPath | | 0.1685 | |
| VBAS vs METRx | | 0.2918 | |
| VBAS vs Other | | 0.1262 | |
| BrainPath vs METRx | | 1.0000 | |
| BrainPath vs Other | | 0.9808 | |
| METRx vs Other | | 1.0000 | |

**Supplementary Data 9 – Risk of Methodological Bias Assessment**

**JBI Critical Appraisal Checklist for Case Series**


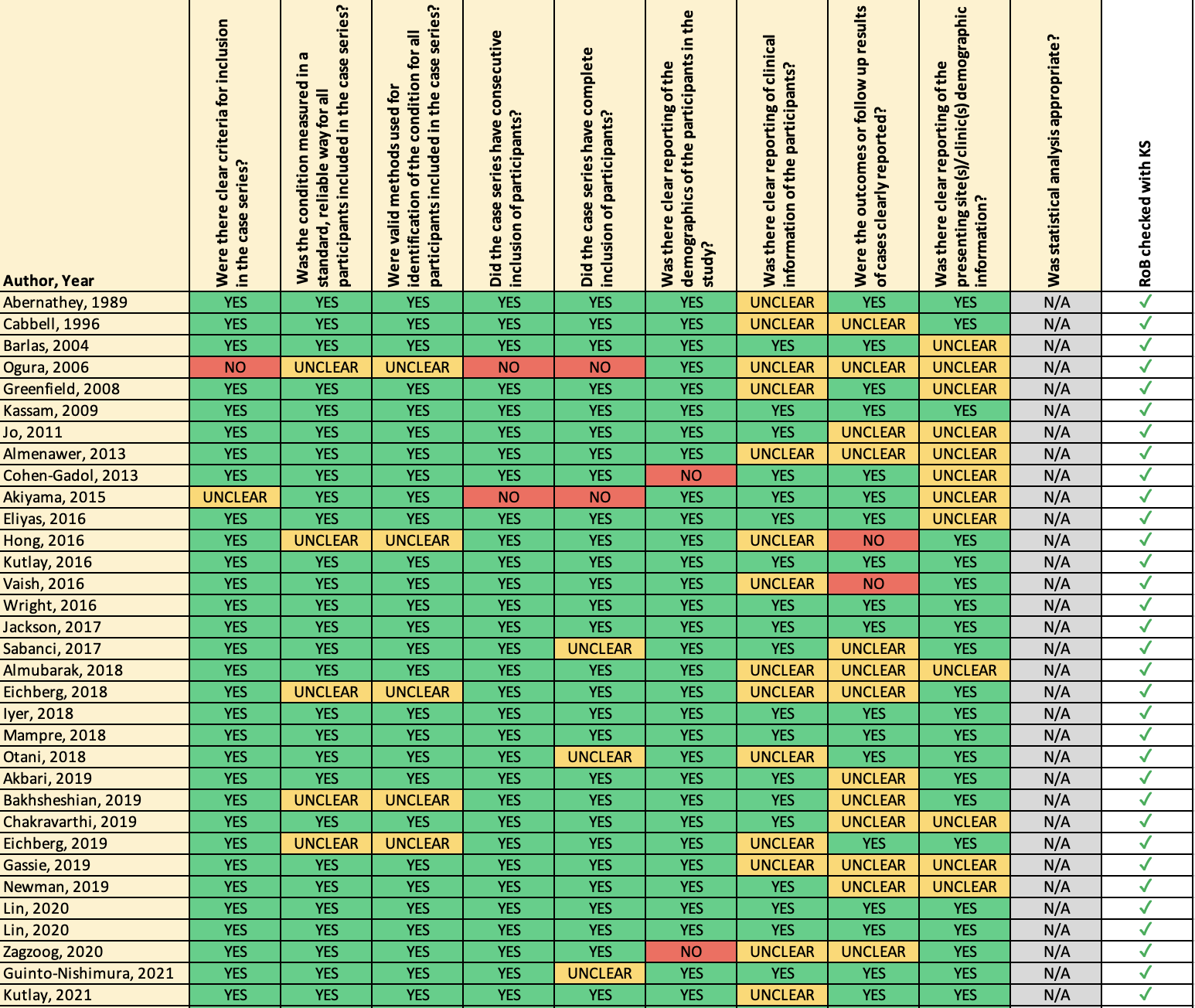


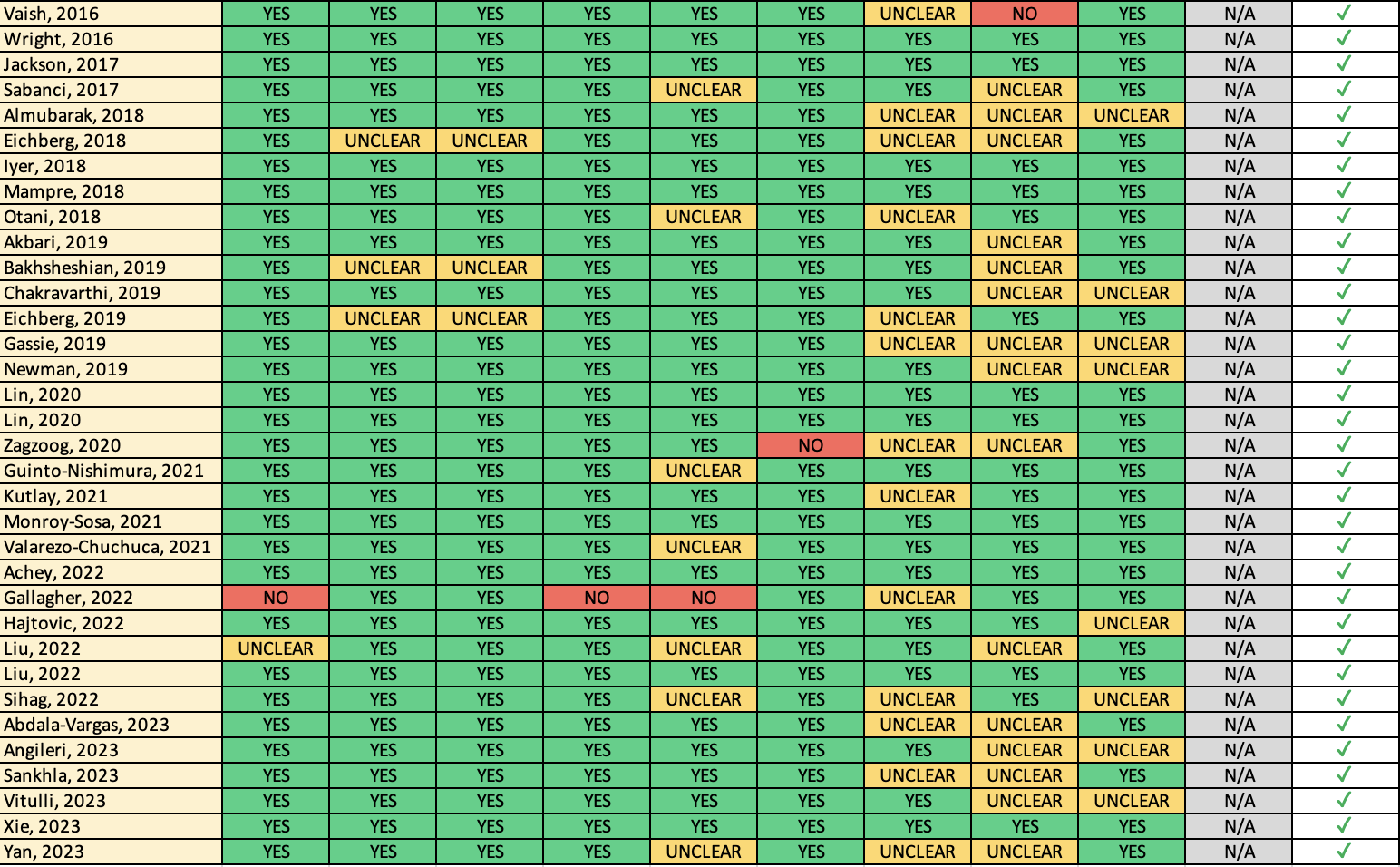


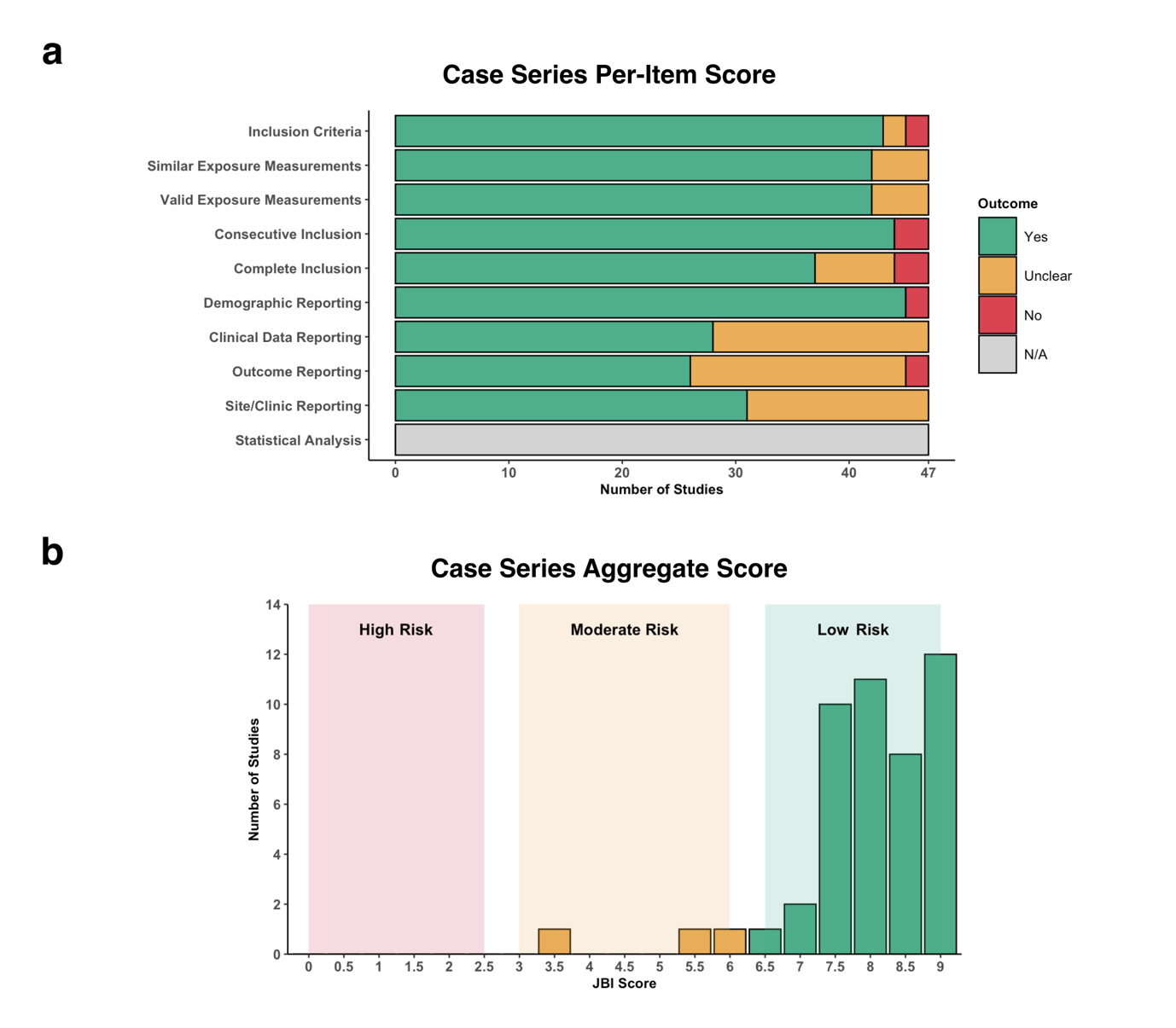


**Risk of bias assessment for case series studies.** a. Histogram of distribution of per-item scores for case series studies, indicating for each item, the number of articles scoring ‘Yes’, ‘Unclear’, ‘No’ or ‘N/A’. b. Histogram of distribution of aggregate JBI scores for case series studies. ‘Yes’ scored 1 point per item, ‘Unclear’ scored 0.5 points per item and ‘No’ scored 0 points per item.

*METRx = minimal exposure tubular retractor system; VBAS = viewsite brain access system.*

**JBI Critical Appraisal Checklist for Case Series**


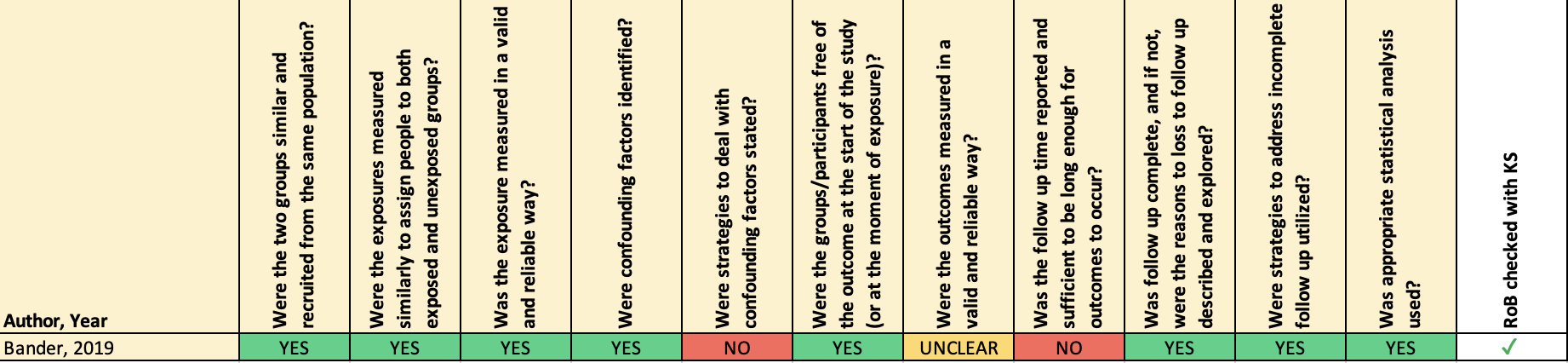


**Supplementary Data 10 – Publication Bias Assessment using Funnel Plots**


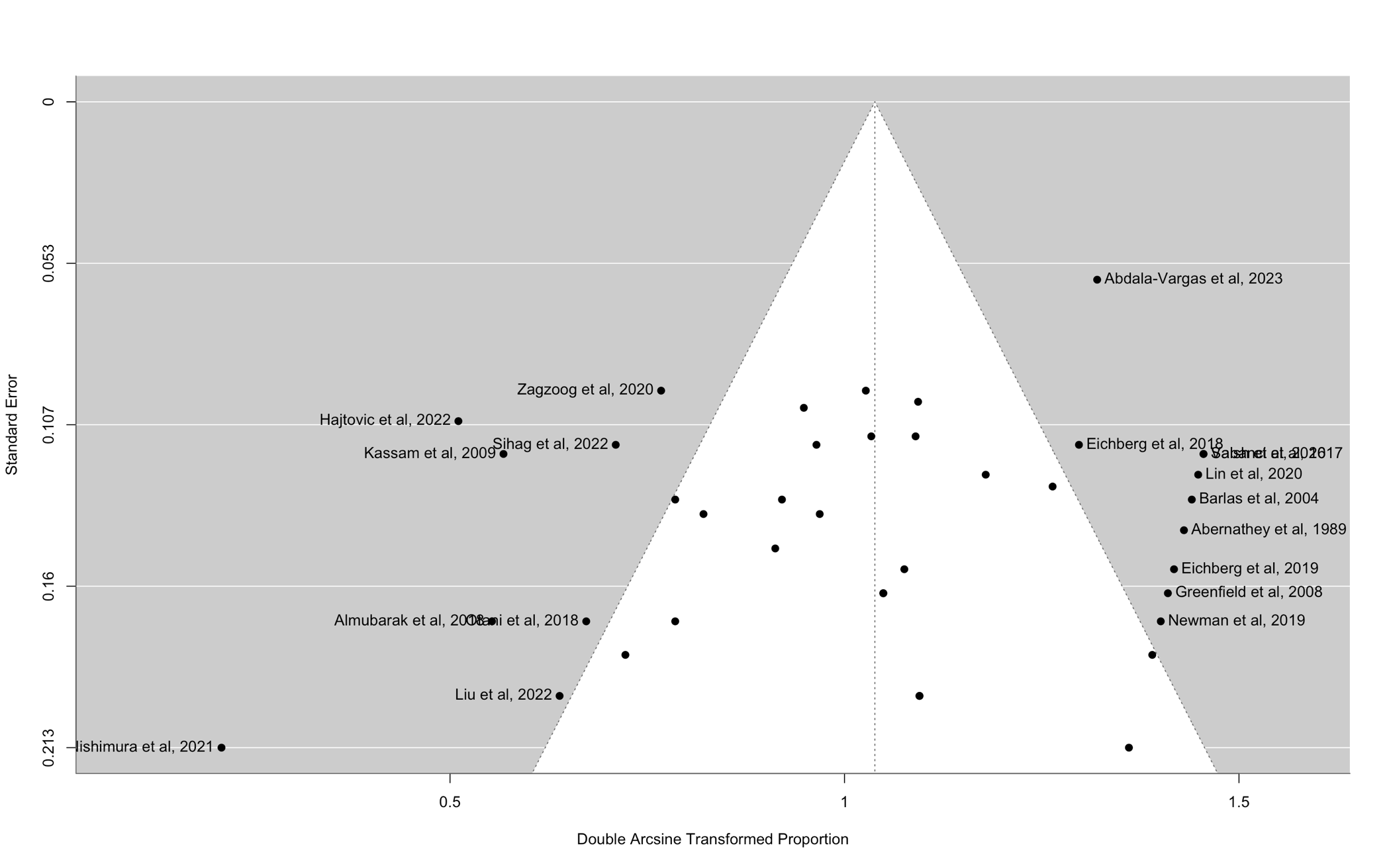


**Overall GTR** (corresponds to Figure 2 forest plot).

Egger’s Test for Funnel Plot Asymmetry: z = -0.8802, p = 0.3788.


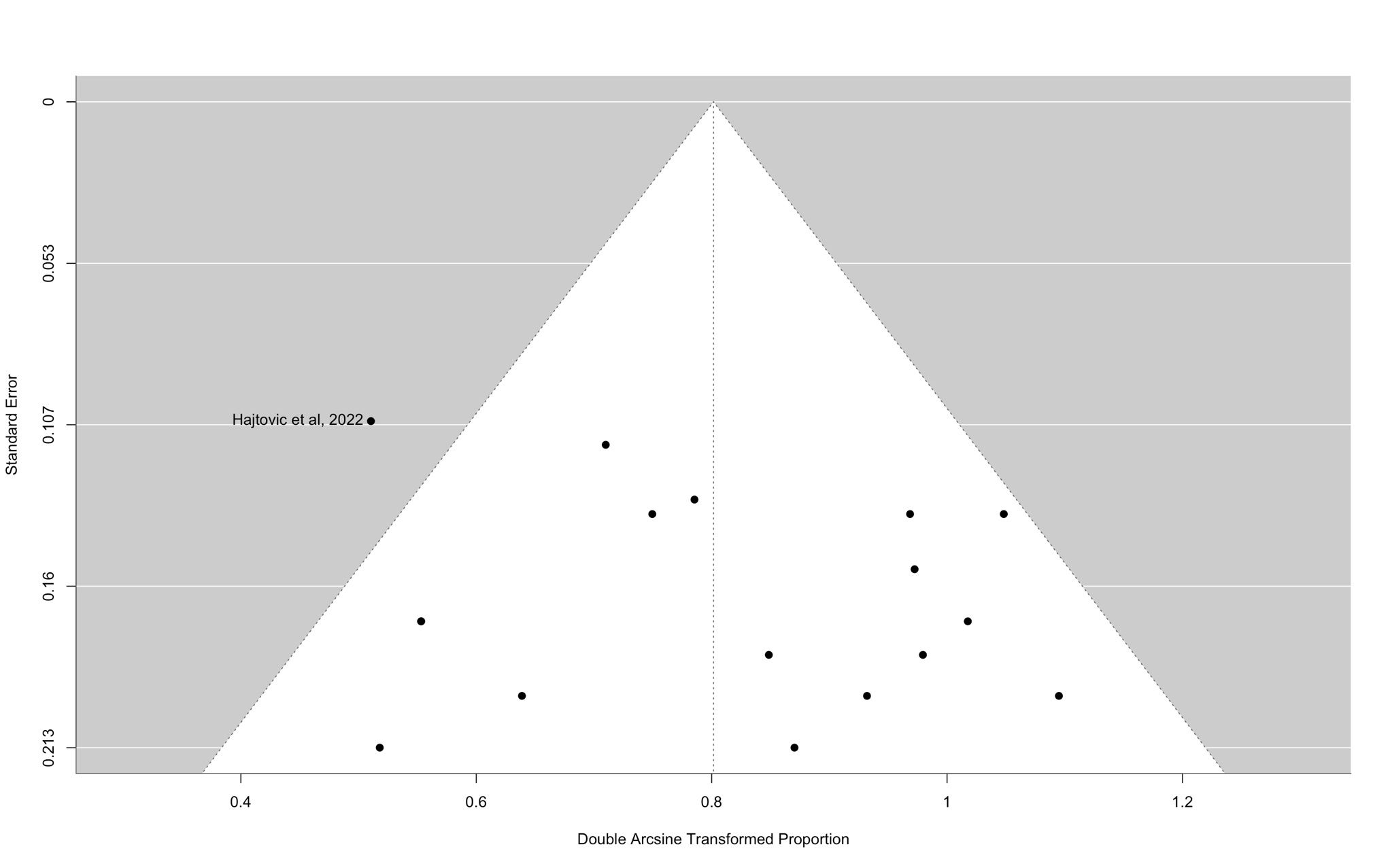


**Gliomas GTR** (corresponds to Figure 3a forest plot).

Egger’s Test for Funnel Plot Asymmetry: z = 0.7463, p = 0.4555.


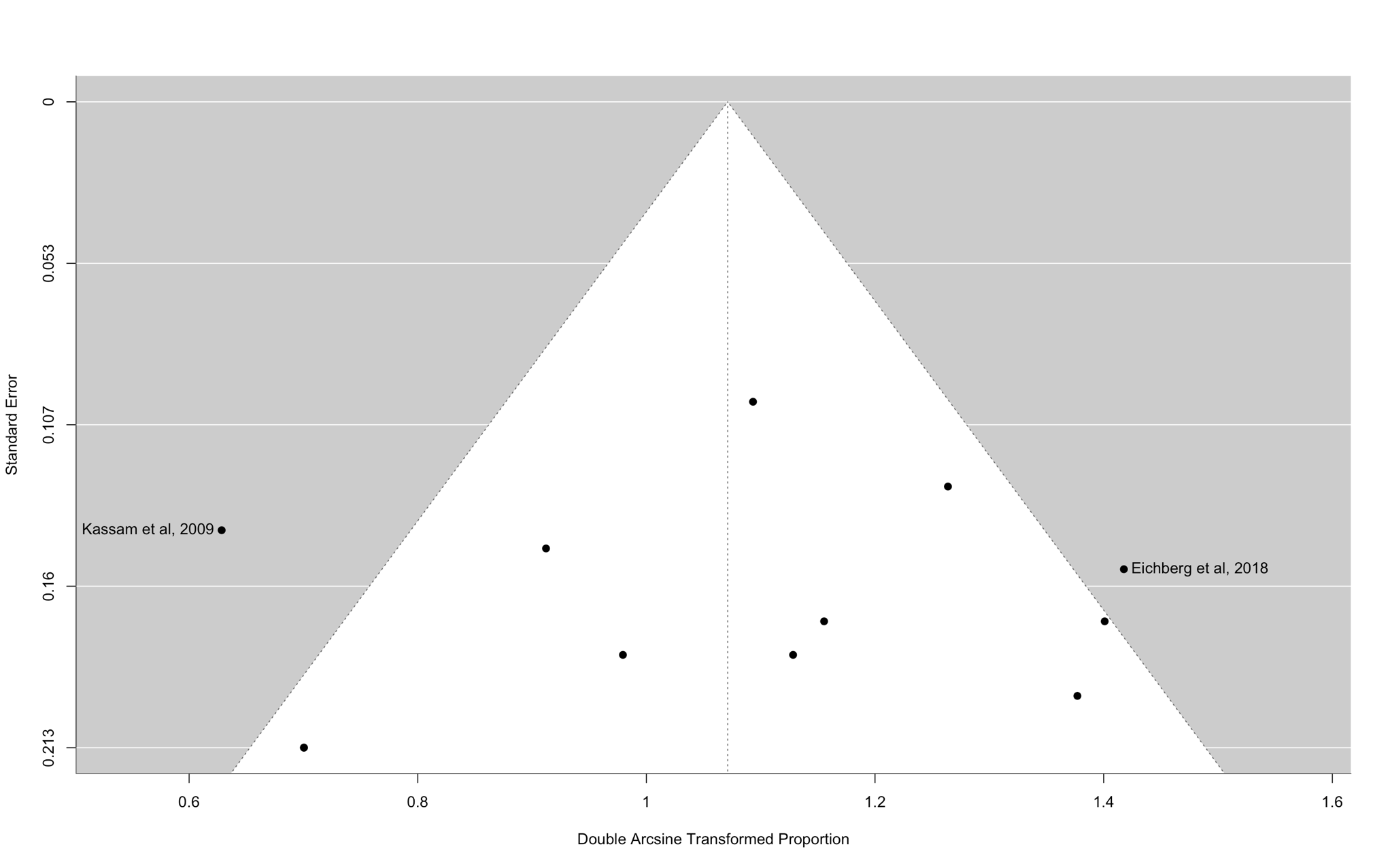


**Metastases GTR** (corresponds to Figure 3b forest plot).

Egger’s Test for Funnel Plot Asymmetry: z = -0.5614, p = 0.5745.


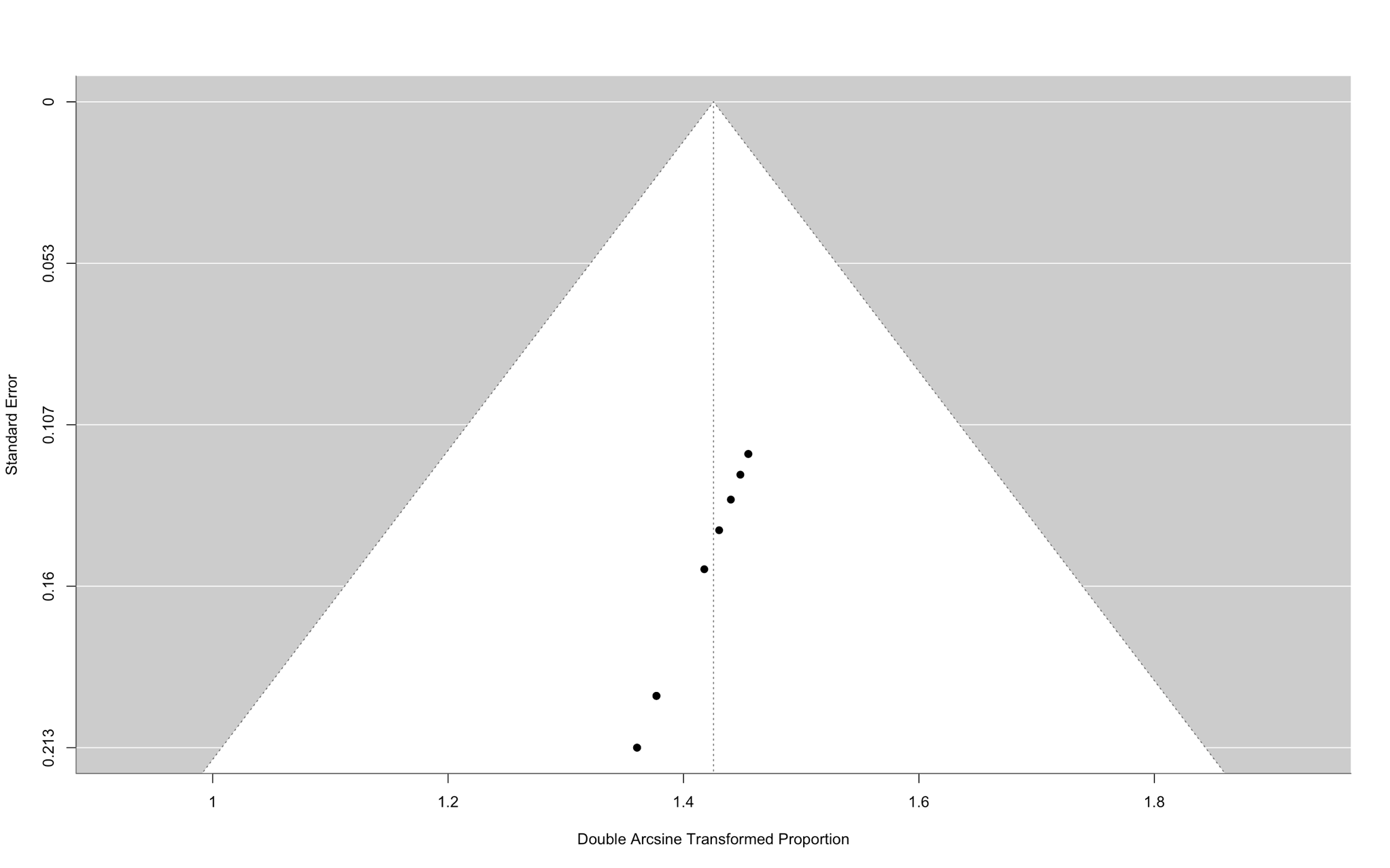


**Colloid cysts GTR** (corresponds to Figure 3c forest plot).

Egger’s Test for Funnel Plot Asymmetry: z = -0.7410, p = 0.4587.


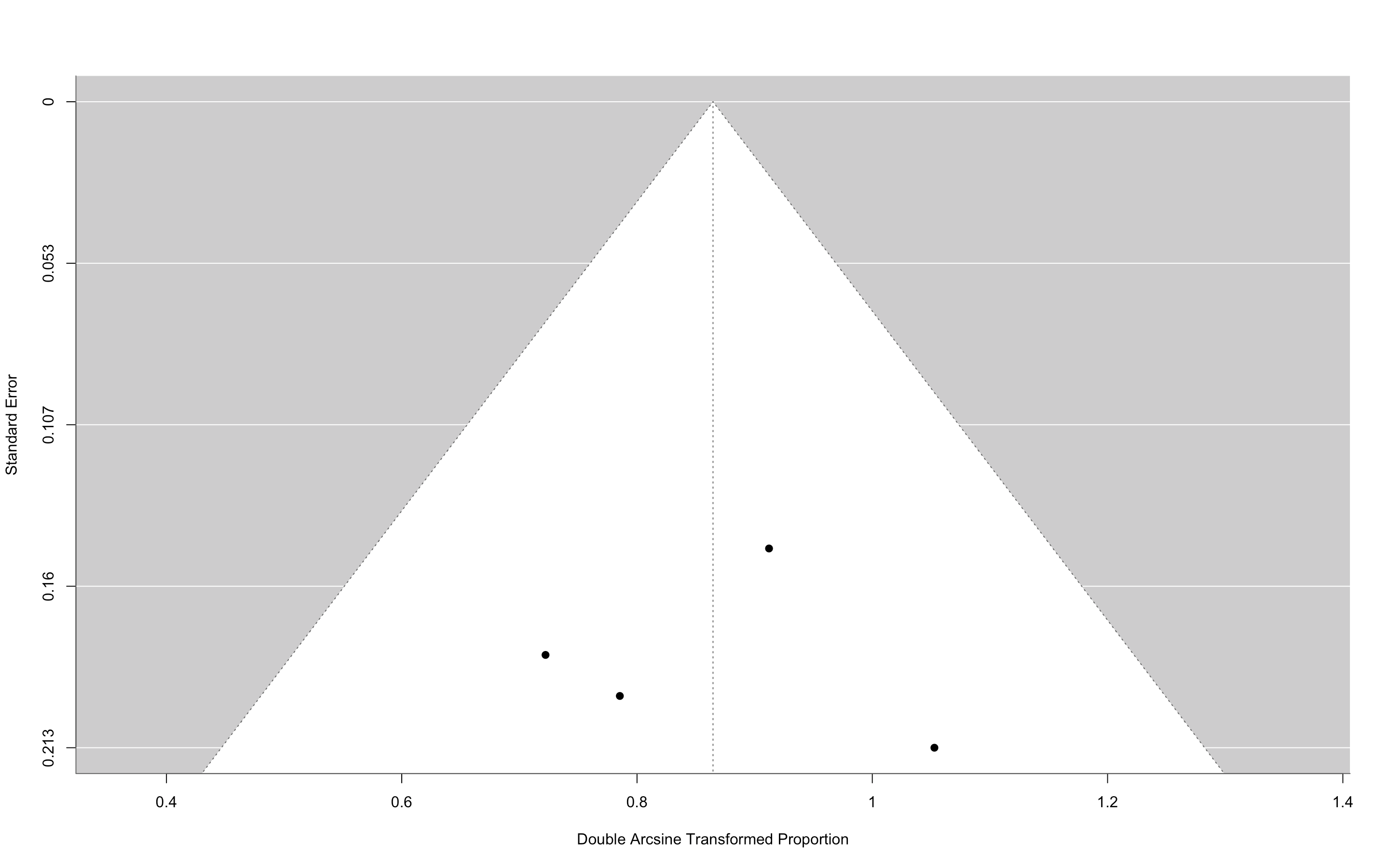


**Low-grade gliomas GTR** (corresponds to Figure 4a forest plot).

Egger’s Test for Funnel Plot Asymmetry: z = 0.0779, p = 0.9379.


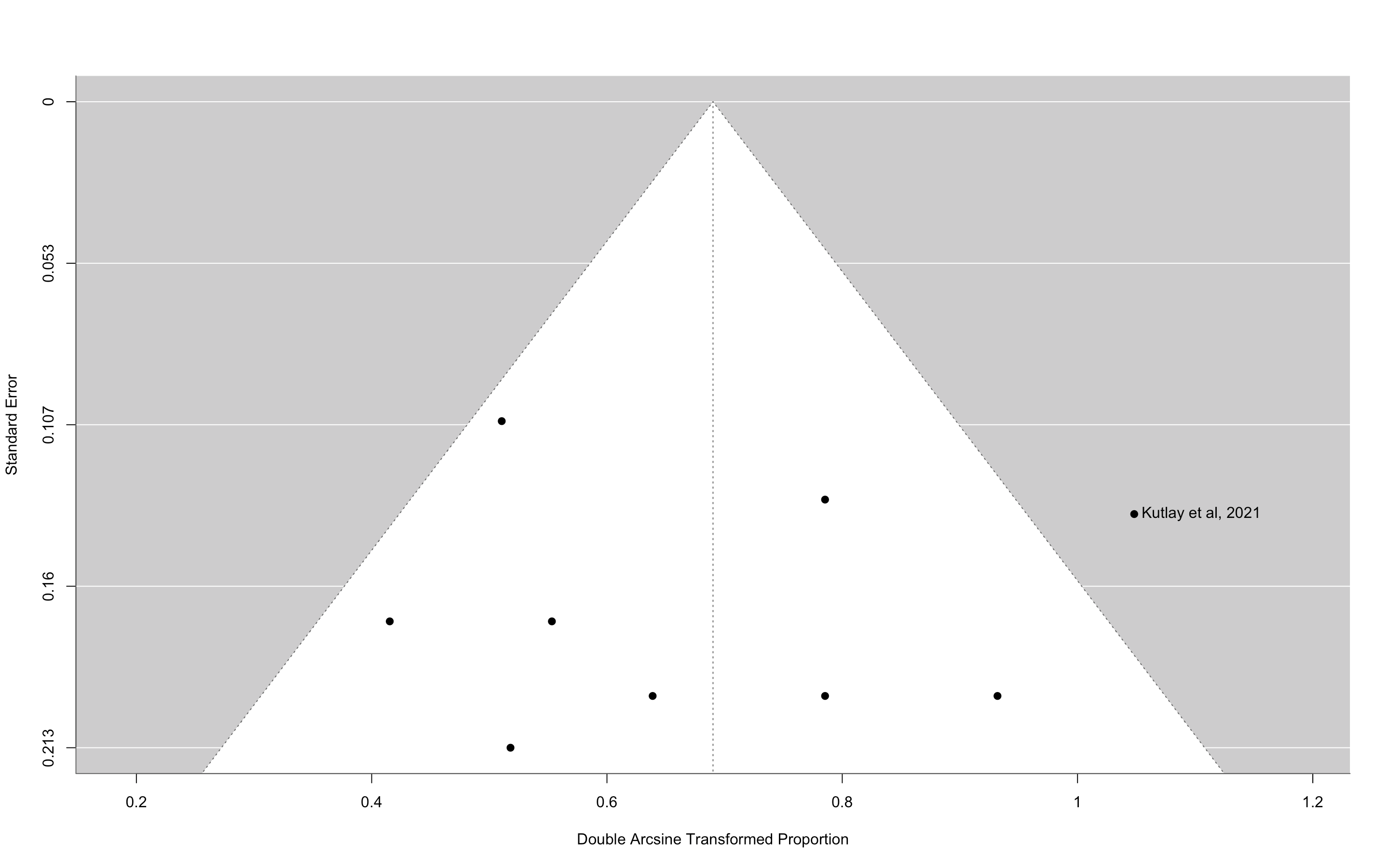


**High-grade gliomas GTR** (corresponds to Figure 4b forest plot).

Egger’s Test for Funnel Plot Asymmetry: z = -0.1077, p = 0.9143.


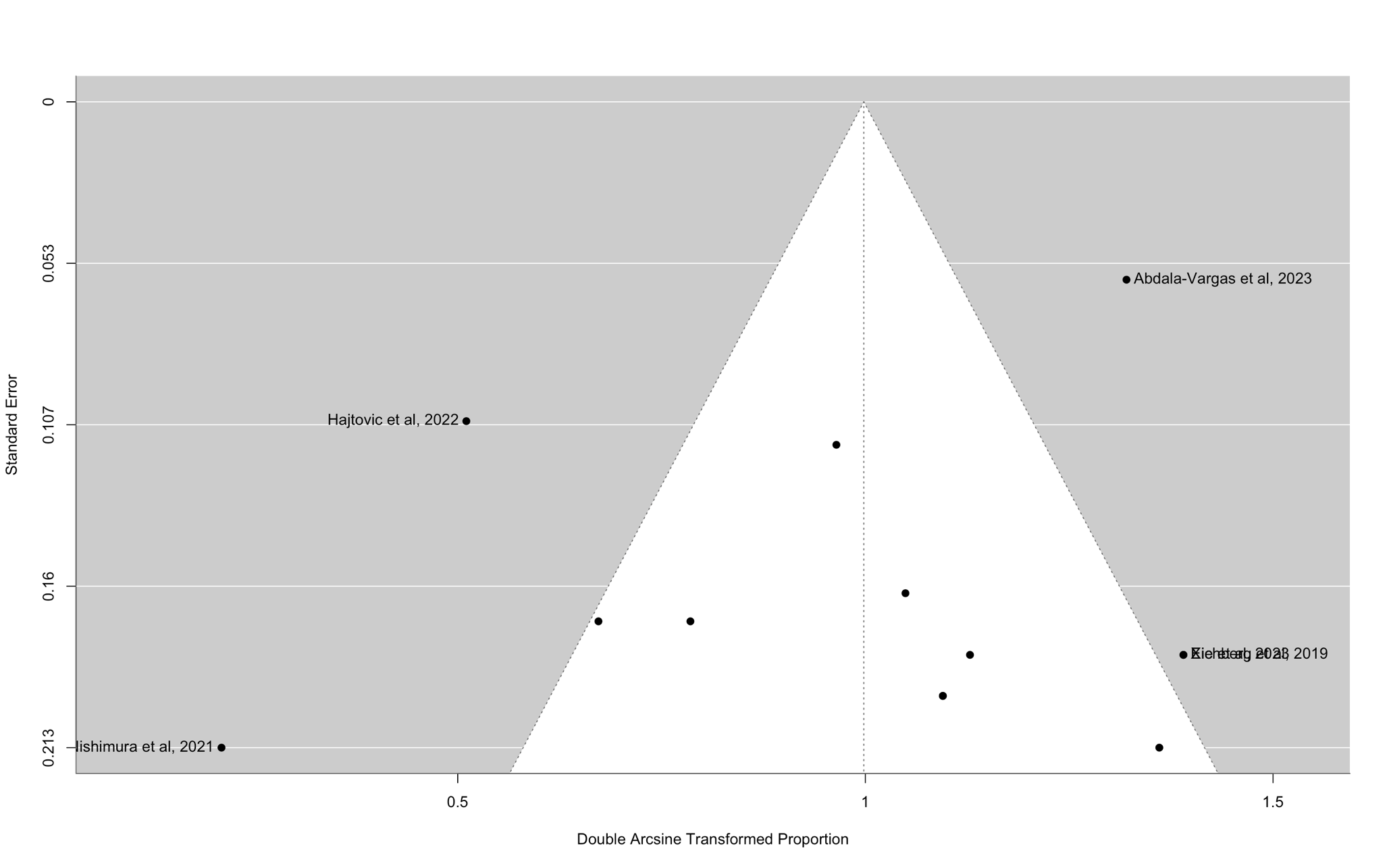


**VBAS GTR** (corresponds to Figure 5a forest plot).

Egger’s Test for Funnel Plot Asymmetry: z = -0.1069, p = 0.9149.


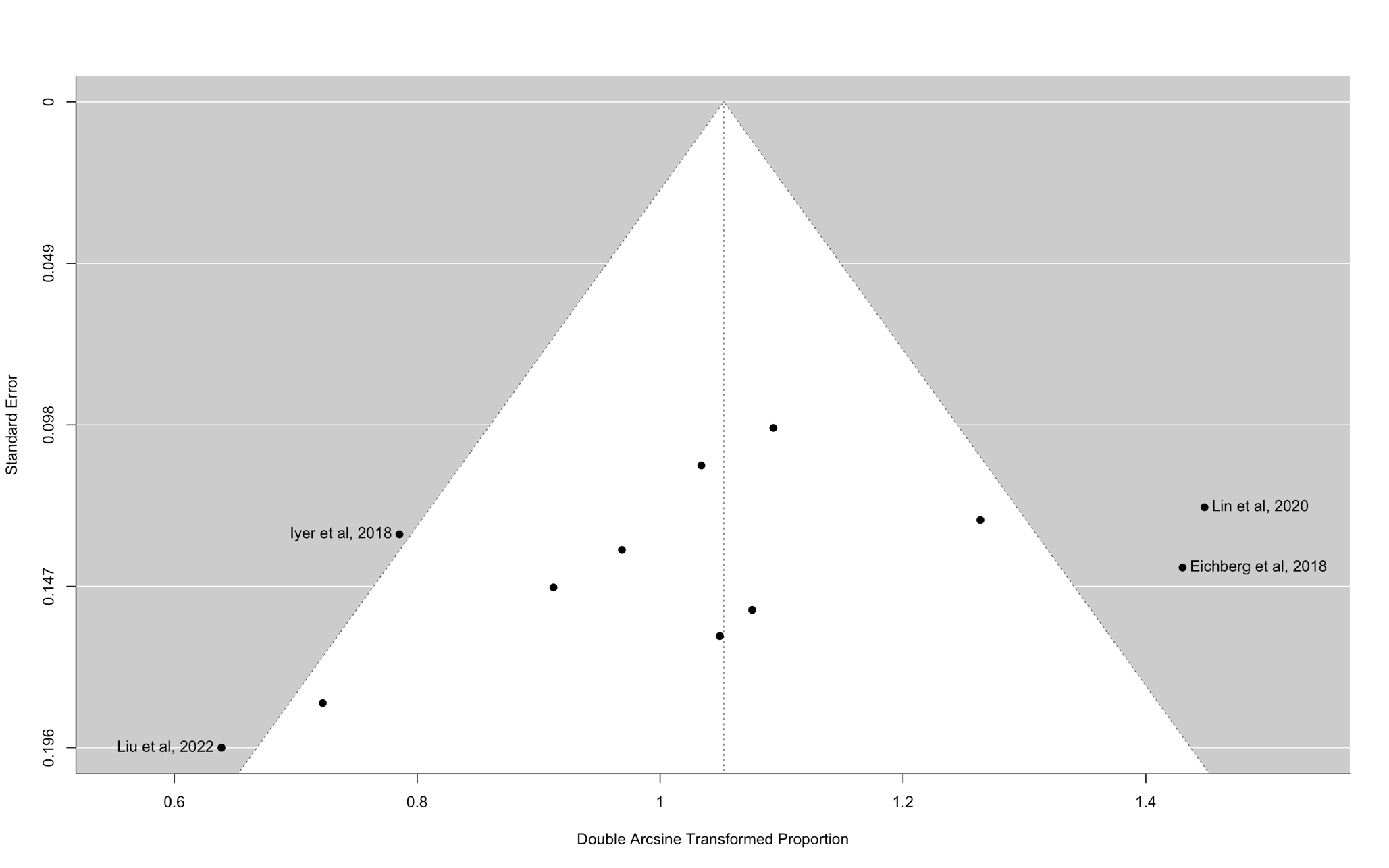


**BrainPath GTR** (corresponds to Figure 5b forest plot).

Egger’s Test for Funnel Plot Asymmetry: z = -1.8105, p = 0.0702.


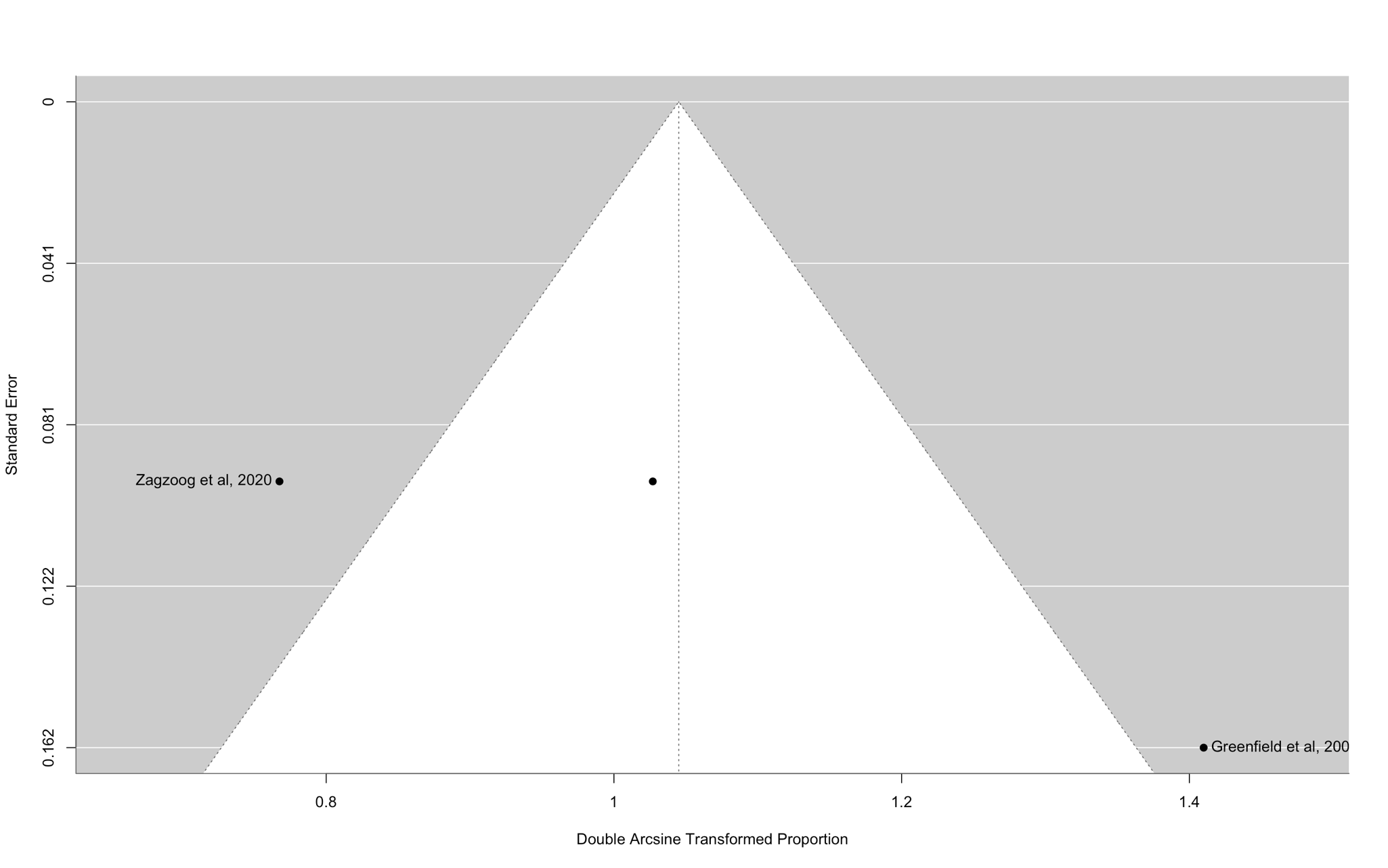


**METRx GTR** (corresponds to Figure 5c forest plot).

Egger’s Test for Funnel Plot Asymmetry: z = 1.9698, p = 0.0489.


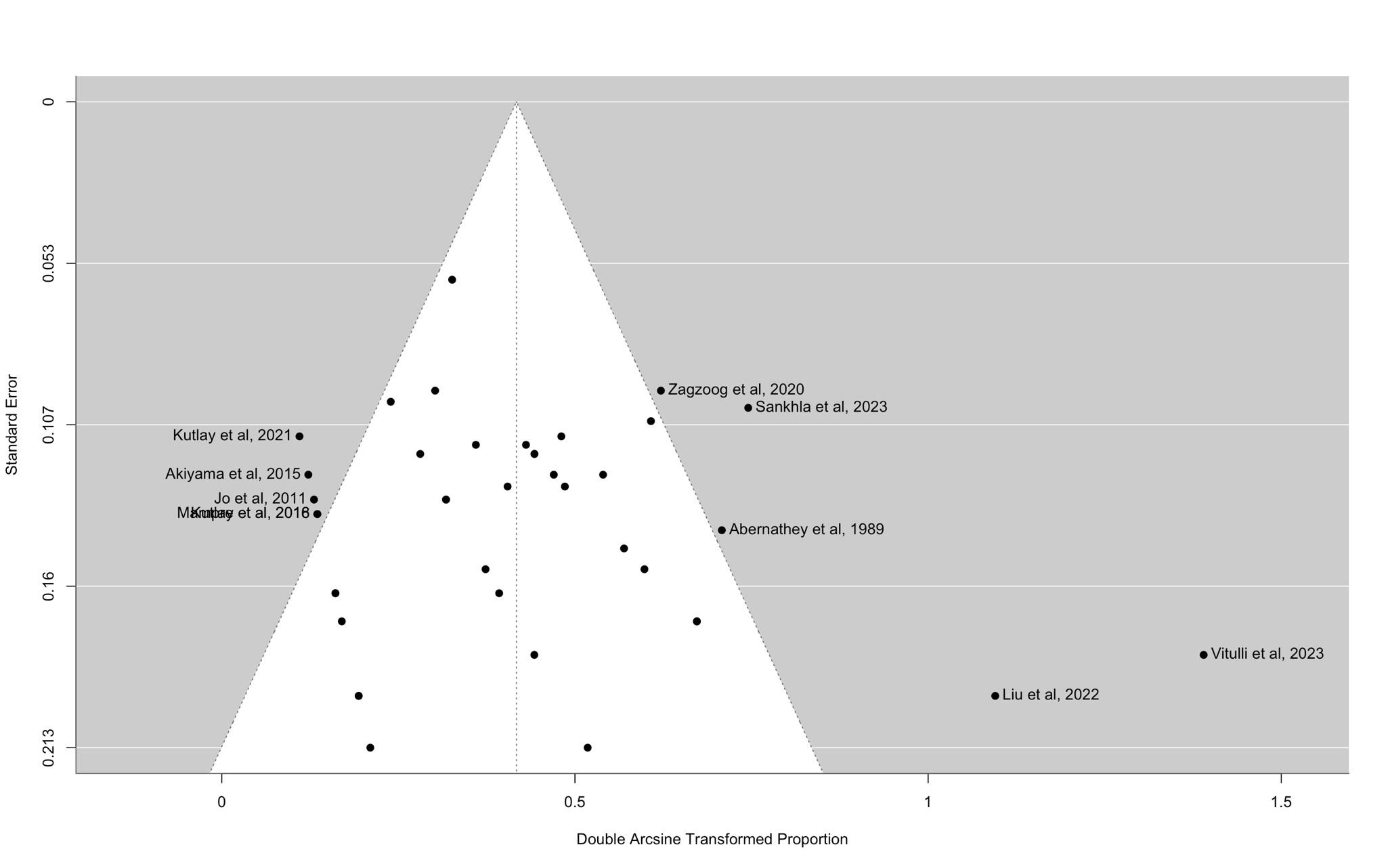


**Overall complications** (corresponds to Figure 6 forest plot).

Egger’s Test for Funnel Plot Asymmetry: z = 0.9806, p = 0.3268.


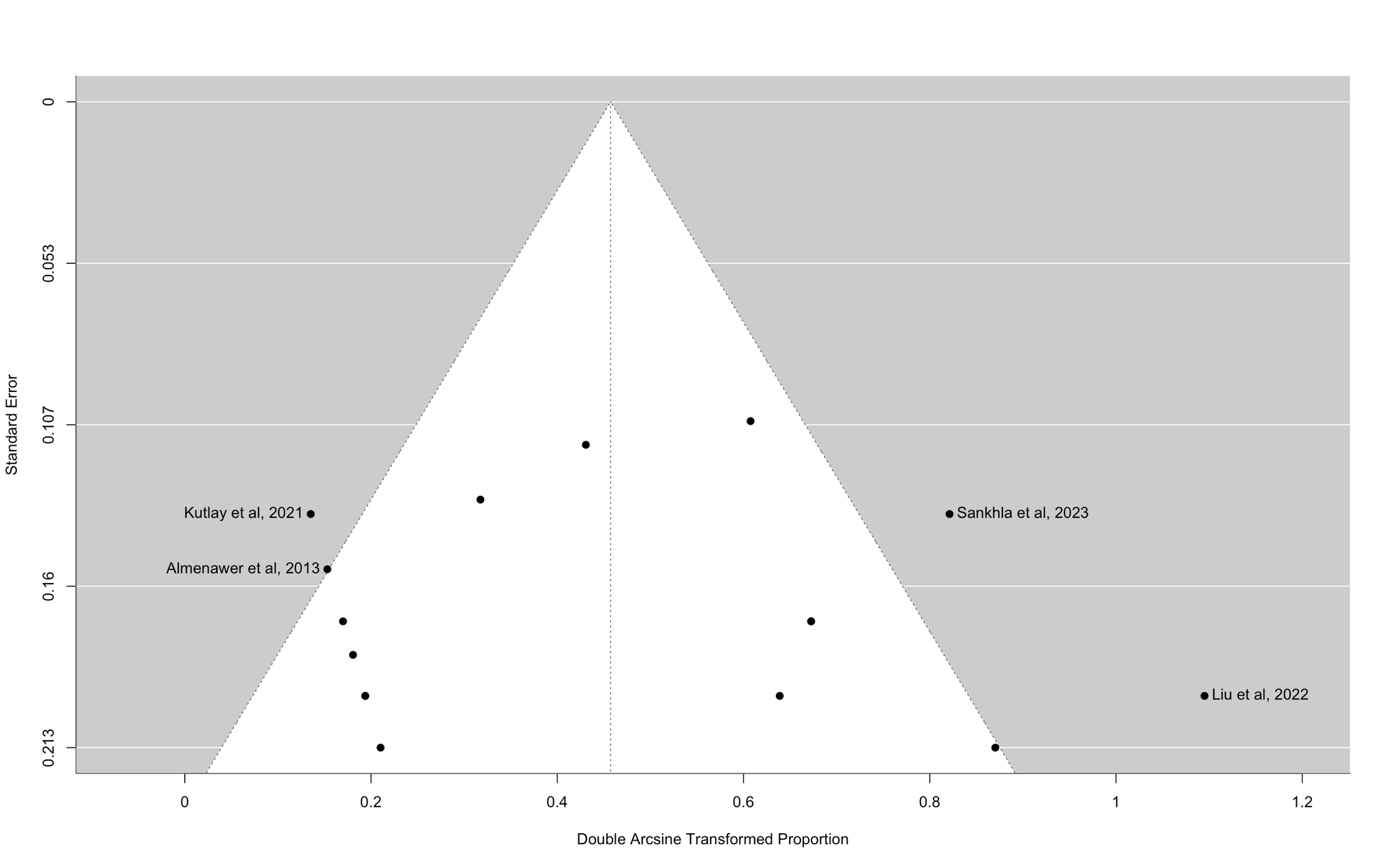


**Gliomas complications** (corresponds to Figure 7a forest plot).

Egger’s Test for Funnel Plot Asymmetry: z = 0.2254, p = 0.8217.


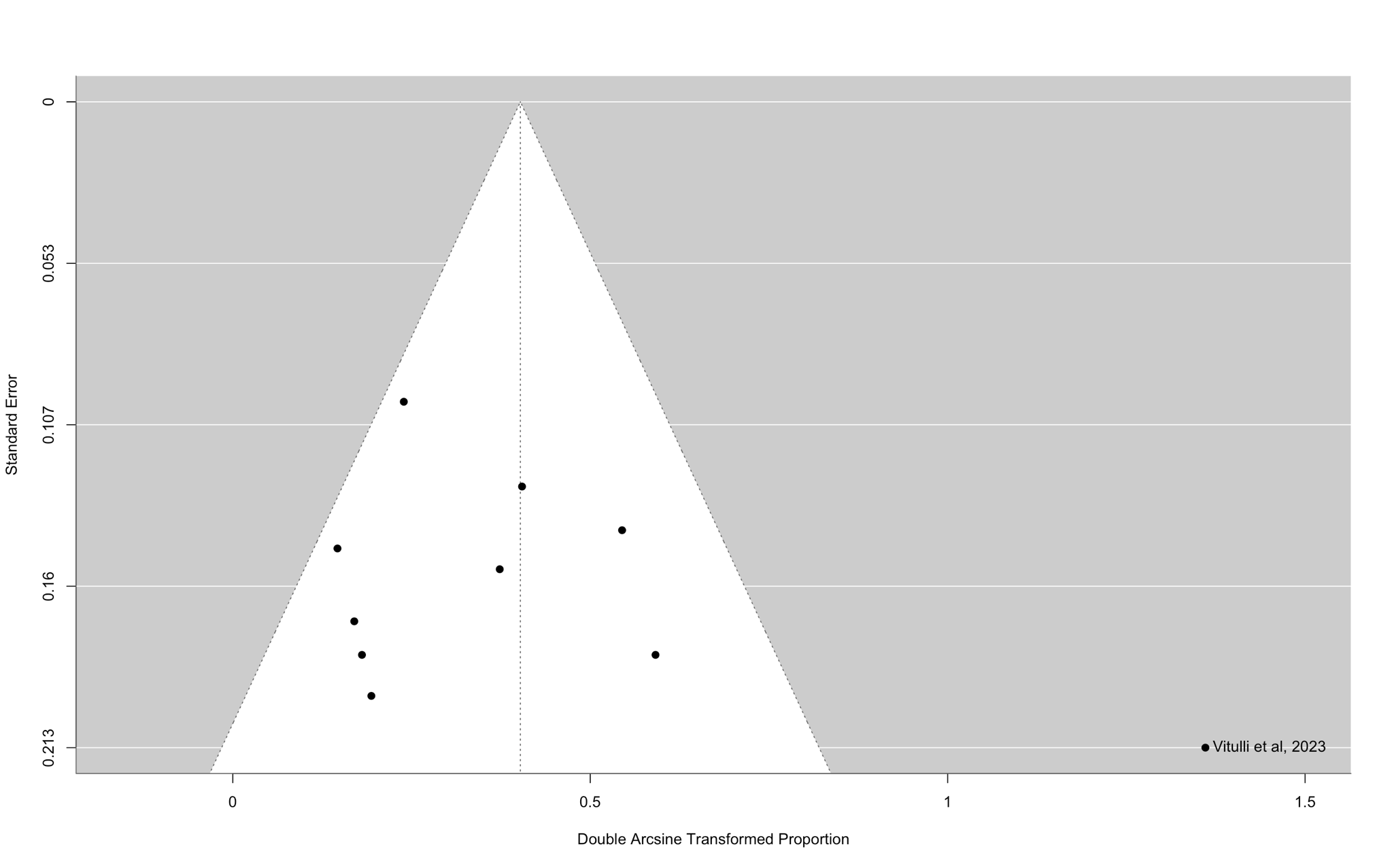


**Metastases complications** (corresponds to Figure 7b forest plot).

Egger’s Test for Funnel Plot Asymmetry: z = 1.4782, p = 0.1394.


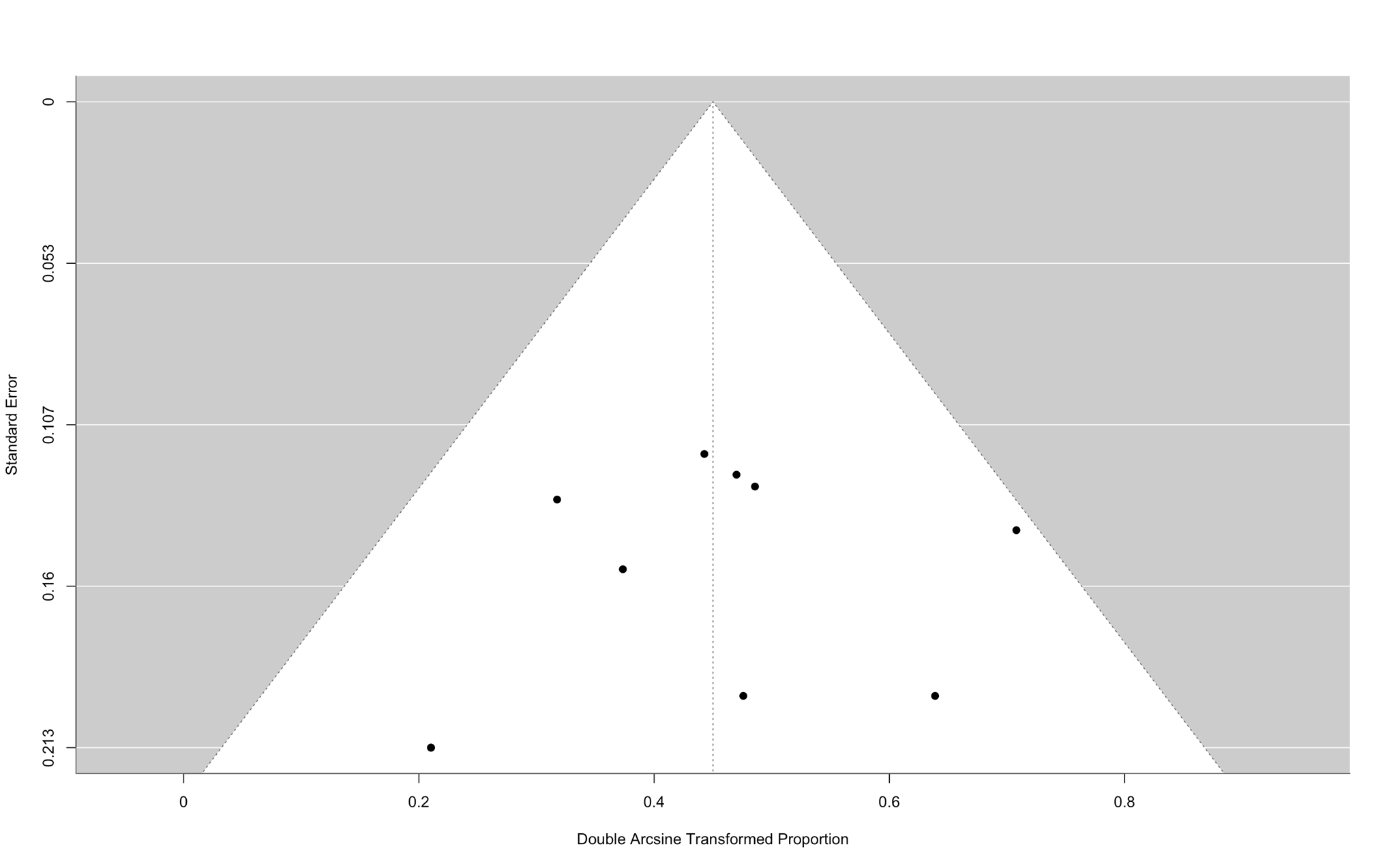


**Colloid cysts complications** (corresponds to Figure 7c forest plot).

Egger’s Test for Funnel Plot Asymmetry: z = -0.6695, p = 0.5032.


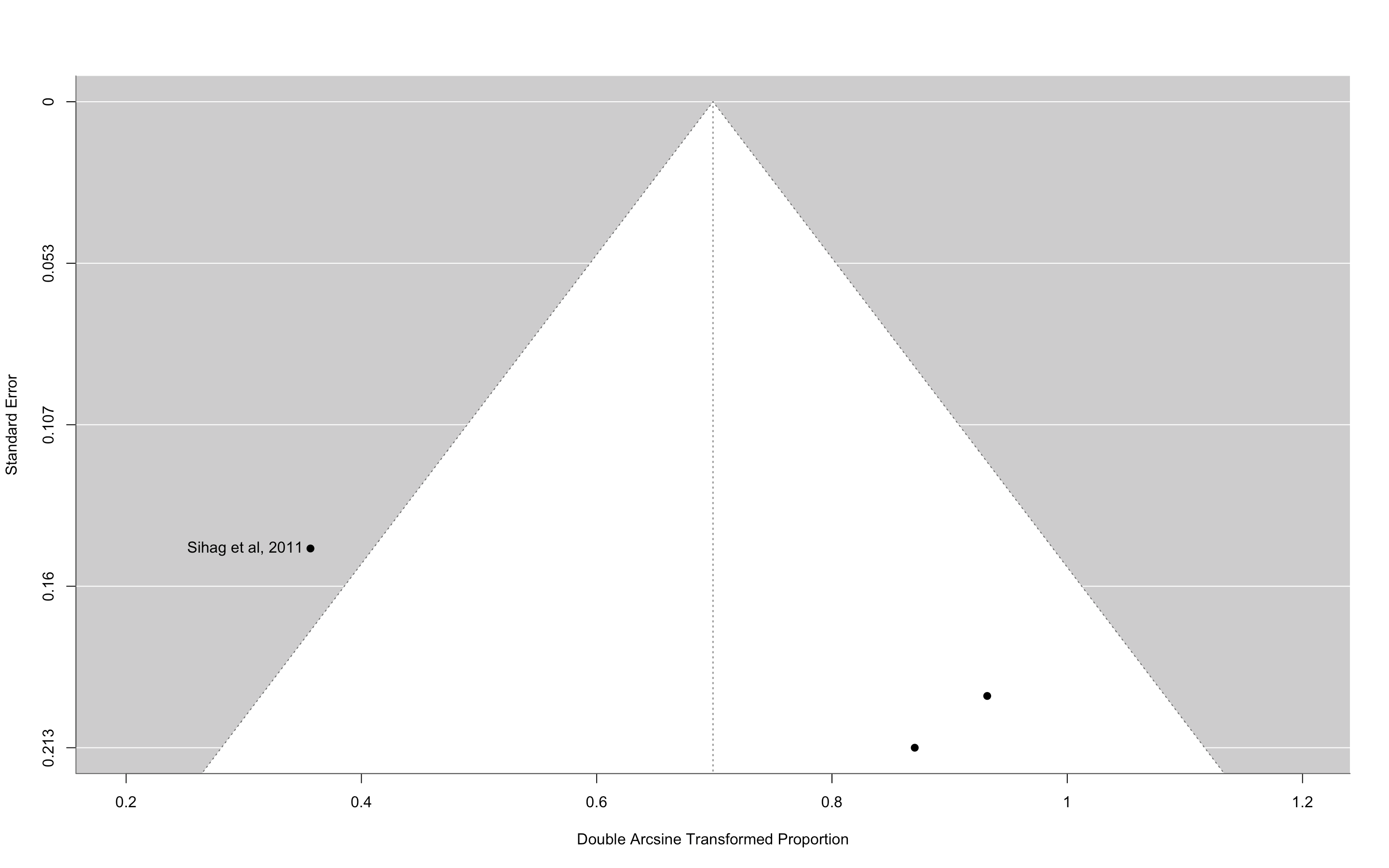


**Low-grade gliomas complications** (corresponds to Figure 8a forest plot).

Egger’s Test for Funnel Plot Asymmetry: z = 2.5471, p = 0.0109.


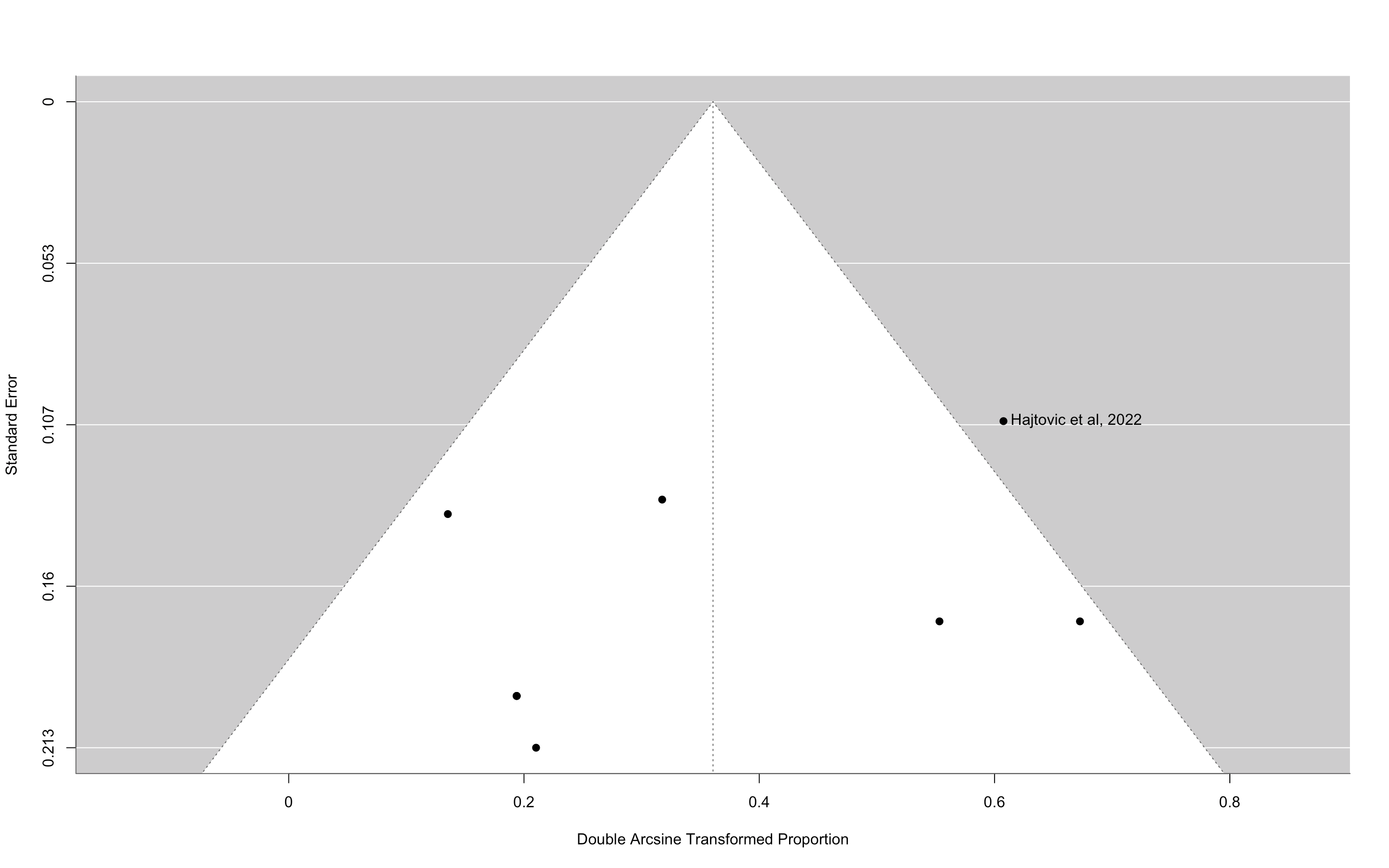


**High-grade gliomas complications** (corresponds to Figure 8b forest plot).

Egger’s Test for Funnel Plot Asymmetry: z = -1.1508, p = 0.2498.


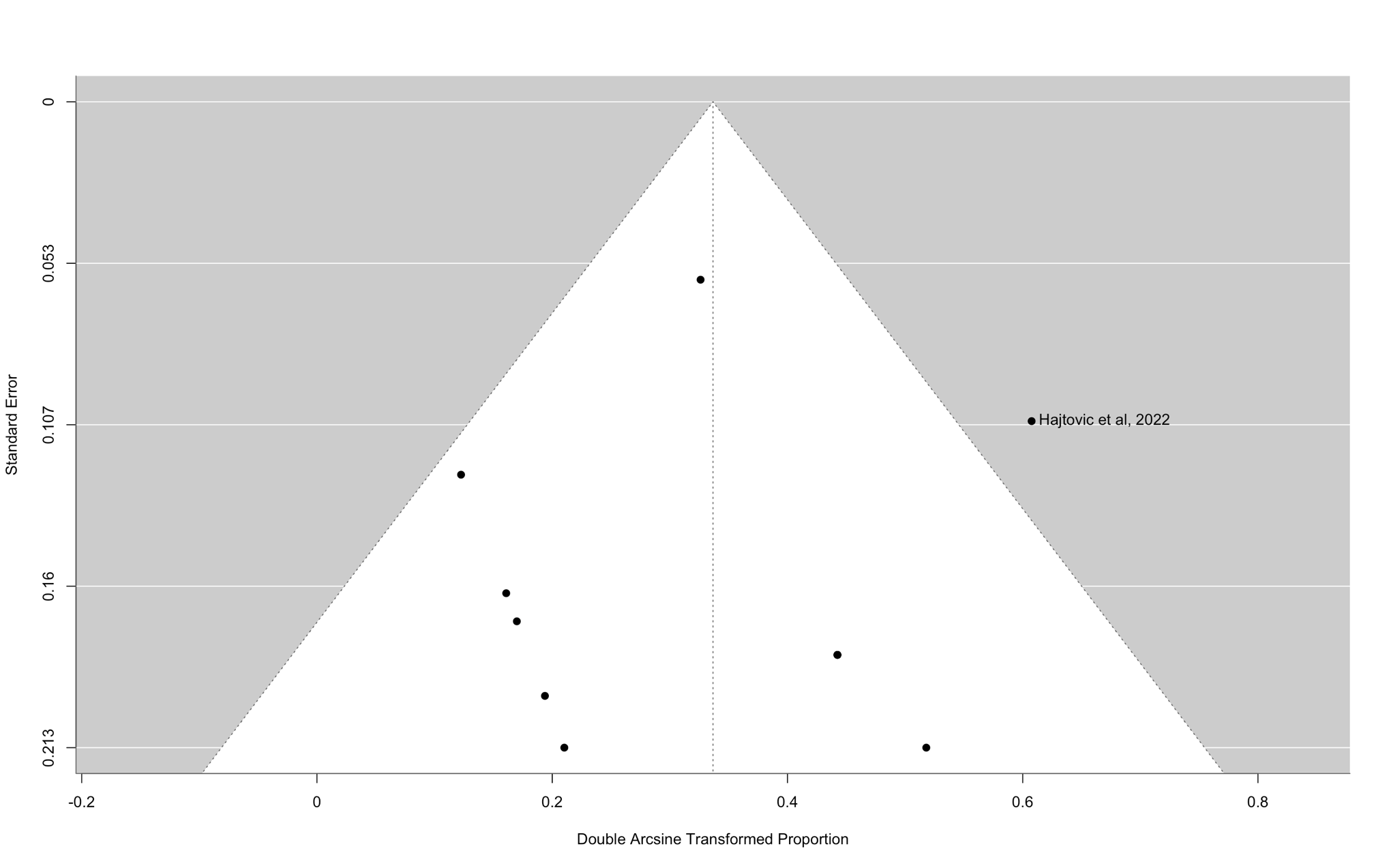


**VBAS complications** (corresponds to Figure 7a forest plot).

Egger’s Test for Funnel Plot Asymmetry: z = -0.2391, p = 0.8110.


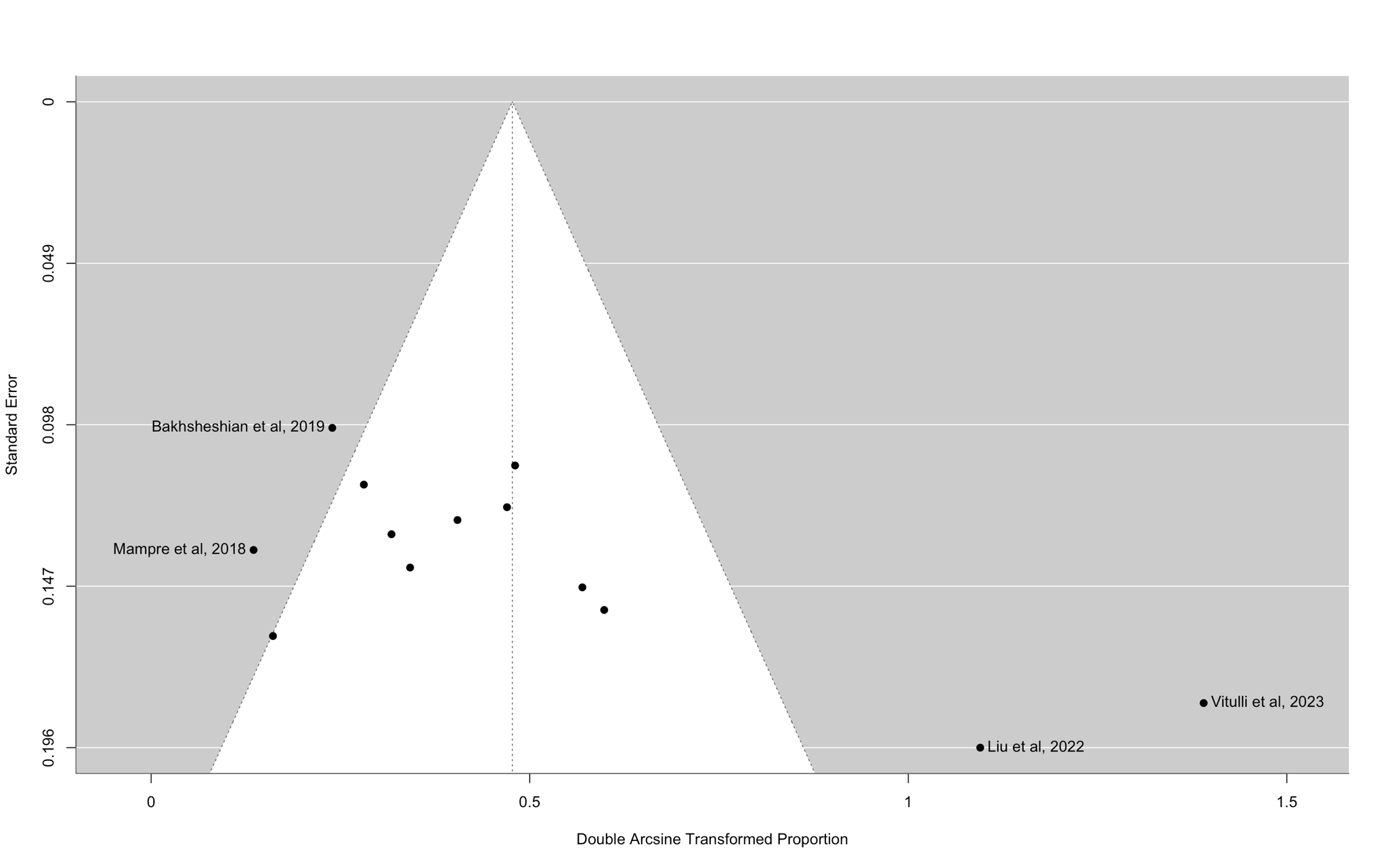


**BrainPath complications** (corresponds to Figure 7b forest plot).

Egger’s Test for Funnel Plot Asymmetry: z = 3.3538, p = 0.0008.


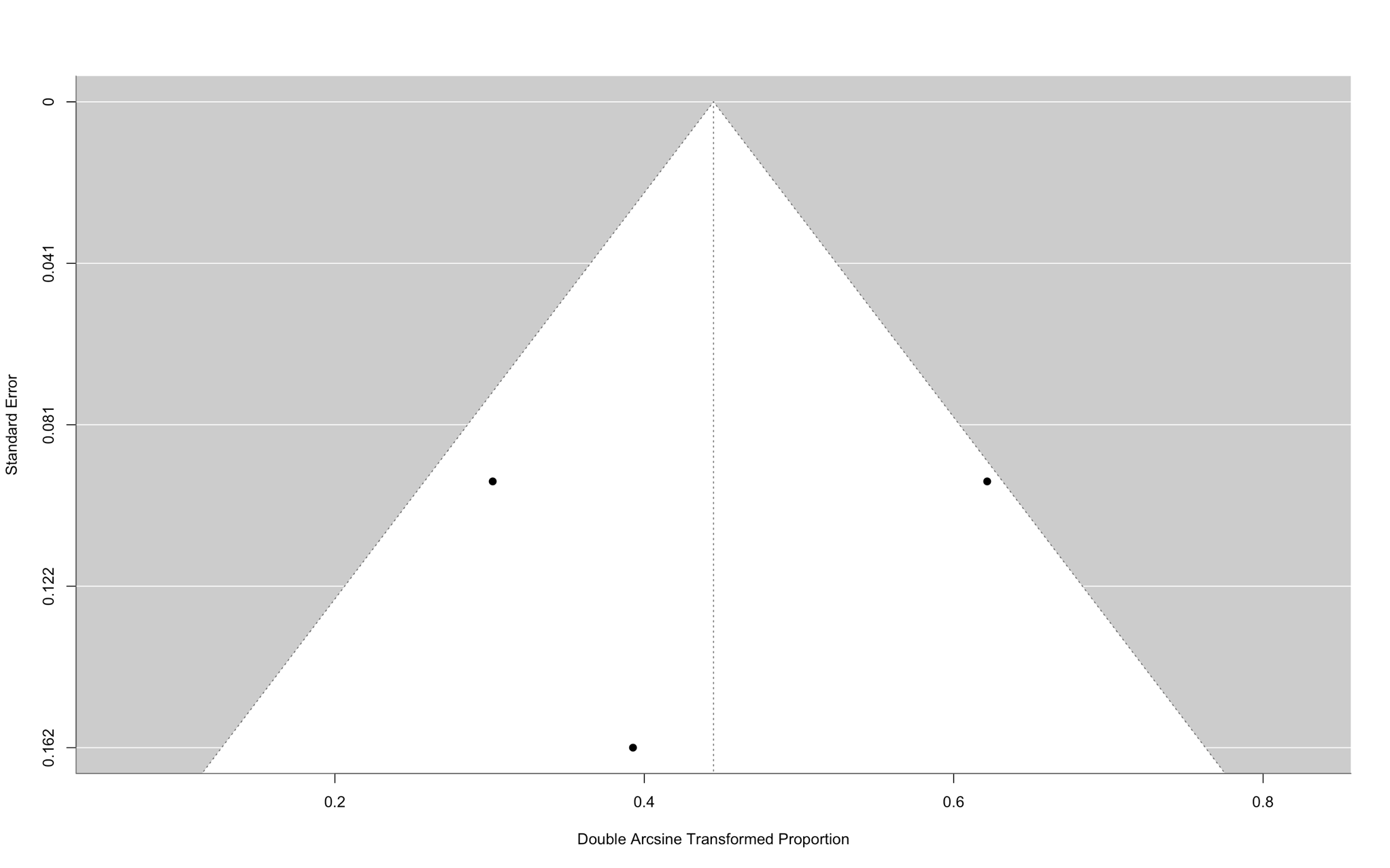


**METRx complications** (corresponds to Figure 7c forest plot).

Egger’s Test for Funnel Plot Asymmetry: z = -0.2257, p = 0.8215.

**Supplementary Data 11 – Certainty of Evidence Assessment**

**Summary and certainty of evidence.**

*CI = confidence interval; GTR = gross total resection; METRx = minimal exposure tubular retractor system; VBAS = viewsite brain access system.*

| **Outcomes** | **Number of patients (studies)** | **Pooled prevalence estimate (95% CI)** | **Quality of the evidence (GRADE)** |
| --- | --- | --- | --- |
| Combined tubular retractor GTR rate | 607 patients  (40 studies) | 76%  (67-85%) | ⊝⊝⊝⊝  Very low  Due to case series design, and inconsistency of results |
| Combined tubular retractor complication rate | 611 patients  (40 studies) | 14%  (8-20%) | ⊝⊝⊝⊝  Very low  Due to case series design, and inconsistency of results |
| Gliomas GTR rate | 170 patients  (17 studies) | 52%  (41-62%) | ⊝⊝⊝⊝  Very low  Due to case series design |
| Metastases GTR rate | 119 patients  (12 studies) | 80%  (65-92%) | ⊝⊝⊝⊝  Very low  Due to case series design, and inconsistency and imprecision of results |
| Colloid cysts GTR rate | 116 patients  (11 studies) | 100%  (99-100%) | ⊝⊝⊝⊝  Very low  Due to case series design |
| Low-grade gliomas GTR rate | 29 patients  (4 studies) | 59%  (39-78%) | ⊝⊝⊝⊝  Very low  Due to case series design |
| High-grade gliomas GTR rate | 88 patients  (9 studies) | 39%  (24-56%) | ⊝⊝⊝⊝  Very low  Due to case series design |
| Gliomas complication rate | 156 patients  (16 studies) | 16%  (5-30%) | ⊝⊝⊝⊝  Very low  Due to case series design, and inconsistency of results |
| Metastases complication rate | 106 patients  (10 studies) | 12%  (1-28%) | ⊝⊝⊝⊝  Very low  Due to case series design, and inconsistency of results |
| Colloid cysts complication rate | 107 patients  (10 studies) | 16%  (8-24%) | ⊝⊝⊝⊝  Very low  Due to case series design |
| Low-grade gliomas complication rate | 22 patients  (3 studies) | 40%  (4-83%) | ⊝⊝⊝⊝  Very low  Due to case series design, inconsistency and imprecision of results, and publication bias |
| High-grade gliomas complication rate | 88 patients  (9 studies) | 8%  (1-20%) | ⊝⊝⊝⊝  Very low  Due to case series design |
| VBAS GTR rate | 181 patients  (13 studies) | 73%  (51-91%) | ⊝⊝⊝⊝  Very low  Due to case series design, and inconsistency and imprecision of results |
| BrainPath GTR rate | 158 patients  (12 studies) | 78%  (64-89%) | ⊝⊝⊝⊝  Very low  Due to case series design, and inconsistency of results |
| METRx GTR rate | 63 patients  (3 studies) | 76%  (44-98%) | ⊝⊝⊝⊝  Very low  Due to case series design, inconsistency and imprecision of results, and publication bias |
| VBAS complication rate | 164 patients (11 studies) | 7%  (1-15%) | ⊝⊝⊝⊝  Very low  Due to case series design |
| BrainPath complication rate | 176 patients  (13 studies) | 19%  (7-34%) | ⊝⊝⊝⊝  Very low  Due to case series design, inconsistency of results, and publication bias |
| METRx complication rate | 63 patients (3 studies) | 17%  (3-37%) | ⊝⊝⊝⊝  Very low  Due to case series design, and inconsistency and imprecision of results |
